# Social determinants of health and epigenetic clocks: a systematic review and meta-analysis of 140 studies

**DOI:** 10.1038/s41562-026-02477-6

**Published:** 2026-06-12

**Authors:** Y. E. Willems, A. D. Rezaki, M. Aikins, A. Bahl, Q. Wu, D. W. Belsky, L. Raffington

**Affiliations:** 1 Max Planck Research Group Biosocial – Biology, Social Disparities and Development, Max Planck Institute for Human Development, Berlin, Germany; 2 Robert N Butler Columbia Aging Center and Department of Epidemiology, Columbia University Mailman School of Public Health, New York, NY USA

**Keywords:** Predictive markers, Human behaviour, Social policy

## Abstract

Social determinants of health—such as socioeconomic status (SES) and race and ethnicity—strongly shape health. Individuals with lower SES or marginalized identities experience earlier onset of disease and shorter lifespans. ‘Epigenetic clocks’ measure biological ageing and are increasingly used to study healthy ageing, yet it remains unclear which clocks are most sensitive to social inequality. Here we conducted an Open Science Framework-pre-registered systematic review and meta-analysis of 140 studies (*N* = 65,919; 1,065 effect sizes) testing associations of SES and race and ethnicity with three generations of clocks. PubMed, PsycINFO, Web of Science, medRxiv and bioRxiv were searched from February 2024 to September 2025. Eligible studies included empirical articles in English published since 2013 on non-clinical populations reporting at least one relevant association. Associations between SES and biological ageing varied across different generations of epigenetic clocks (*F*(2) = 178,10, *P* < 0.001), with the weakest effects for first-generation clocks (*r* = –0.03, 95% confidence intervaI (CI) [−0.04, −0.01], *P* < 0.001) and stronger effects for second- (*r* = –0.11, 95% CI [−0.12, −0.09], *P* < 0.001) and third-generation clocks (*r* = –0.13, 95% CI [−0.15, −0.11], *P* < 0.001]). Sex, tissue and array minimally modified results, and publication bias was negligible. Limitations include inconsistent reporting of technicalities and overrepresentation of data from high-income countries. The findings indicate that newer clocks are more responsive to social inequality.

## Abstract (web summary)

This meta-analysis of 140 studies (~66,000 participants) finds that social inequality is associated with faster biological ageing, with stronger associations observed for newer DNA methylation epigenetic clocks than earlier generations.

## Main

Individuals with lower socioeconomic status (SES) or marginalized racial and ethnic identities often experience an earlier onset of chronic diseases and a shorter lifespan compared with their more advantaged counterparts^1,2^. These factors are considered fundamental because they shape exposure to a wide range of health-relevant conditions, including access to resources, environmental hazards and chronic stress^3–5^. One hypothesis advanced to explain these disparities is that social disadvantage accelerates processes of biological ageing^6–10^. Biological ageing is the progressive loss of integrity and resilience capacity in cells, tissues and organs with the passage of time^8,11^. While there is no gold-standard measurement of biological ageing^11,12^, a family of machine-learning-derived DNA methylation (DNAm) algorithms known as ‘epigenetic clocks’ are widely studied and well-validated biomarker predictors of healthy lifespan across a range of human populations^13,14^. These algorithms are emerging as an important tool in social determinants of health research, where researchers are using them to track the biological embedding of health risks.

Epigenetic clocks are developed by modelling proxy measures of biological ageing from whole-genome DNAm patterns. The result is a predictive algorithm that, when applied to DNAm data from a new sample, returns a numeric value representing the progress or pace of biological ageing. Within this general approach, there are now multiple ‘generations’ of clocks^6,15^. The first generation of epigenetic clocks was developed by modelling differences between individuals in their chronological age (that is, time since birth)^16,17^. First-generation epigenetic clocks estimate biological age as the age at which a person’s DNAm pattern matches the norm in a reference population. While first-generation clocks accurately estimate chronological age, their ability to predict health outcomes appears to be more limited^18^. To overcome this limitation, the second generation of epigenetic clocks was developed by modelling differences between individuals in their risk of death and translating these risks into age-equivalent values. Second-generation epigenetic clocks estimate biological age as the age at which an individual’s predicted mortality and health risk match the norm in a reference population^19,20^. A third generation of epigenetic clocks was developed by modelling differences between individuals in the rate at which their bodies deteriorate over time. Rather than estimating a biological age, they estimate a person’s pace of ageing, or the rate at which biological age is increasing relative to the norm in a reference population^21,22^.

Research increasingly shows that epigenetic clocks are sensitive to socioeconomic and racial and ethnic disparities^7,23–26^. However, many clocks exist, and they do not provide identical information. In fact, correlations across generations of clocks are modest^24,27^. Key knowledge gaps include which clocks best capture social determinants of health, when socioeconomic exposures most affect biological ageing and whether effects differ by sex. Theory and evidence suggest that ageing begins at conception^28,29^. However, how molecular damage and hallmarks of ageing relate to early-life exposures remains unclear. Addressing these questions will improve the use of clocks in studies testing interventions across the life course.

We conducted a pre-registered systematic review and multi-level meta-analysis synthesizing findings on the associations of SES and race and ethnicity with three generations of epigenetic-clock measures of biological ageing (see Table 1; https://osf.io/u5z82). First, we examined SES associations with epigenetic clocks. Second, we investigated whether these associations varied depending on when in life (childhood versus adulthood) SES and DNAm were measured. Third, we examined race and ethnicity associations with epigenetic clocks. Fourth, we explored potential factors modifying the associations, such as sex and technical factors (tissue type, array platform, and included covariates, such as body mass index (BMI) and smoking; Table 2), and whether there was evidence of publication bias.

**Table 1:** Overview of key concepts of interest for epigenetic clock-measured biological ageing and social determinants of health

Epigenetic clock-measured biological ageing	Social determinants of health
First generation measures: - Horvath Pan-Tissue Clock^17^ - Hannum Clock^16^ - Horvath Skin and Blood Clock^82^ - Zhang Clock^83^ - PedBE Clock^84^ Second generation measures: - PhenoAge^19^ - GrimAge^20,85^ Third generation measures: - DunedinPoAm^21^ - DunedinPACE^22^	Socioeconomic status in adulthood: - Income/wealth - Education - Occupational status - Composite measures that combine two or more of these variables into one SES measure - Neighbourhood SES Socioeconomic status in childhood: - Parental income/wealth - Parental education - Parental occupation - Composite measures that combine two or more of these variables into one SES measure - Neighbourhood SES Race and ethnicity - Self-reported racial and ethnic identity

Although SES was operationalized differently across studies (for example, education, income and composite indices), these indicators are conceptually and empirically related and were therefore analysed together, consistent with previous meta-analytic work on SES and epigenetic ageing^8,9^.

**Table 2:** Overview of moderator variables that were explored in this meta-analysis

Moderator	Type	Measure	Levels
First, second and third generation epigenetic clocks	Categorical	Epigenetic clock measures	Horvat Pan-Tissue, Hannum, Horvath Skin & Blood, Zhang, PedBE, PhenoAge, GrimAge, GrimAge2, DunedinPoAm, DunedinPACE
Tissue in target sample	Categorical	Tissue from which DNAm was extracted	Venous blood, blood spot, cord blood, saliva, buccal tissue
Age at DNAm sampling	Continuous and categorical	Timing of DNAm data collection	Prenatal (0–0.5), early childhood (0.5–5), late childhood (5–10), adolescence (10–18), young adulthood (18–45), middle adulthood (45–65), old adulthood (65+)
SES-DNAm measurement time	Categorical	Whether DNAm and SES were measured during childhood or adulthood	Both DNAm and SES measured in adulthood, both DNAm and SES measured in childhood, DNAm measured in adulthood and SES measured in childhood
Sex	Categorical	Sex distribution of the sample	Predominantly (>65%) female, predominantly male, mixed
Cell composition	Categorical	If cell composition is corrected for	Cell composition corrected, cell composition not corrected
Chip version	Categorical	Chip used to retrieve DNAm	450k, EPIC, 450k and EPIC, Not Available (NA)
Covariates	Categorical	If covariates were included in the model	≤5 covariates versus >5 covariates included Smoking included/not included as covariate BMI included/not included as covariate

## Results

### Descriptive results

**Fig. 1:**
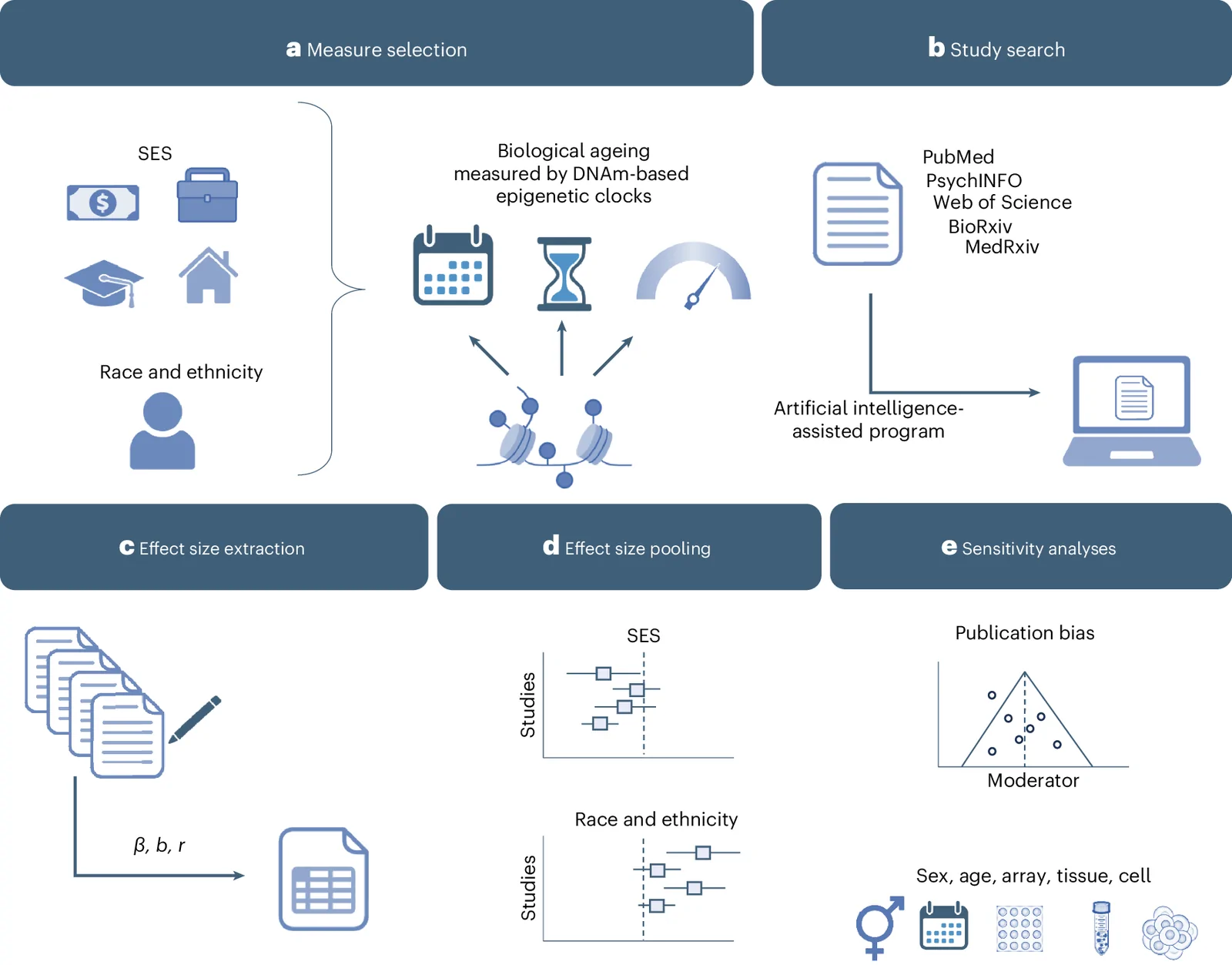
Methodological workflow of the present meta-analysis. **a**, Measures of interest, including SES, race and ethnicity and epigenetic-clock measures of biological ageing. **b**, The study search process across PubMed, PsycINFO, Web of Science, bioRxiv and medRxiv databases. The results were screened at the title and abstract level using an artificial intelligence-assisted programme and then screened at the full-text level by two researchers. **c**, Effect sizes, along with information on the study and cohort characteristics, were extracted and harmonized from the selected articles by three authors. **d**, Effect sizes were converted to a common metric and pooled. The associations of SES and self-reported race and ethnicity with epigenetic clock-measured biological ageing were pooled and meta-analysed. **e**, Moderator analyses for pre-registered technical and theoretical moderators as well as sensitivity analyses, including probing evidence for publication bias. Created in BioRender; Willems, Y. https://biorender.com/o37a1rg (2026).

**Fig. 2:**
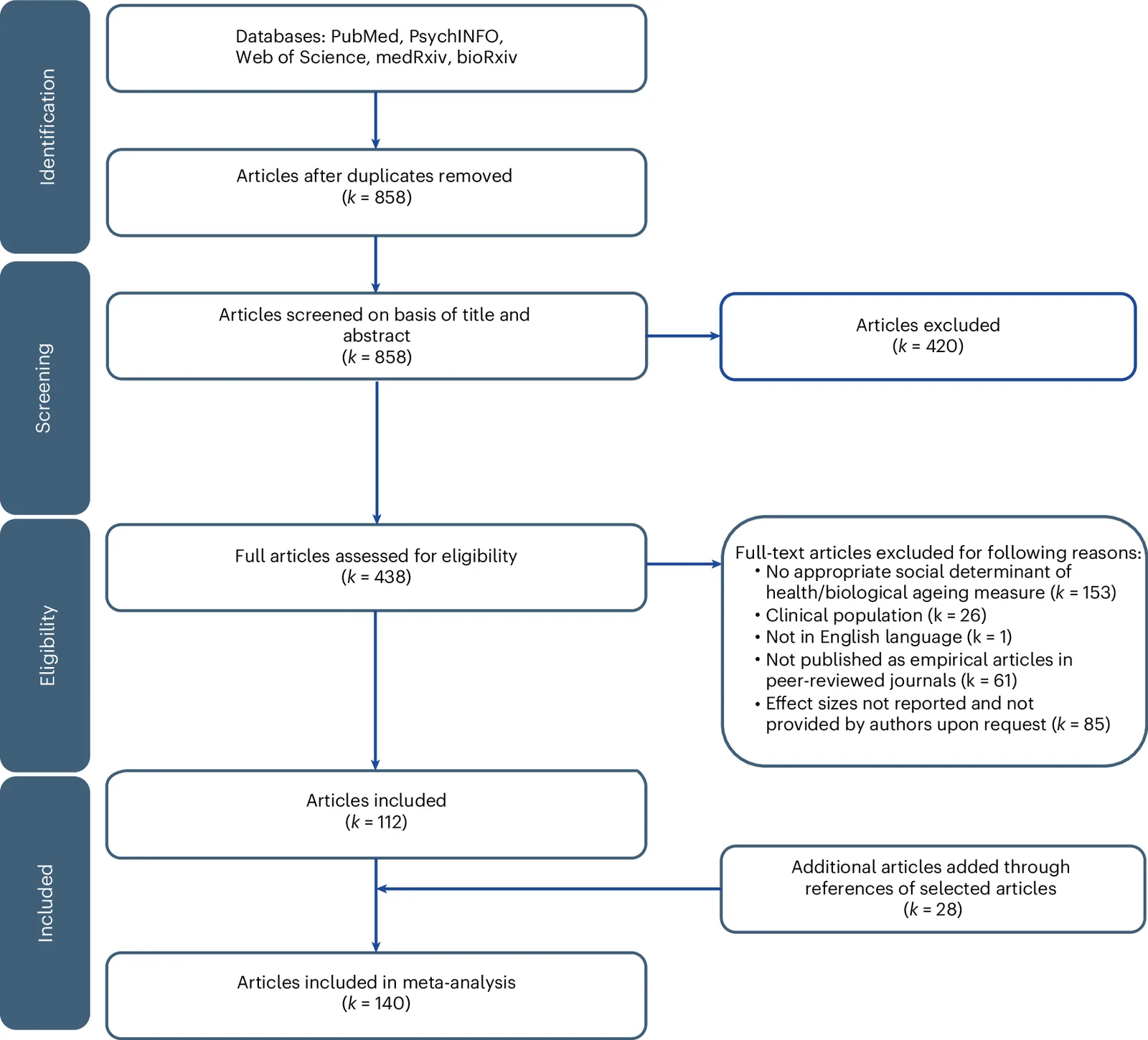
PRISMA flow diagram for the meta-analysis. The search was conducted from 2013 to February 2024.

Figure 1 presents the workflow of our analysis. Our final meta-analytic dataset consisted of 140 studies, including *N* = 65,919 unique participants and a total of 1,065 effect sizes derived from 79 unique cohorts drawn from 23 different countries (Supplementary Fig. 1; see Fig. 2 for PRISMA flow chart; see Table 3 for inclusion criteria). Of the 1,065 extracted effect sizes, 1,017 were included in the main analyses examining associations between epigenetic clock-based biological ageing and social determinants of health: 908 for SES and 109 for race and ethnicity (68 Black–white and 41 Latinx–white comparisons). The remaining 48 effect sizes were analysed separately, including 34 assessing changes in biological ageing and/or SES over time and 14 using biweight midcorrelation (bicor) estimates. The majority of cohorts (52%) were from the USA followed by the UK (9%). More than half of the cohorts (57%) were based on predominantly (>60%) white samples, and the majority (77%) had a mixed distribution of males and females. Sample sizes across all cohorts ranged from *n* = 33 to *n* = 16,245. The mean age was 49.9 years, ranging from 0–86 years.

**Table 3:** Inclusion and exclusion criteria for this meta-analysis

Inclusion criteria	Exclusion criteria
- Written in English - Publishing date after 2013 (first publication of Epigenetic clock-measured biological ageing) - Reporting effect size(s) including the association between at least one social determinant of health and one DNAm measure of biological ageing	- Reviews, dissertations, book chapters, conference abstracts - Studies on clinical populations - Age acceleration not reported for first- and second- generation measures - Effect sizes not reported and not provided by authors upon request

### Associations of SES with biological ageing by epigenetic clock generation and measure

We first examined whether epigenetic clock-measured biological ageing was associated with SES, considering the influence of clock generation and clock measure. For this analysis, we conducted a statistical test of effect-measure-modification (moderation) of SES–clock associations by clock generation or measure. Effect sizes included in this analysis were derived from 23 countries, including individuals from birth to old age and various measures of SES (Table 1).

**Fig. 3:**
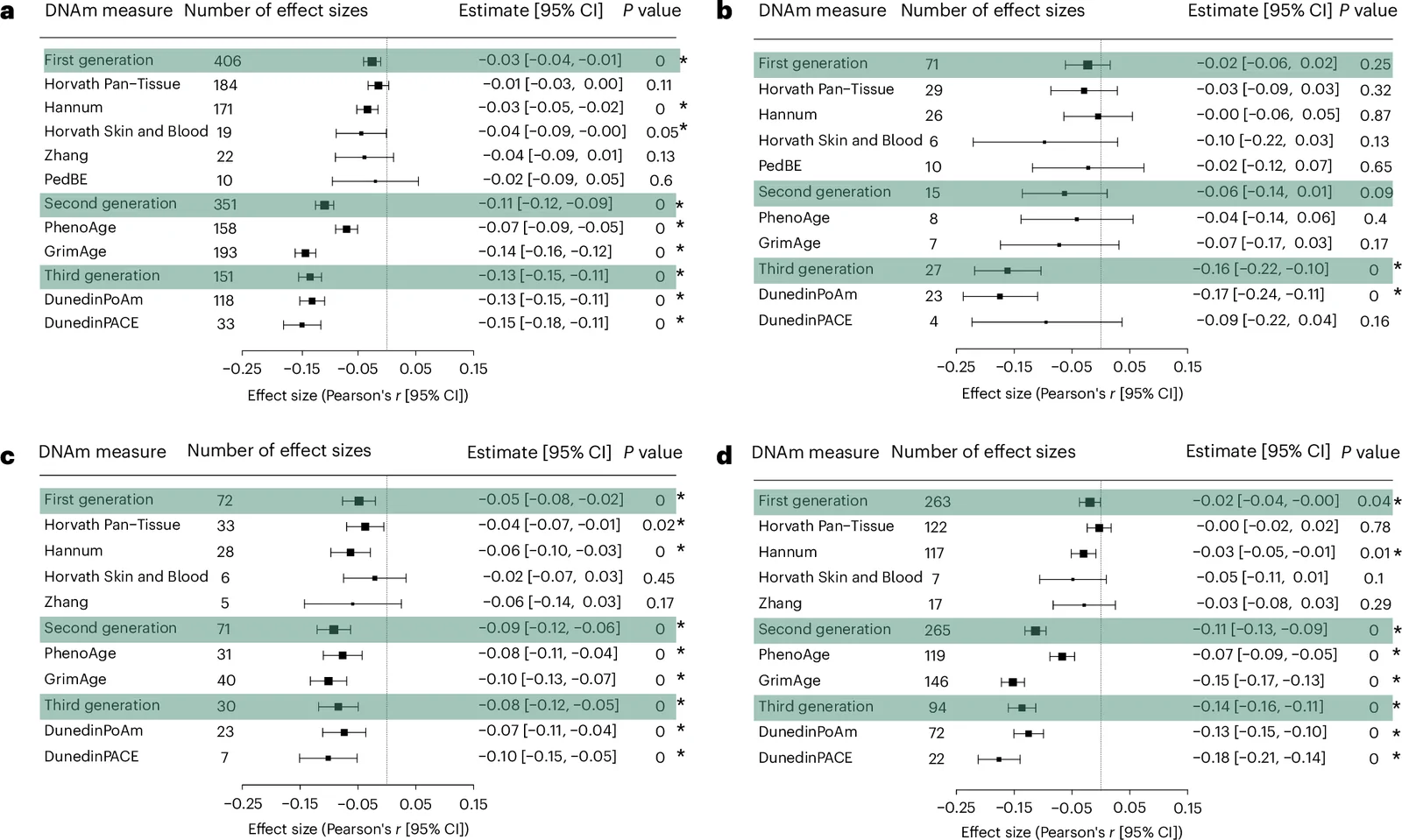
Meta-analytic association between SES and epigenetic clock-measured biological ageing. **a**−**d**, Forest plots depict meta-analytic associations of SES and epigenetic clock-measured biological ageing (**a**), childhood SES and epigenetic clock-measured biological ageing measured in childhood (**b**), childhood SES and epigenetic clock-measured biological ageing in adulthood (**c**) and adulthood SES and epigenetic clock-measured biological ageing measured in adulthood (**d**). Green rows indicate the meta-analysed effect size estimates for first-, second- and third-generation clocks, followed by individual epigenetic clocks within each category. Two-sided tests were conducted using Fisher-*Z* transformation and later transformed to Pearson’s *r* to ease interpretation. Effect size estimates in Pearson’s *r* are bounded by 95% CIs. Asterisks denote significant difference from zero at *P* < 0.05. The size of the boxes in the middle of each panel represents the precision of the effect-size estimate (that is, the smaller the CI is, the more precise the estimate is).

We found that individuals of lower SES tended to exhibit accelerated epigenetic clock-measured biological ageing compared with individuals of higher SES. The magnitude of this association between SES and biological ageing was moderated by clock generation (*F*(2) = 178.10, *P* < 0.001) and measure (*F*(8) = 244.88, *P* < 0.001; Supplementary Table 1a). It was largest for third-generation measures (*r* = −0.13, 95% confidence interval (CI) [−0.15, −0.11], *P* < 0.001), followed by second-generation measures (*r* = −0.11, 95% CI [−0.12, −0.09], *P* < 0.001), both of which were larger than first-generation measures (*r* = −0.03, 95% CI [−0.04, −0.01], *P* < 0.001); pairwise comparisons *z* = −11.05, *P* < 0.001 for second- versus first-generation, *z* = −11.26, *P* < 0.001 for third- versus first-generation, *z* = −2.67, *P* < 0.01 for third- versus second-generation; Fig. 3a; see Supplementary Table 1a for pairwise comparisons across all clocks). Among the individual clock measures, the SES–clock associations were most pronounced for GrimAge acceleration, DunedinPoAm and DunedinPACE compared with all other DNAm clocks (Fig. 3a and Supplementary Table 1a). These three measures indicated accelerated ageing in lower relative to higher SES individuals (*r* values from −0.13 to −0.15).

We tested for publication bias in SES–clock associations using funnel plots, Egger’s test and trim-and-fill analyses. No evidence of bias was found: the plots were symmetric (Supplementary Fig. 2), Egger’s tests were non-significant, and trim-and-fill minimally affected magnitude of effect sizes (Supplementary Table 1b,c). Sensitivity analyses excluding data from the Health and Retirement Study (HRS), meta-analyses, preprints, effect sizes with small sample sizes (*n* < 100) and leave-one-cohort- out tests produced negligible changes in effect sizes (*r* = 0–0.02; Supplementary Figs. 3–6 and 17–19 and Supplementary Table 1d–g), confirming the robustness of the results. All estimates in Pearson’s *r* are illustrated in Supplementary Fig. 20. Corrections for multiple comparisons did not alter differences between clock generations or the significance of GrimAge, DunedinPoAm and DunedinPACE compared with other clocks.

#### Measurement timing effects of SES and epigenetic clock-measured biological ageing

We then probed when associations of SES with epigenetic clock-measured biological ageing were apparent. Because of the hypothesized importance of childhood environments on lifelong ageing trajectories^30,31^, our main focus was on three combinations of measurement timing: (1) children’s DNAm with childhood SES, (2) adults’ DNAm with childhood SES and (3) adults’ DNAm with adulthood SES.

We note that DNAm collected in adulthood (87.6% of effect sizes) is more common than DNAm in childhood (12.4%). None of the studies had DNAm from the same people collected repeatedly in both childhood and adulthood. Moreover, most cohort studies included either children or adults, but not both. Thus, these analyses can be understood as probing for age and generational cohort differences alongside other study-specific effects (for example, child studies are substantially more likely to have saliva rather than blood DNAm).

#### Children’s DNAm with childhood SES

We wanted to know whether children living in families of lower SES versus higher SES differed in biological ageing, as measured by childhood DNAm. Of the 113 effect sizes, most were from the USA (60 effect sizes) and the UK (34 effect sizes) studies, with a mean age of 10.07 years, ranging from 0 to 18 years; 37% of the effect sizes were estimated in samples of saliva DNAm and 53% in samples of venous blood.

In an analysis restricted to child DNAm, we found that children living in lower SES families tended to have a faster pace of ageing than their more affluent peers. The magnitude of this association between SES and biological ageing was moderated by clock generation (*F*(2) = 18.91, *P* < 0.001) and clock measure (*F*(7) = 22.01, *P* < 0.001; Supplementary Table 2a). We found that only third-generation clocks were significantly associated with SES (Fig. 3b; *r* = −0.16, 95% CI [−0.22, −0.1], *P* < 0.001; pairwise comparisons *z* = −1.01, *p* = 0.31 for second- versus first-generation, *z* = −4.33, *P* < 0.001 for third- versus first-generation and *z* = −2.35, *P* < 0.05 for third- versus second-generation; see Supplementary Table 2a for pairwise comparisons across clocks). Among individual clock measures, only DunedinPoAm (*r* = −0.18, 95% CI [−0.24, −0.11], *P* < 0.001) was significantly associated with SES. There were insufficient effect sizes to compare DunedinPoAm to DunedinPACE ( < 4 ES).

#### Adult’s DNAm with childhood SES

We next examined whether adults who—as children—grew up in families of lower SES versus higher SES differed in biological ageing as measured in their adult DNAm. Most of the studies had retrospective self-reports of childhood SES. Of the 173 effect sizes across 6 countries, most were from US studies (145), with a mean age of 56.7 years, ranging from 20 to 79, and venous blood DNAm was the most common tissue, which was measured in 84% of effect sizes.

In an analysis restricted to childhood SES and adulthood DNAm, we found that adults who grew up in lower SES families tended to have an accelerated biological age and faster pace of ageing than their more affluent peers. The magnitude of the association between SES and biological ageing was moderated by clock generation (*F*(2) = 14.74, *P* < 0.001) and clock measure (*F*(7) = 21.71, *P* < 0.001, Supplementary Table 2b). It was strongest for second-generation clocks (*r* = −0.09, 95% CI [−0.12, −0.06], *P* < 0.001) and third-generation clocks (*r* = −0.08, 95% CI [−0.12, −0.05], *P* < 0.001) and weakest for first-generation clocks (*r* = −0.05, 95% CI [−0.08, −0.02], *P* < 0.001; Fig. 3c and Supplementary Table 2b); pairwise comparisons *z* = −3.72, *P* < 0.001 for second- versus first-generation, *z* = −2.41, *P* < 0.05 for third- versus first-generation, *z* = 0.54, *P* = 0.59 for third- versus second-generation. Among the individual clock measures, the SES–clock associations were most pronounced for GrimAge acceleration, PhenoAge acceleration, DunedinPoAm and DunedinPACE (*r* values range from −0.07 to −0.11; see Fig. 3c and Supplementary Table 2b for pairwise comparisons also across other clocks).

#### Adult’s DNAm with adulthood SES

Next, we tested whether adults currently of lower SES versus higher SES differed in biological ageing, as measured in their adult DNAm. Most of the 622 effect sizes came from the USA (427 effect sizes), followed by the UK (42 effect sizes). The mean age was 55.75, ranging from 19.3 to 85.8. The most commonly used tissue was venous blood, contributing 551 effect sizes (89%).

In an analysis restricted to adulthood SES and adulthood DNAm, we found that adults currently of lower SES tended to have an accelerated biological age and faster pace of ageing than those of higher SES. The magnitude of the association between SES and biological ageing was moderated by clock generation (*F*(2) = 168.98, *P* < 0.001) and clock measure (*F*(7) = 275.02, *P* < 0.001; Supplementary Table 2c). It was most pronounced for third-generation clocks (*r* = −0.14, 95% CI [−0.16, −0.11], *P* < 0.001), followed by second-generation clocks (*r* = −0.11, 95% CI [−0.13, −0.09], *P* < 0.001), and weakest for first-generation measures (*r* = −0.02, 95% CI [−0.04, −0.00], *P* < 0.05; Fig. 3d and Supplementary Table 2c); pairwise comparisons *z* = −11.34, *P* < 0.001 for second- versus first-generation, *z* = −0.12, *P* < 0.001 for third- versus first-generation, *z* = −2.16, *P* < 0.05 for third- versus second-generation. Among individual clock measures, GrimAge, DunedinPoAm and DunedinPACE had the largest effect sizes (*r* values range from −0.13 to −0.18) compared with all other clocks (Fig. 3d; see Supplementary Table 2c for pairwise comparison across clocks).

#### Child and adult DNAm with SES

We next examined whether epigenetic clock associations with SES significantly differed by measurement timing. We restricted this analysis to GrimAge and DunedinPoAm. For both clocks, the magnitude of the SES association was moderated by measurement timing (DunedinPoAm (*F*(2) = 16,75, *P* < 0.001; GrimAge (*F*(2) = 14,92, *P* < 0.001; Supplementary Table 2d). The DunedinPoAm–SES association was significantly weaker when SES was measured in childhood and DNAm in adulthood compared with when DunedinPoAm–SES were both measured in childhood or both measured in adulthood. The DunedinPoAm–SES association was not statistically different when comparing childhood SES with child DNAm to adulthood SES with adult DNAm (see Supplementary Table 2d for pairwise comparisons).

The GrimAge–SES association was significantly weaker when SES was measured in childhood and DNAm in adulthood compared with when GrimAge–SES were both measured in adulthood (Supplementary Table 2d). Although the GrimAge–SES association was not significantly different from zero in child DNAm (*r* = –0.07, 95% CI [–0.02, 0.18], *P* = 0.17), the effect size did not significantly differ from the effect size of SES in adulthood with adults DNAm (*r* = –0.15, 95% CI [–0.17, –0.13], *P* < 0.001; Supplementary Table 2d). This may be attributable to the wide confidence interval and larger standard error in the child DNAm estimate, which are due to the limited number of effect sizes available for SES–GrimAge associations (only 7 effect sizes; Fig. 3b).

In the sensitivity analyses, we further delineated whether associations between childhood SES and DunedinPoAm differed when DNAm was measured in ‘childhood (5–10 years)’ or ‘adolescence (10–18 years)’. Moderation analyses showed no significant differences across these age bins (Supplementary Table 2e). Next, we probed whether the association between adulthood SES and GrimAge and DunedinPoAm differed when DNAm was measured in ‘young adulthood (ages 18–45)’, ‘middle adulthood (ages 45–65)’ and ‘old adulthood (age 65+)’. Moderation analyses showed no significant differences across these age bins (Supplementary Table 2e).

#### Intergenerational SES and repeated measures of biological ageing

Several studies examined clock associations with intergenerational SES, primarily by comparing first- and second-generation clock measures in individuals with stably low versus stably high SES across their lives. We found that when individuals grew up in low SES and had low SES in adulthood, they had accelerated biological ageing when compared with those who grew up in high SES and had high SES in adulthood, although this conclusion should be interpreted with caution considering that the number of effect sizes per category (only 2 effect sizes for PhenoAge and 2 for GrimAge; Supplementary Fig. 7) was lower than our threshold of 5, which limits statistical power.

Moreover, a few effect sizes explored the association between SES and repeated measures of biological ageing within individuals, or between repeated measures of both SES and biological ageing within individuals. Meta-analysing these few associations showed no significant link; thus, more studies are needed to better quantify these associations (Supplementary Fig. 8). Some studies investigated intergenerational mobility^24,32^; however, these did not yield sufficient effect sizes to synthesize in a meta-analysis.

### Differences between race and ethnic groups in biological ageing

**Fig. 4:**
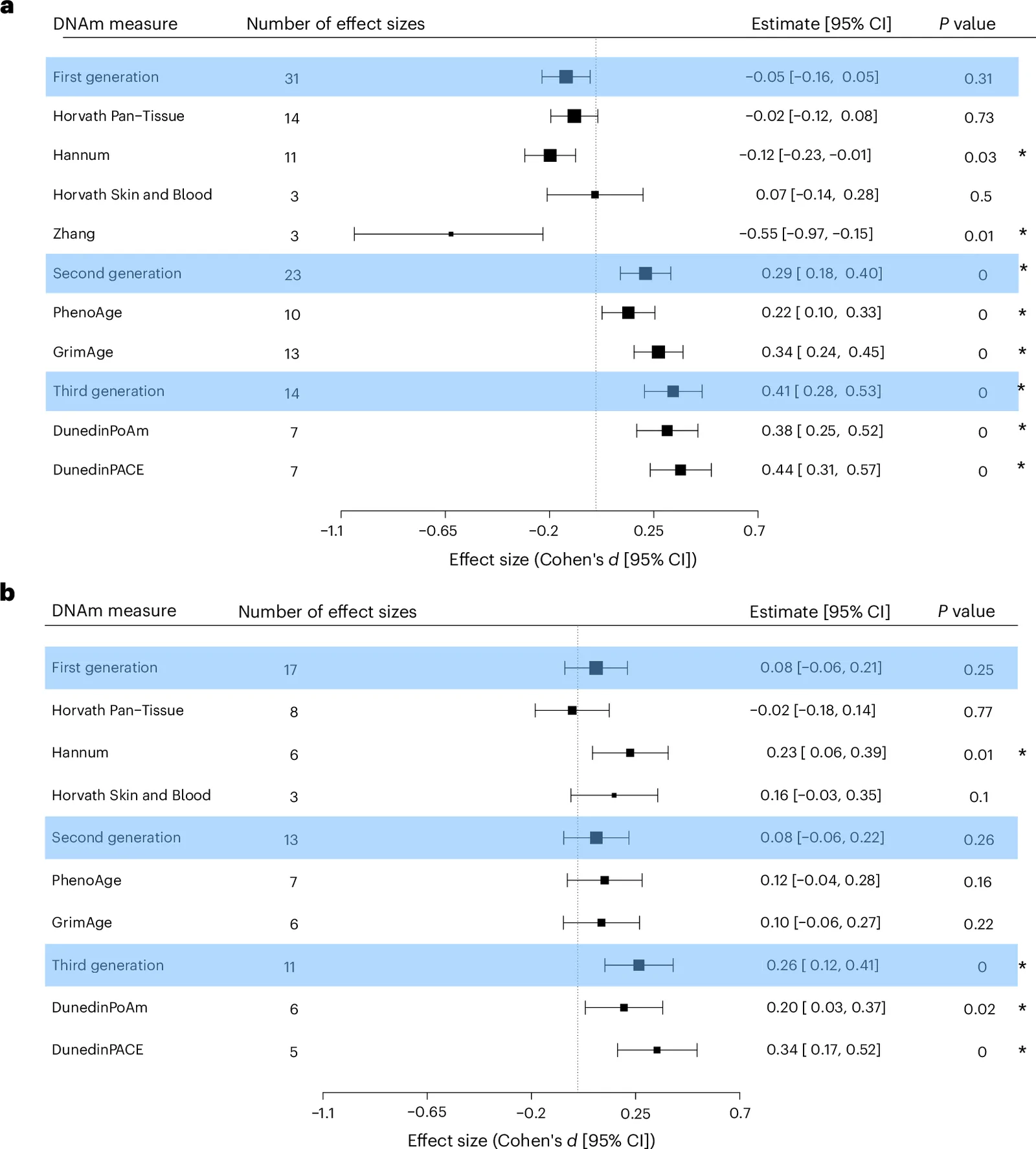
Meta-analytic effect sizes describing the differences between self-identified race and ethnicity in epigenetic clock-measured biological ageing. **a**,**b**, Forest plots depict meta-analytic associations of Black- versus white-identifying individuals (**a**) and Latinx- versus white-identifying individuals (**b**). Green rows indicate the meta-analysed effect size estimates for first-, second- and third-generation clocks, followed by individual clock measures within each category. Two-sided tests were conducted, and effect size estimates and 95% CIs are reported in Cohen’s *d*. Effect sizes report differences for self-identified racial and ethnic groups in reference to white. Asterisks denote significant difference from 0 at *P* < 0.05. The size of the boxes in the middle of each panel represents the precision of the effect-size estimate (that is, the smaller the CI is, the more precise the estimate is).

Next, we examined whether epigenetic clock-measured biological ageing differed between race and ethnic groups. All effect sizes included in this analysis came from studies of US-based cohorts. In our analysis, we found that Black individuals tended to exhibit accelerated biological ageing when compared with white individuals. The magnitude of this difference between racial groups in epigenetic clock-measured biological ageing was moderated by clock generation (*F*(2) = 88.40, *P* < 0.001) and measure (*F*(7) = 121.41, *P* < 0.001; Supplementary Table 3a). The effect was largest for third-generation measures (*d* = 0.41, 95% CI = [0.28, 0.53], *P* < 0.001) compared with second-generation measures (*d* = 0.29, 95% CI = [0.18, 0.40], *P* < 0.001), both of which were larger than first-generation measures (*d* = −0.05, 95% CI = [−0.16, 0.05], *P* = 0.31); pairwise comparisons *z* = 7.41, *P* < 0.001 for second- versus first-generation, *z* = 8.43, *P* < 0.001 for third- versus. first-generation, *z* = 2.17, *P* < 0.05 for third- versus second-generation (Fig. 4a and Supplementary Table 3a).Among the individual clock measures, the difference between Black- and white individuals was most pronounced in GrimAge acceleration, DunedinPoAm and DunedinPACE (range of *d* values 0.34 to 0.44) compared with all other DNAm measures (Fig. 4a; see Supplementary Table 3a for pairwise comparison across all clocks). These three measures indicated accelerated ageing in Black relative to white individuals. In contrast, the Hannum (*d* = −0.12 [−0.23, −0.01]) and Zhang (*d* = −0.55 [−0.97, −0.15]) clocks indicated biological age deceleration in Black relative to white individuals. While slight deceleration was observed for the Hannum and Zhang clocks, which primarily index chronological age, the second-generation clocks that capture morbidity- and mortality-related biological processes indicated faster ageing among Black compared with white participants, consistent with established health disparities^4,33,34^.

Second, we examined whether epigenetic clock-measured biological ageing differed between Latinx and white individuals, given social disparities in health between these groups^35^. We found that Latinx individuals tended to exhibit accelerated biological ageing when compared with white individuals. Again, the magnitude of this difference between ethnic groups and DNAm measures was moderated by clock generation (*F*(2) = 14.91, *P* ≤ 0.001) and measure (*F*(6) = 71.18, *P* < 0.001; Supplementary Table 3b). The difference was largest for third-generation measures (*d* = 0.26, 95% CI [0.12, 0.41], *P* < 0.001) compared with second-generation and first-generation measures, both of which were not significantly different from zero; pairwise comparison *z* = 0.02, *P* = 0.98 for second- versus first-generation, *z* = 3.45, *P* < 0.001 for third- versus first-generation, *z* = 3.49, *P* < 0.001 for third- versus second-generation (Fig. 4b and Supplementary Table 3b). Among the individual clock measures, the difference between Latinx and white groups was most pronounced in DunedinPACE (*d* = 0.34, 95% CI [0.17, 0.52], *P* < 0.001) compared with all other DNAm measures (Fig. 4b, see Supplementary Table 3b for pairwise comparison across all clocks). We could not investigate the heterogeneity in race and ethnicity associations with epigenetic clocks by the timing of DNAm measurement, as there were relatively few studies involving racially marginalized children. We then examined potential publication bias in associations between race and ethnicity and epigenetic clock measures using funnel plots, Egger’s regression and trim-and-fill analyses. Funnel plot’s and Egger’s test showed asymmetry for the Hannum and GrimAge clocks in Black–white comparisons (Supplementary Fig. 9 and Supplementary Table 3c); however, trim-and-fill corrections produced minimal changes in effect size estimates (Supplementary Table 3d). For Latinx–White comparisons, Horvath Pan-Tissue, GrimAge and DunedinPACE indicated possible bias (Supplementary Fig. 10 and Supplementary Table 3e), and trim-and-fill results showed differences in magnitude of the effect sizes (Supplementary Table 3f). However, the results were uncertain due to the small number of studies and high heterogeneity (≥80%). Excluding preprints or small-sample effect sizes minimally altered results by 0–0.02 in Cohen’s *d* (Supplementary Figs. 11 and 12 and Supplementary Table 3g–j). Removing HRS showed stronger alterations Black–white effects by ~0.03–0.21 and Latinx–white by ~0.04–0.16 (Supplementary Fig. 13 and Supplementary Table 3k,l), as HRS contributed most of the race and ethnicity effect sizes. Leave-one-cohort-out tests for SES–clock links changed *d* by 0–0.02, occasionally 0.04 (Supplementary Figs. 18 and 19).

### Factors potentially modifying the magnitude of the association between SES–epigenetic clocks and race and ethnicity–epigenetic clock

#### Sex

We explored whether the strength of SES–clock associations varied depending on sex. Few studies included SES–clock associations for females and males separately. In line with earlier meta-analyses^36,37^, we compared associations of sex ratio balanced and sex ratio imbalanced (>65% females or males). We found moderating effects of sex for Hannum and PhenoAge, where associations were more pronounced when derived from predominantly female samples compared with sex ratio balanced samples (Supplementary Table 4a; sex differences for Hannum did not survive correction for multiple comparison). We did not find moderating effects of sex for other clocks (Supplementary Table 4b). We did not probe sex moderation for race and ethnicity, nor specifically in childhood samples, because there were too few studies with sex-imbalanced cohorts.

#### Technical factors

We next examined whether the strength of SES–clock associations varied depending on technical factors (for example, tissue type, array and cell correction). However, our analysis was substantially hindered by inconsistent or missing reporting of which technical factors were included as covariates. Thus, our findings should be considered preliminary and require further examination in future studies.

First, we examined whether SES–clock associations were moderated by tissue type (for example, blood or saliva), and no differences were found (Supplementary Table 4b). Because saliva samples were primarily available in child studies, we conducted an exploratory analysis to examine whether tissue type moderated the association between childhood SES and children’s DNAm. Only the Horvath Pan-Tissue, Hannum and DunedinPoAm clocks had sufficient effect sizes across both tissue types to allow moderation testing, and none showed significant differences between blood and saliva (Supplementary Table 4c).

Next, we explored whether associations varied depending on the array type (EPIC, 450k). We pre-registered analyses comparing EPIC v2 and EPIC v1; however, none of the studies in our meta-analysis used the EPIC v2 array because it was only recently released. We found no differences in SES–clock associations between arrays for clock generation or individual clocks (Supplementary Fig. 14 and Supplementary Table 4b, See S4D-E for estimates across arrays). We then probed whether SES–clock associations differed by adjustment for cell composition in different tissue types and found no differences (Supplementary Table 4f). For race and ethnicity and epigenetic clocks there was a limited number of covariates that had enough effect sizes per clock, and none showed significant moderating effects (only Horvath Pan-Tissue, Hannum and GrimAge had the required >5 effect sizes for moderator analyses to test cell composition, and only Horvath Pan-Tissue was used to test array type; Supplementary Table 4g).

#### Covariates

Last, we examined whether the number and type of covariates (smoking, BMI) moderated associations of SES with epigenetic clocks. Across studies, covariate counts ranged from 0 to 37; 643 effect sizes included >5 covariates, 420 adjusted for smoking, and 348 adjusted for BMI. Moderator analyses indicated that the SES–DunedinPoAm association was stronger in models with fewer covariates (≤5: *r* = −0.17) than in those with more (>5: *r* = −0.11), whereas PhenoAge, GrimAge and DunedinPACE were not significantly affected by covariate count (Supplementary Table 4h,i). Adjusting for smoking attenuated SES–GrimAge (*r* = −0.16 to −0.07) and SES–DunedinPoAm (*r* = −0.17 to −0.06) associations, but did not effect other SES–clock associations (Supplementary Table 4h,i). Similarly, adjusting for BMI attenuated SES–GrimAge (*r* = −0.15 to −0.07) and SES–DunedinPoAm (*r* = −0.16 to −0.06) associations, but did not affect other SES–clock associations (Supplementary Table 4h,i). Examining the effects of covariates on the association between race and ethnicity and epigenetic clocks was limited by the number effect sizes (only Horvath Pan-Tissue, Hannum, PhenoAge and Grimage had the minimum required five effect sizes per category to test effects of smoking as covariate, and only Horvath Pan-Tissue and Hannum were used to test for the effect of BMI as a covariate). We did find a reduced effect size for the Black–white aasociation on clocks in Hannum (Cohen’s *d* = −0.05 to 0.02) when smoking was included as covariate, with no effects on other associations (Supplementary Table 4j,k). All findings held after correction for multiple comparisons.

#### Systematic review

To ensure that our findings reflect the most current evidence, we conducted an updated systematic search covering studies published from February 2024 to September 2025. Following our pre-registered protocol, we identified 280 records. After title and abstract screening, we reviewed 67 full-text articles; resulting in 33 studies after excluding studies based on inclusion criteria (composed of 21 adult samples and 14 adolescent/child samples; 4 drew on cohorts included in our previous synthesis (for example, HRS, Framingham Heart Study)).

Overall, the results aligned with our meta-analysis (Supplementary Table 5). First-generation clocks showed little to no association with SES or race and ethnicity, whereas later-generation clocks (GrimAge, PhenoAge and DunedinPACE) yielded more consistent associations. Lower versus higher SES and Black versus white racial identity were linked to faster ageing. Effect sizes for second- and third-generation clocks in the new studies fell within our meta-analytic range (SES *r* = –0.12 to –0.20; race *d* = 0.12–0.25). Studies examining specific SES indicators (for example, spousal or grandparent education) produced more variable findings. PhenoAge had the least consistent results, with several null associations for SES or race, consistent with our observation that it appears less sensitive than GrimAge or DunedinPACE. Taken together, the updated search supports the robustness of our meta-analytic conclusions.

## Discussion

We conducted a pre-registered, multi-level meta-analysis synthesizing 1,065 effect sizes across 140 studies on the associations of SES or race and ethnicity with three generations of epigenetic clock measures of biological ageing. Previous systematic reviews and meta-analyses examining socioeconomic and racial and ethnic differences in epigenetic ageing have been limited in scope^10,38–40^, whereas our study quantitatively synthesizes evidence across first-, second- and third-generation clocks, integrating findings from both child and adult samples to provide a comprehensive view of how social determinants relate to biological ageing across the lifespan. The included studies comprised *N* = 65,919 people from 23 countries aged 0 to 86 years. There were four key findings.

First, SES is associated with biological ageing with lower SES being associated with older biological age and faster biological ageing. When considered by generation, effect sizes were most consistent for the third generation of epigenetic clocks, which were developed by summarizing rates of change across multiple organ systems. These were followed by the second generation of epigenetic clocks, which were developed by modelling mortality risk. In contrast, the first generation of epigenetic clocks, which were developed by modelling chronological age, demonstrated small or non-significant associations with SES. When considered at the level of individual epigenetic clocks, third-generation DunedinPoAm and DunedinPACE and second-generation GrimAge exhibited the strongest associations with SES. Strikingly, we did not find evidence of publication bias in SES–clock associations. Since our initial search, a growing number of studies have documented associations between SES, racial and ethnic disparities and epigenetic clocks, which we synthesized in our systematic review. Taken together, the updated search supports the robustness of our meta-analytic conclusions.

Second, SES associations with third-generation clocks were similar in magnitude when DNAm was measured in childhood and adulthood, whereas second-generation clocks showed weaker associations in children. Fewer paediatric studies included second-generation clocks, and although effect sizes differed by approximately twofold, the differences were not significant. Childhood SES showed comparable associations with second- and third-generation clocks measured in adult blood. Collectively, these findings suggest that third-generation clocks such as DunedinPACE, alongside second-generation clocks like GrimAge, are promising tools for tracing environmental effects on ageing early in life. While ageing probably begins at conception^29,41^, its relation to development remains unclear. Accelerated ageing may divert resources from growth, or developmental activity itself may induce damage. Molecular epidemiology is now linking ageing biomarkers to developmental processes, including pubertal timing^23,42^. Because clocks were trained in adults with different blood composition and no active developmental programmes, paediatric estimates may be less accurate and reflect both ageing and development, which is why we should interpret them with caution.

Third, while deceleration was observed for the Hannum and Zhang clocks, which primarily index chronological age, the second-generation clocks that capture morbidity- and mortality-related biological processes indicated faster ageing among Black versus white participants, consistent with established health disparities^4,33–35^. Group differences were larger for Black–white comparisons than for Latinx–white comparisons. Similar to our findings for SES, those racial and ethnic disparities were most pronounced for third-generation epigenetic clocks, followed by second-generation clocks. Again, DunedinPoAm, DunedinPACE and GrimAge showed the starkest social differences in epigenetic clock-measured ageing. We did find evidence for publication bias for some clocks for race and ethnicity results; however, caution is warranted in the interpretation given the high level of heterogeneity. Racism intersects with socioeconomic disadvantage and other health risks, creating complex challenges^43–45^. Effect sizes were larger for racial and ethnic than SES disparities. However, studies relying on self-reported race and ethnicity cannot capture structural or individual-level racism (for example, segregation, discrimination). Limited data on racially marginalized children also prevented testing whether racial disparities vary by DNAm measurement timing.

Fourth, we found little evidence that sex, array, tissue type or cell-count correction moderated SES or racialized differences in epigenetic ageing. Still, whether these factors influence SES–clock associations warrants further study. Future work should directly compare associations between females and males, as our meta-analysis could only contrast studies that were mostly male, mostly female, or sex-balanced. Among adults, men typically show older biological age and faster ageing than women, whereas in adolescence, girls show older biological age and faster ageing, consistent with earlier pubertal timing^18,42,46^. Sex-specific racial and ethnic disparities remain underexplored, although our results suggest that social disparities in GrimAge and DunedinPACE are similar across sexes. Few studies have tested whether SES–clock associations differ by tissue type in children, but previous work shows moderate-to-high correspondence between blood and saliva after cell adjustment^47^. Although some signal may be lost in saliva, SES associations with GrimAge, DunedinPoAm and DunedinPACE appear robust to such technical variation. Measuring DNAm in both tissues will clarify how cellular context shapes clock associations in childhood.

Fifth, future research should clearly justify covariate selection and, where feasible, report effect sizes with and without key adjustments, as our analysis was constrained by limited reporting across studies. Although adjusting for smoking or BMI appears to attenuate some SES–clock associations, these findings should be interpreted cautiously because these covariates were typically included alongside numerous others, potentially obscuring their specific contributions.

These findings should be interpreted within the framework of several limitations. There are epigenetic clocks that were not included in our analysis, including new clocks for which studies of SES have not yet been conducted^48,49^. Our analyses of sex differences and technical modifiers affecting SES–clock associations lacked complete information. Some studies failed to report complete information on technical details, with this variability in reporting practices and methodological differences potentially effecting pooled estimates, and the number of studies conducted in non-blood tissues remains small. Finally, analysis of race and ethnic variation in epigenetic clocks may be confounded by ancestry-linked genetic artefacts^50,51^. However, there is little evidence so far to support this type of bias in the case of the second- and third-generation clocks; studies find consistent patterns of clock associations with health within different race and ethnic groups^24,52–56^. An urgent priority is to develop and validate biological ageing algorithms that are inclusive and globally representative. This will require expanding data collection beyond predominantly Western, high-income cohorts to encompass participants from varied socioeconomic and geographic contexts, ensuring that epigenetic measures accurately reflect ageing processes worldwide.

There is now abundant correlational evidence linking a range of socially stratified exposures with epigenetic clock measures of biological ageing (for example, environmental toxicants, access to healthcare and stress)^6,10,57–60^. Quasi-experimental studies further provide causal evidence that early-life conditions—poverty, in utero undernutrition from famine and lower educational attainment—accelerate later-life epigenetic ageing^32,61,62^. However, few studies have included repeated measures of DNAm to examine how changes in environmental conditions correspond to changes in biological ageing. Ongoing trials are probing epigenetic clock sensitivity to randomized interventions, including behavioural programmes^63,64^ and cash transfers. Our results can guide the choice of outcome measures in future studies.

## Methods

The Preferred Reporting Items for Systematic Reviews and Meta-Analyses (PRISMA) Checklist^65^ was used to structure, conduct and report this meta-analysis. Deviations from preregistration are described in Supplementary Table 6 (https://osf.io/u5z82). The full meta-analytic dataset and analytic scripts are available in Supplementary Tables 7 and 8 and on our GitHub, https://github.com/Biosocial/Meta-SDoH-EpigeneticClocks.

### Search terms

Table 1 presents an overview of the variables of interest for epigenetic clock-measured biological ageing and social determinants of health, which we restrict to measures of SES and racialization. These variables were used to produce a search string for a systematic literature search (Supplementary Table 9).

### Search strategy and information sources

For the literature search, the following databases were used: PubMed, PsycINFO, Web of Science, medRxiv and bioRxiv. Search for medRxiv and bioRxiv was performed using R’s medrxivr package. Literature search was completed at one time point, and not repeated thereafter. The search string was used to scan titles and abstracts, with specific search terms listed in Supplementary Table 6.

We included studies published or pre-printed between 2013 (when the first epigenetic clock, the Horvath Pan-Tissue clock, was published) and 14 February 2024 (when the meta-analytic search was conducted).

### Eligibility criteria

Study inclusion and exclusion criteria are presented in Table 3. Supplementary Fig. 15 shows a heatmap of effect sizes across socioeconomic and race and ethnicity measures with the darker shades corresponding to larger numbers of effect size estimates included in the meta-analysis.

### Selection procedure

Zotero was used to manage and organize papers. Authors A.D.R. and A.B. independently screened the obtained articles based on their titles and abstracts using ASReview^66^, an open-source artificial intelligence-assisted programme for systematic reviews. Then, selected and rejected articles were compared and where differences arose, they were resolved through discussion. This step led to the exclusion of 420 articles. The two authors then independently performed full-text screening of the remaining 438 articles. After discussion over disagreements, a further 326 articles were excluded (Fig. 2).

During the full-text screening process, 114 articles that included relevant SES, race, ethnicity and biological ageing measures but did not report the effect sizes of their associations were identified. Emails were sent to the corresponding authors to request the effect sizes. The authors were given three weeks to provide the data. A reminder email was sent two weeks after the initial email as a follow-up. Out of these 114 articles, we did not receive a response for 71 (62.3%) articles. Out of the authors that responded, data were received for 19 (16.7%) articles. Ten articles we did not receive data for still included relevant results; thus, they were included in this meta-analysis.

The references of the articles selected for inclusion were screened by A.D.R. to find relevant articles that were not identified by the initial literature search. Among the 41 articles identified in this step, 19 included relevant SES and biological ageing measures but did not report effect sizes. Requests for data were sent via email as previously described. We did not receive any response for 9 (56.3%) articles and data were received for 6 (31.6%) articles. This step led to the inclusion of 28 articles, resulting in a final meta-analytic sample of 140 articles.

### Coding of studies

A coding scheme was developed based on meta-analysis guidelines^67^, including study descriptives (for example, authors, title and year of publication) and study characteristics (for example, sample size, country, participants, DNAm measure(s) of biological ageing, SES measure, race and effect sizes). Potential moderator variables of interest (see ‘Moderator analyses’ section) were listed in the coding scheme.

While epigenetic-clock measures of biological ageing were consistently reported as continuous variables, this was not always the case for SES measures. Some studies reported them as continuous variables (for example, years of education), while others reported them as categorical variables (for example, highest educational attainment). Effect sizes for associations between two continuous variables were taken as reported. For studies reporting the association between biological ageing and categorical variables, only the effect size denoting the difference between the lowest and highest categories was extracted. To ensure consistency, SES variables were adjusted when necessary, so that higher values indicated higher SES.

Twenty percent of the articles were randomly selected to be coded by three authors, A.D.R., M.A. and A.B. Two methods were used to evaluate inter-rater reliability. For continuous variables, intraclass correlation was calculated and it ranged from 0.85 to 1. For categorical variables, Cohen’s *κ* was measured, with values between 0.57 and 1. Where disagreements arose, they were resolved by in-depth reading and discussion with author Y.E.W. After this inter-rater step, the remaining studies were divided and coded separately by the raters.

The preferred effect size metric was Pearson’s correlation coefficient *r*. However, if Pearson’s *r* was not reported in a given study, the following effect size metrics were extracted: Cohen’s *d*, standardized regression coefficient *β*, non-standardized regression coefficient *b*, Spearman’s rho, or the *t*-statistic from a regression or *t*-test. These metrics were then converted into Pearson’s *r* using R.

Effect sizes reported as Cohen’s *d* were converted into Pearson’s *r* using the function d_to_r() in the ‘effectsize’ package^68^. Spearman’s rho was transformed into Pearson’s *r* using the conversion formula^69^. The *t*-statistic from regression was converted using the t_to_r() function from the ‘effectsize’ package, and the *t*-statistic from a *t*-test was converted using esc_t() from the ‘esc’ package. The standardized regression coefficient *β* was transformed based on a widely used formula for effect sizes whose absolute value did not exceed 0.5 (ref. ^70^). For *β* values above this threshold, the function esc_beta() from the ‘esc’ package^71^ was used. To convert the non-standardized regression coefficient *b*, two approaches were used. Effect sizes from categorical SES variables were converted to Pearson’s *r* using the esc_B() function from the ‘esc’ package. To convert effect sizes from continuous variables, the non-standardized *b* was standardized by multiplying it by the standard deviation (SD) of the predictor variable (that is, SES measure) and dividing it by the s.d. of the outcome variable (that is, biological age acceleration). This standardized regression coefficient was then converted into Pearson’s *r* as described above.

If s.ds of SES or age acceleration measures were not reported in a study, various methods were used to impute the s.ds. For studies reporting age acceleration and/or SES measure’s s.ds for different groups (for example, men and women, white- and Black-identifying), the pooled s.ds for the whole sample were calculated, assuming equal variances of subgroups. For studies reporting interquartile ranges (IQR), the IQR was transformed into s.d. by dividing the IQR by 1.35 (ref. ^72^), assuming the outcome s.d. is similar to the normal distribution. For studies reporting means and s.ds of income/wealth variables in descriptives, but log-transforming them for the analysis, the s.d. of the log-transformed data was calculated using the formula reported in ref. ^73^.

If s.ds of age acceleration measures were not reported, they were taken from another study using the same cohort. The studies with the highest sample size, and the ones closest in age to the target sample size were prioritized. If this was not available, s.ds for adulthood and childhood age acceleration measures were taken from other studies that matched in tissue. For effect sizes reporting on age acceleration measured in adults from blood samples, s.ds were taken from Health and Retirement Study. The s.ds for Horvath Pan-Tissue, Horvath Skin and Blood, Hannum, PhenoAge, GrimAge and DunedinPoAm were taken from ref. ^74^. DunedinPACE was taken from ref. ^18^. The s.d. for Zhang clock was taken from the Multi-Ethnic Study on Atherosclerosis^75^.

SDs for children’s saliva were imputed using data from the Future of Families and Child Wellbeing Study (previously called Fragile Families and Child Wellbeing Study, FFCW). Methylation data processing is described in the ‘biomarker Data’ file on the FFCW documentation website. s.d were calculated by author Y.W. based on data access^23^. For the remaining childhood samples based on venous blood, cord blood or buccal tissue, s.d. were taken from other studies matching in tissue. If this was not possible, authors were emailed. Finally, if s.ds were not reported and could not be taken from another study, authors were emailed. Six authors provided relevant information.

Once the Pearson’s *r* values were calculated for each effect size, they were converted into Fisher’s *z* for analyses using the convert_r2z() function from the ‘esc’ package. This was done to eliminate bias due to sampling distributions, as the distribution of Fisher’s *z* approximates a normal distribution^67^. After the analyses, the *z*-scores were converted back to Pearson’s *r* using the convert_z2r() function from the ‘esc’ package for interpretation.

Fourteen effect sizes as biweight midcorrelation (bicor) estimates. As there is not a direct transformation from bicor to Pearson’s *r*, effect sizes reporting bicor values were meta-analysed separately and reported in Supplementary Fig. 16.

### Statistical analyses

The analyses were conducted using R’s metafor package^76^. A three-level mixed-effects model was used to accommodate the dependency of effect sizes within and across studies^77^, as some studies use the same sample and/or report multiple effect sizes. The study is nested within-sample to deal with potentially non-independent effect sizes coming from the same article or the same sample of participants. This allowed us to include all effect sizes of included studies, while taking the dependency of studies using data of the same cohort into account. In the three-level meta-analytic approach, level 1 accounts for the sampling variance of effect sizes, level 2 considers the variance among effect sizes within the same sample, and level 3 addresses the variance between studies^78^. This allowed the inclusion of all studies while ensuring that studies reporting multiple effect sizes will not have a greater contribution to the mean effect size than those reporting only one effect size^9^. These analysis steps have been used in previous meta-analyses including DNAm measures^79^. This analysis was carried out based on the guidelines by Assink and Wibbelink^80^.

#### Main analyses

Social determinants of health measures were separated into SES and race and ethnicity. The following steps were performed for SES and each race and ethnicity category. First, we assessed whether the association between SES, race and ethnicity measure and epigenetic clock-measured biological ageing differs for first-, second- and third-generation clock measures (Table 2). We then investigated whether the association between SES, race and ethnicity measures and clock measures differs for specific clocks, if there were a minimum of 5 effect sizes for a given clock (as with a small number of studies, statistical power is low^81^). Then, likelihood-ratio tests were conducted to establish the within- and between-cohort heterogeneity separately for the associations of first-, second- and third-generation clocks and SES. Significant heterogeneity at these two levels would suggest evidence for variance that cannot be solely attributed to sampling variance^80^.

#### Moderator analyses

We conducted moderator analyses when effect-size heterogeneity was present and at least five effect sizes were available per category. To be more inclusive, we deviated from our preregistration (which required five studies) and instead used a threshold of five effect sizes per category. We also prioritized the timing of DNAm measurement as the most empirically meaningful moderator.

The moderator analyses were carried out as follows: If there were enough effect sizes for each individual DNAm measure, we assessed whether the moderators (Table 2) influenced the association between SES, race and ethnicity and DNAm measures if there were at least five effect sizes reported for a given category of the moderator.

#### Risk of bias

We evaluated potential risk of bias using multiple complementary approaches. We generated funnel plots and conducted Egger’s regression tests to assess systematic bias and applied trim-and-fill analyses to estimate the influence of potentially missing studies. To test the robustness of our findings, we re-ran all meta-analyses (1) excluding preprints (non–peer-reviewed studies), (2) excluding previous meta-analyses, (3) excluding the largest contributing cohort (the Health and Retirement Study) and (4) excluding effect sizes derived from small samples (*n* < 100). In addition, we conducted Leave-one-cohort-out analyses for 19 influential cohorts—defined as those included in ≥3 studies or contributing ≥25 effect sizes—by sequentially removing each cohort and re-estimating the associations. Note that Egger’s and trim-and-fill estimates are not based on the three-level meta-analysis reported in the main results, as this method is not yet adjusted for multi-level meta-analyses^80^.

### Ethics statement

This study is a systematic review and meta-analysis based exclusively on data reported in previously published studies. No new data were collected, and no direct interaction with human participants occurred. Therefore, local ethics approval and participant consent were not required for the present study. As the study synthesizes published findings, it does not introduce additional risks to participants, researchers or communities beyond those present in the original studies. Roles and responsibilities among co-authors were agreed upon before conducting the study.

### Reporting summary

Further information on research design is available in the Nature Portfolio Reporting Summary linked to this article.

## Supplementary information

Supplementary Information Supplementary figures.

Reporting Summary

Peer Review File

Supplementary Tables Supplementary Tables.

## Data Availability

All data analysed in this study were derived from previously published studies included in the systematic review and meta-analysis. Extracted data and R-code are publicly available in the project repository at https://github.com/Biosocial/Meta-SDoH-EpigeneticClocks.
