# Supplementary material for: Social determinants of health and epigenetic clocks: a systematic review and meta-analysis of 140 studies
Source: Nat Hum Behav. 2026 Jun 12;10(10):2016–28. doi: 10.1038/s41562-026-02477-6 (PMC13683040; doi:10.1038/s41562-026-02477-6)
Supplement: Supplementary file 1 [file 41562_2026_2477_MOESM1_ESM.pdf]

nature human behaviour

Supplementary information https://doi.org/10.1038/s41562-026-02477-6

Social determinants of health and epigenetic clocks: a systematic review and meta-analysis of 140 studies

In the format provided by the authors and unedited

Table of Contents

Supplemental Figure 1. World map showing the countries included in this meta-analysis...2

Supplemental Figure 2. Funnel plots to assess publication bias in the relationship between socioeconomic status and epigenetic-clock-measured biological aging...3

Supplemental Figure 3. Meta-analytic associations between socioeconomic status (SES) and epigenetic clocks, excluding effect sizes from the Health and Retirement Study (HRS)...4

Supplemental Figure 4. Meta-analytic associations between socioeconomic status (SES) and epigenetic clocks, excluding effect sizes from meta-analyses...5

Supplemental Figure 5. Meta-analytic associations between socioeconomic status (SES) and epigenetic clocks, excluding effect sizes from preprints...6

Supplemental Figure 6. Meta-analytic associations between socioeconomic status (SES) and epigenetic clocks, excluding effect sizes with small sample sizes (n <100)...7

Supplemental Figure 7. Meta-analytic associations between intergenerational socioeconomic status (SES) and epigenetic clocks...8

Supplemental Figure 8. Associations between repeated measures of socioeconomic status (SES) and epigenetic clocks...9

Supplemental Figure 9. Funnel plots to assess publication bias in the relationship between Black-White comparisons and epigenetic-clock-measured biological aging...11

Supplemental Figure 10. Funnel plots to assess publication bias in the relationship between Latinx-White comparisons and epigenetic-clock-measured biological aging...12

Supplemental Figure 11. Meta-analytic associations between race/ethnicity and epigenetic clocks, excluding effect sizes from preprints...13

Supplemental Figure 12. Meta-analytic associations between race/ethnicity and epigenetic clocks, excluding effect sizes with small sample sizes (n<100)...15

Supplemental Figure 13. Meta-analytic associations between race/ethnicity and epigenetic clocks, excluding effect sizes from the Health and Retirement Study...17

Supplemental Figure 14. Meta-analytic associations between socioeconomic status (SES) and epigenetic clocks, separately for EPIC and 450k arrays...19

Supplemental Figure 15. Heatmap showing the number of effect sizes for epigenetic-clock-measured biological aging (x-axis) and social determinants of health (y-axis)...21

Supplemental Figure 16. Meta-analytic associations between socioeconomic status (SES) and epigenetic clocks – biweight midcorrelation (bicor) estimates...22

Supplemental Figure 17. Leave-One-Cohort-Out Analyses for the meta-analytic associations between SES and epigenetic clocks...33

Supplemental Figure 18. Leave-One-Cohort-Out Analyses for the meta-analytic associations between Black- and White-identifying groups and epigenetic clocks...35

Supplemental Figure 19. Leave-One-Cohort-Out Analyses for the meta-analytic associations between Latinx- and White-identifying groups and epigenetic clocks...38

Supplemental Figure 20. Pearson's r values for the associations between SES/race/ethnicity and DNAm generations and measures of biological aging 39

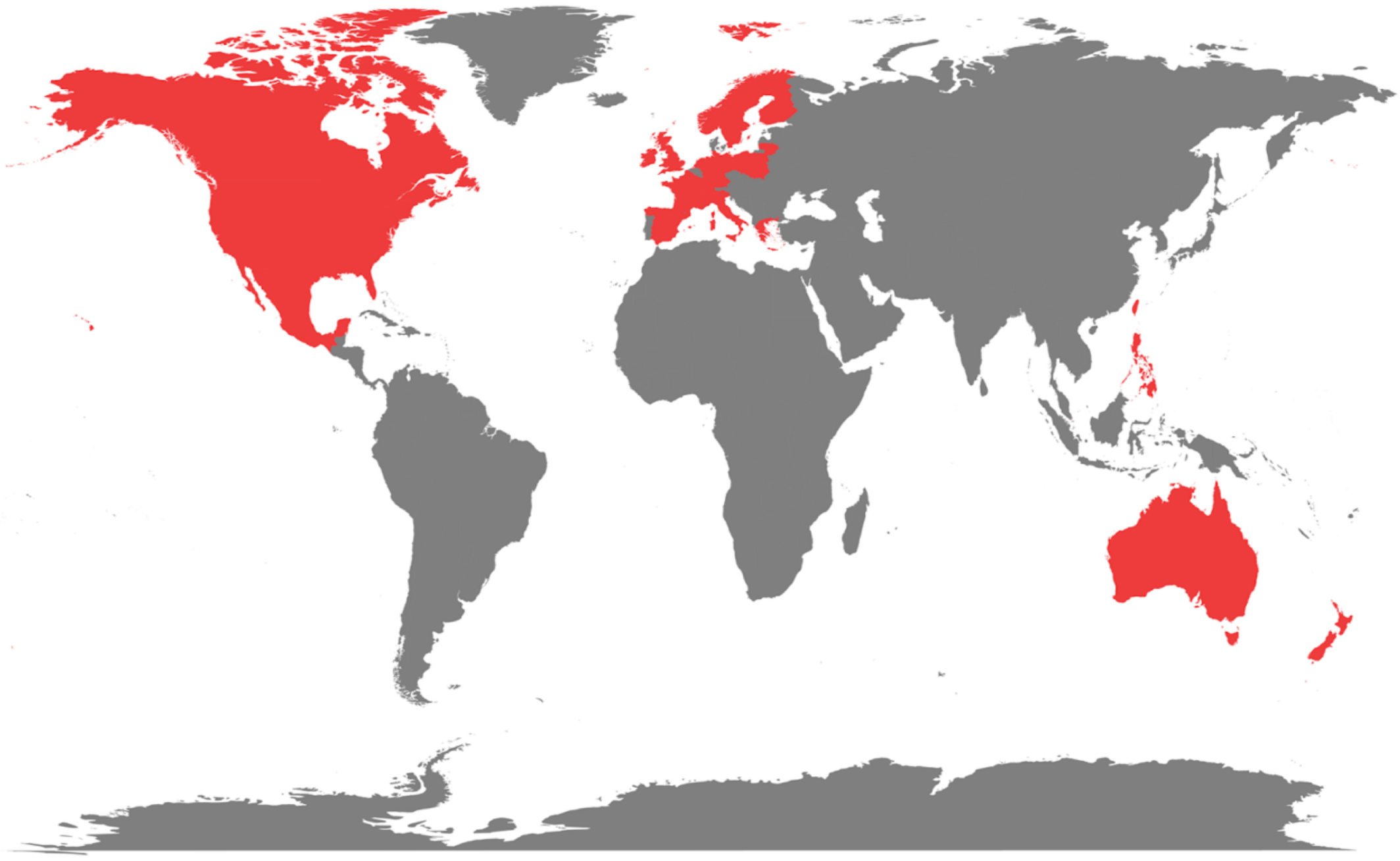

Supplemental Figure 1. World map showing the countries included in this meta-analysis. Red color indicates that there is at least one effect size from that country (including contributions to meta-analyses). Countries colored in gray do not contribute any effect sizes. Map data from Natural Earth (public domain), visualisation created by authors in R.

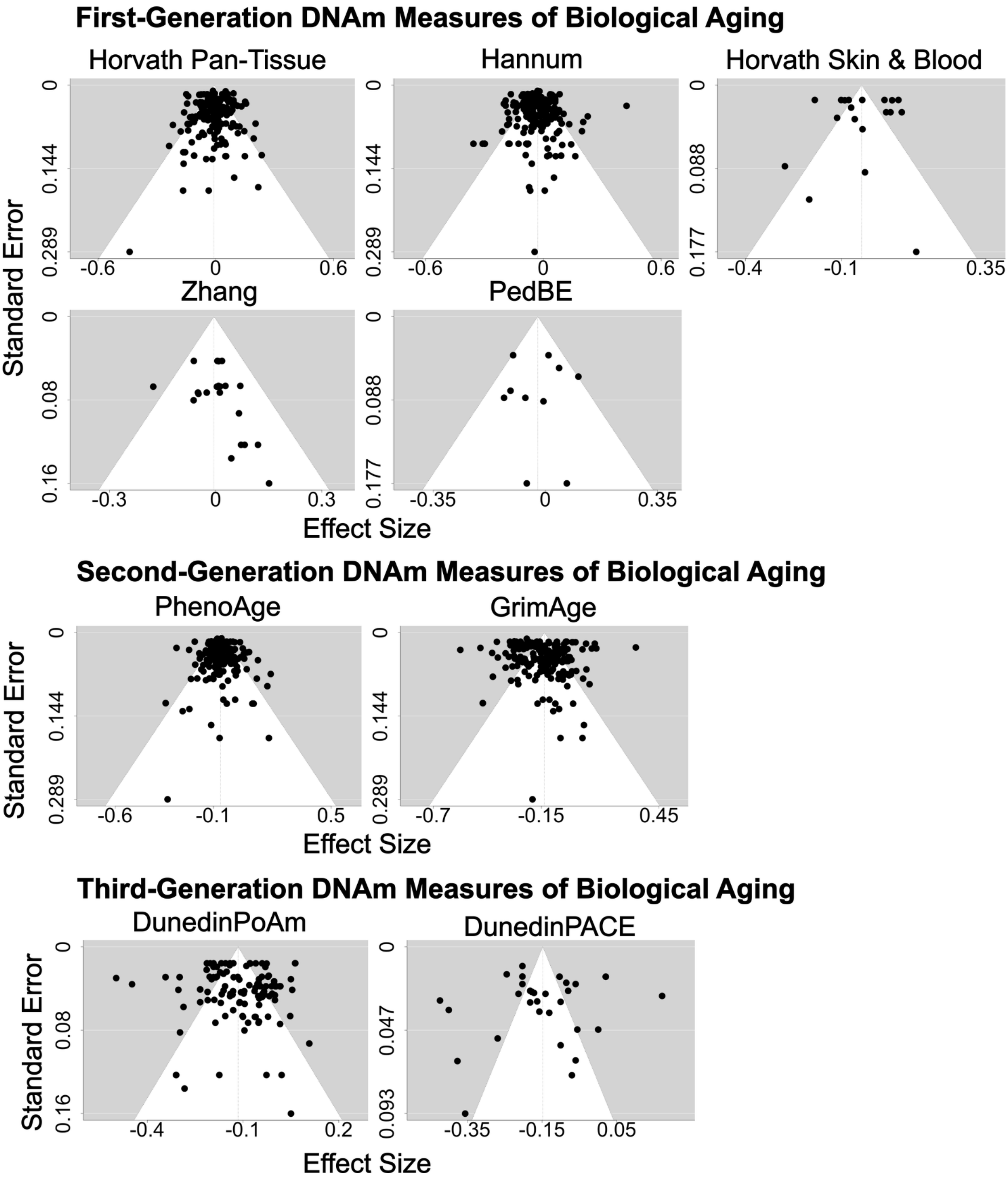

Supplemental Figure 2. Funnel plots to assess publication bias in the relationship between socioeconomic status and epigenetic-clock-measured biological aging.

These plots display individual effect sizes on the x-axis and their standard errors on the y-axis. According to the results of Egger's regression test, there is no evidence of publication bias for any of the epigenetic-clock-measured biological aging (all ps < 0.05; Supplemental Table 5A).

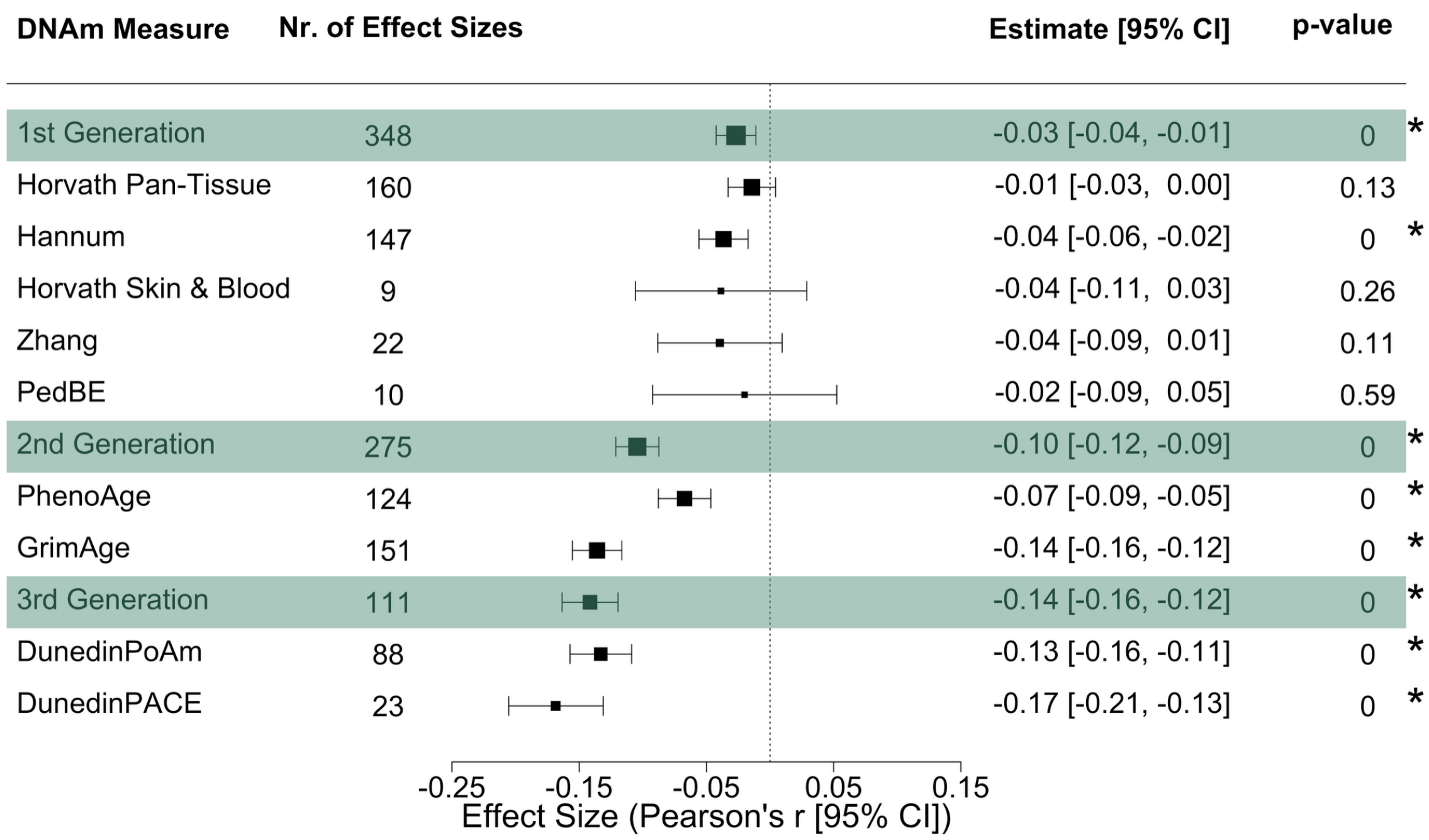

Supplemental Figure 3. Meta-analytic associations between socioeconomic status (SES) and epigenetic clocks, excluding effect sizes from the Health and Retirement Study (HRS). Forest plot depicts the meta-analytic associations between SES and generations of epigenetic clocks, after 174 effect sizes contributed by HRS are removed. Green panels indicate the meta-analyzed effect size estimates for first-, second-, and third-generation clocks, followed by individual clocks within each category. Analyses were conducted using two-sided Fisher-Z transformation and later transformed to Pearson's r to ease interpretation. Effect size estimates are bounded by 95% confidence intervals (CIs).* denotes significant difference from 0 at $p < 0.05$ (not corrected for multiple comparisons). The size of the boxes in the middle panel represents the precision of the effect size estimate (i.e., the smaller the CI, the more precise the estimate).

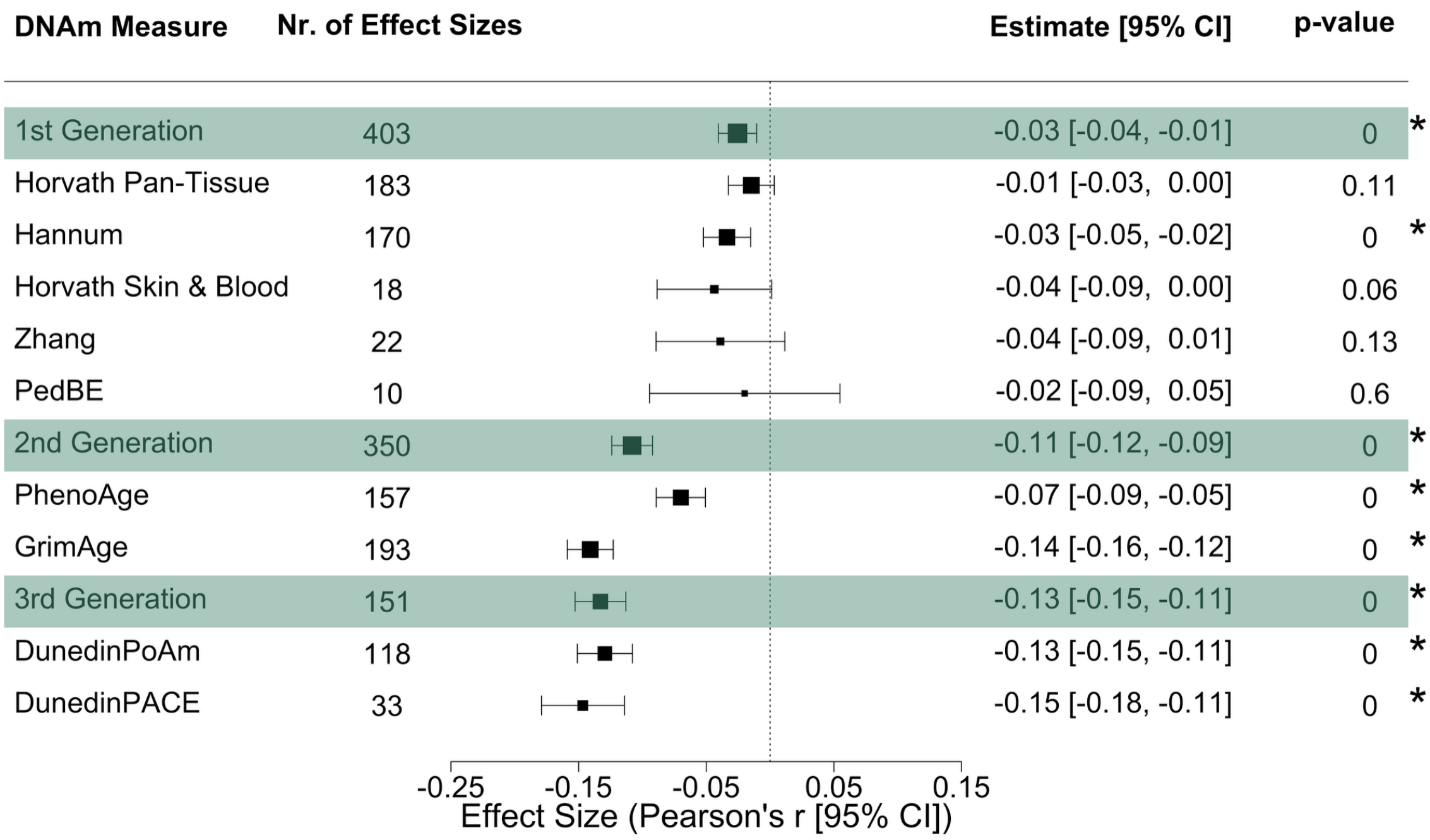

Supplemental Figure 4. Meta-analytic associations between socioeconomic status (SES) and epigenetic clocks, excluding effect sizes from meta-analyses. Forest plot depicts the meta-analytic associations between SES and generations of epigenetic clocks, after 4 effect sizes contributed by meta-analyses are removed. Green panels indicate the meta-analyzed effect size estimates for first-, second-, and third-generation clocks, followed by individual clocks within each category. Analyses were conducted using two-sided Fisher-Z transformation and later transformed to Pearson's r to ease interpretation. Effect size estimates are bounded by 95% confidence intervals (CIs).* denotes significant difference from 0 at $p < 0.05$ (not corrected for multiple comparisons). The size of the boxes in the middle panel represents the precision of the effect size estimate (i.e., the smaller the CI, the more precise the estimate).

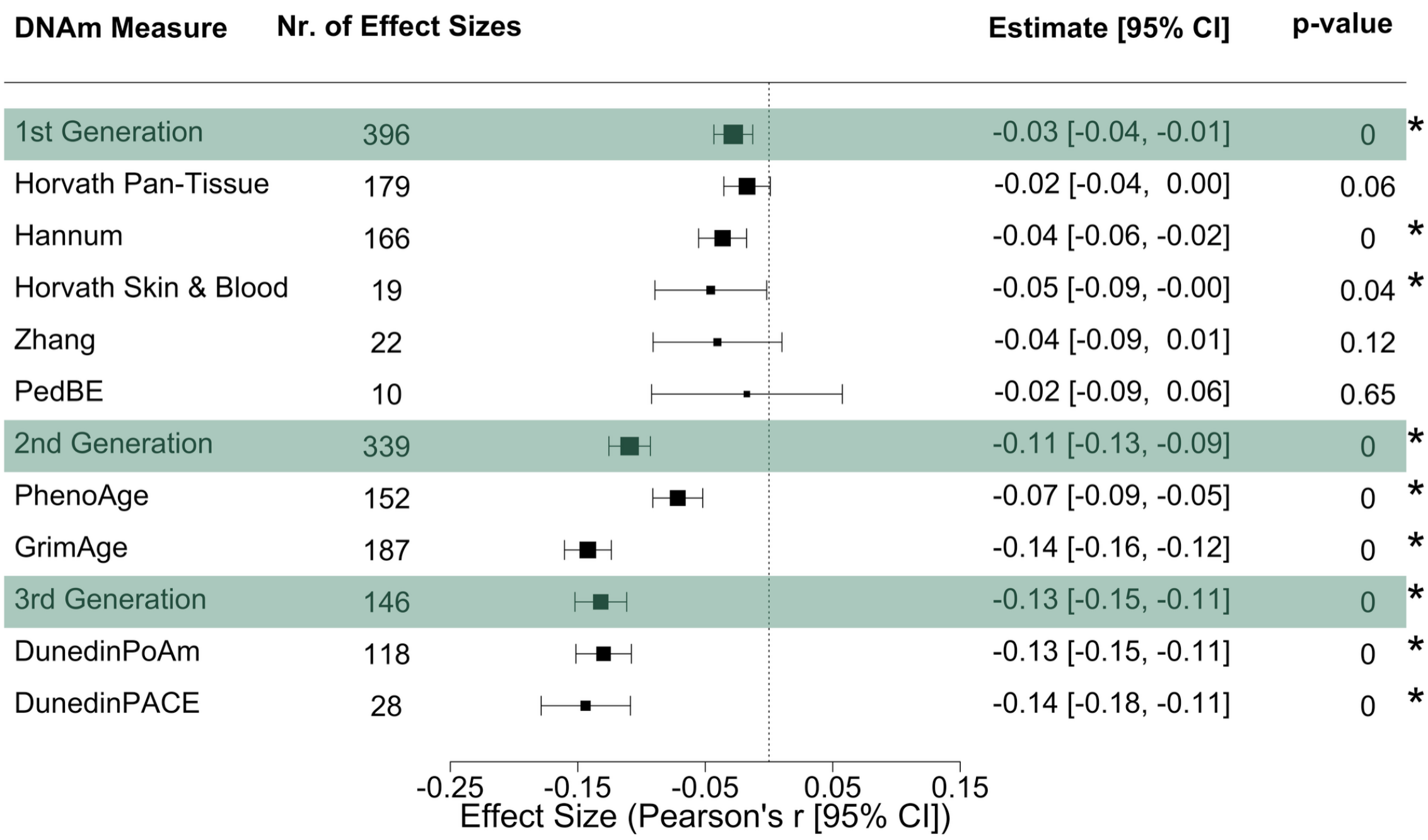

Supplemental Figure 5. Meta-analytic associations between socioeconomic status (SES) and epigenetic clocks, excluding effect sizes from preprints. Forest plot depicts the meta-analytic associations between SES and generations of epigenetic clocks, after 27 effect sizes contributed by preprints are removed. Green panels indicate the meta-analyzed effect size estimates for first-, second-, and third-generation clocks, followed by individual clocks within each category. Analyses were conducted using two-sided Fisher-Z transformation and later transformed to Pearson's r to ease interpretation. Effect size estimates are bounded by 95% confidence intervals (CIs).* denotes significant difference from 0 at $p < 0.05$ (not corrected for multiple comparisons). The size of the boxes in the middle panel represents the precision of the effect size estimate (i.e., the smaller the CI, the more precise the estimate).

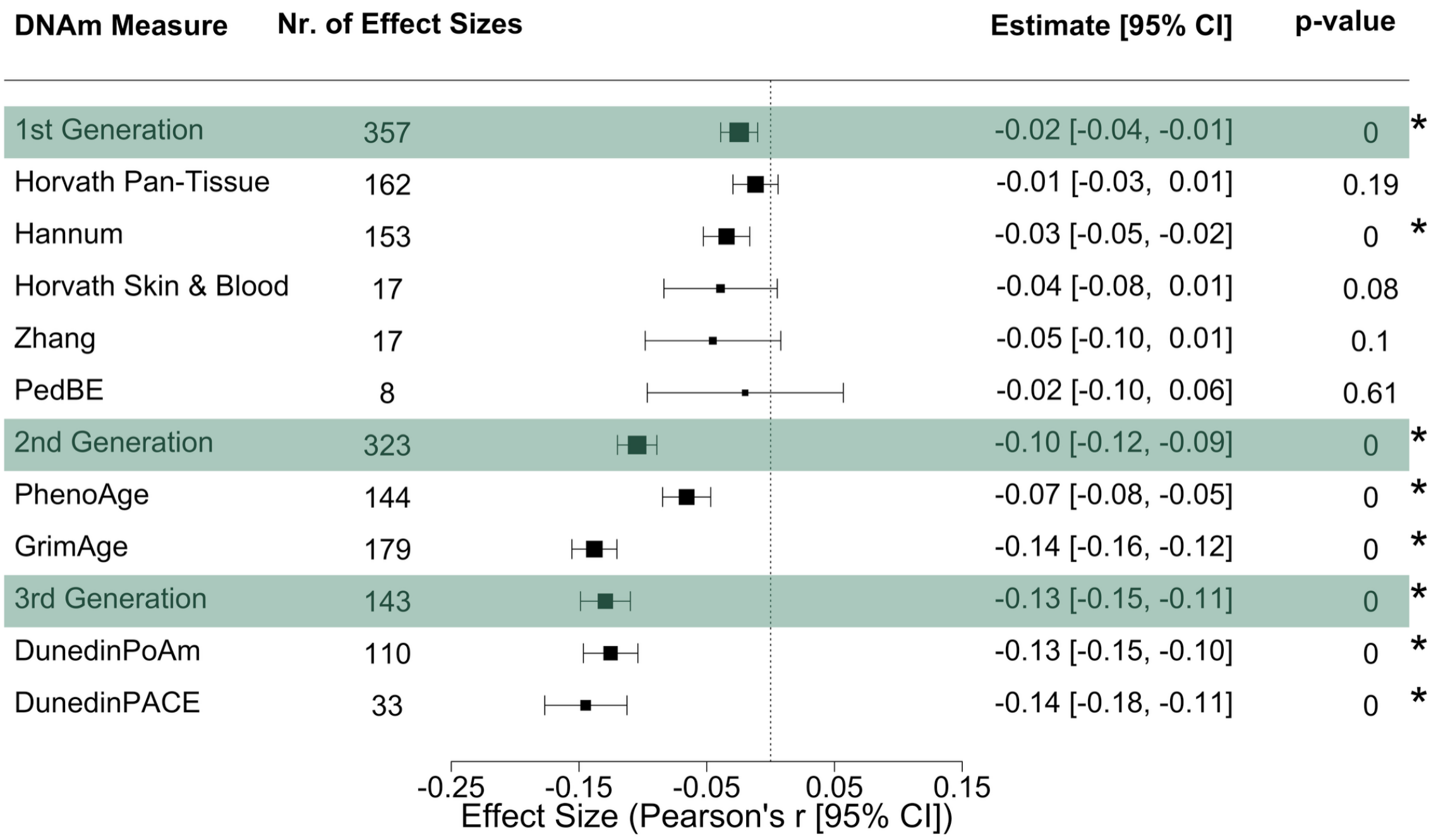

Supplemental Figure 6. Meta-analytic associations between socioeconomic status (SES) and epigenetic clocks, excluding effect sizes with small sample sizes (n <100).

Forest plot depicts the meta-analytic associations between SES and generations of epigenetic clocks, after 85 effect sizes with small sample sizes (n<100) are removed. Green panels indicate the meta-analyzed effect size estimates for first-, second-, and third-generation clocks, followed by individual clocks within each category. Analyses were conducted using two-sided Fisher-Z transformation and later transformed to Pearson's r to ease interpretation. Effect size estimates are bounded by 95% confidence intervals (CIs).* denotes significant difference from 0 at $p < 0.05$ (not corrected for multiple comparisons). The size of the boxes in the middle panel represents the precision of the effect size estimate (i.e., the smaller the CI, the more precise the estimate).

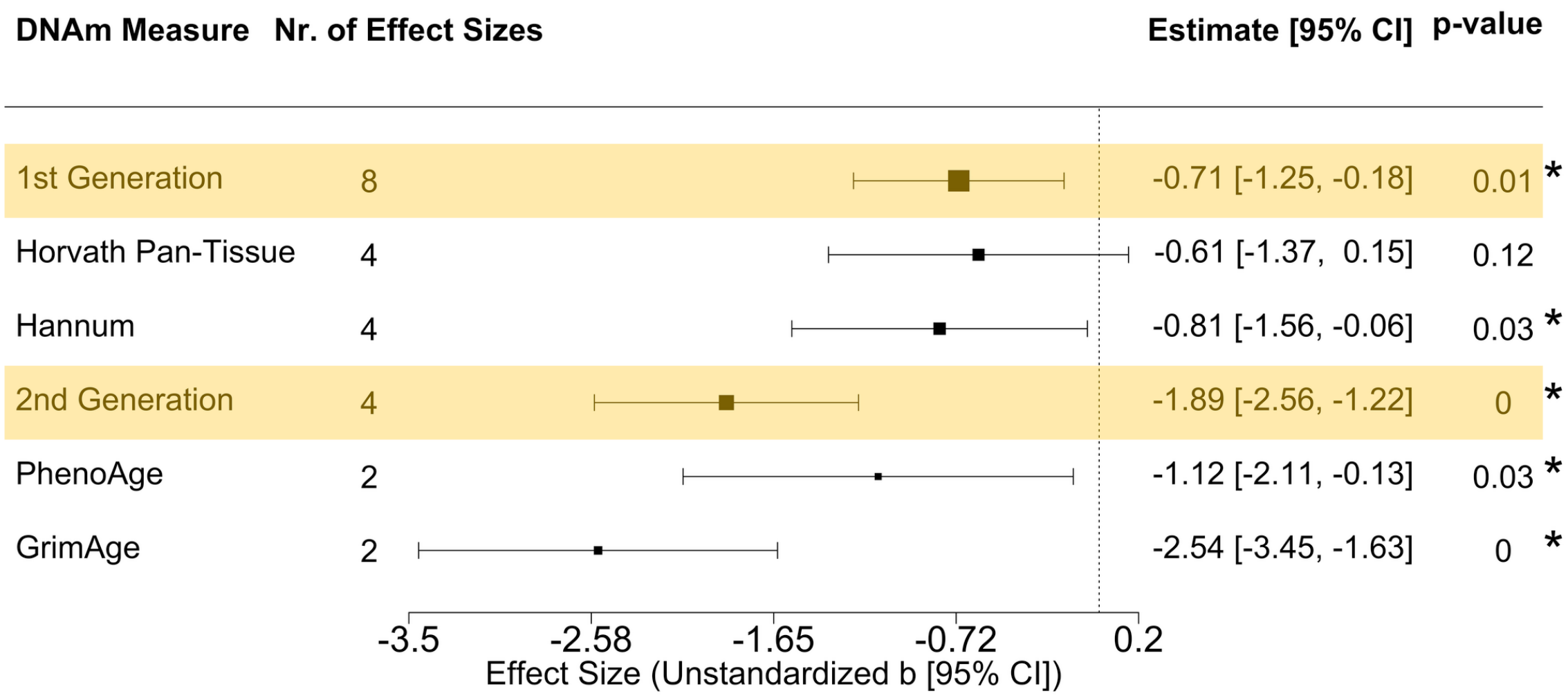

Supplemental Figure 7. Meta-analytic associations between intergenerational socioeconomic status (SES) and epigenetic clocks. Forest plot depicts the meta-analytic associations between intergenerational SES and epigenetic clocks. DNAm was measured at one time point. Intergenerational SES is measured by the intergenerational stability in SES, comparing parental occupation with current occupation, where the reference group is stably low SES and the target group is stably high SES. Yellow panels indicate the meta-analyzed effect size estimates for first- and second-generation clocks, followed by individual clocks within each category. Results are meta-analyzed as unstandardized regression coefficients using two-sided hypothesis testing. Effect size estimates are bounded by 95% confidence intervals (CIs).* denotes significant difference from 0 at $p < 0.05$ (not corrected for multiple comparisons). The size of the boxes in the middle panel represents the precision of the effect size estimate (i.e., the smaller the CI, the more precise the estimate).

A. Associations between socioeconomic status (SES) and repeated measures of epigenetic clocks within individuals

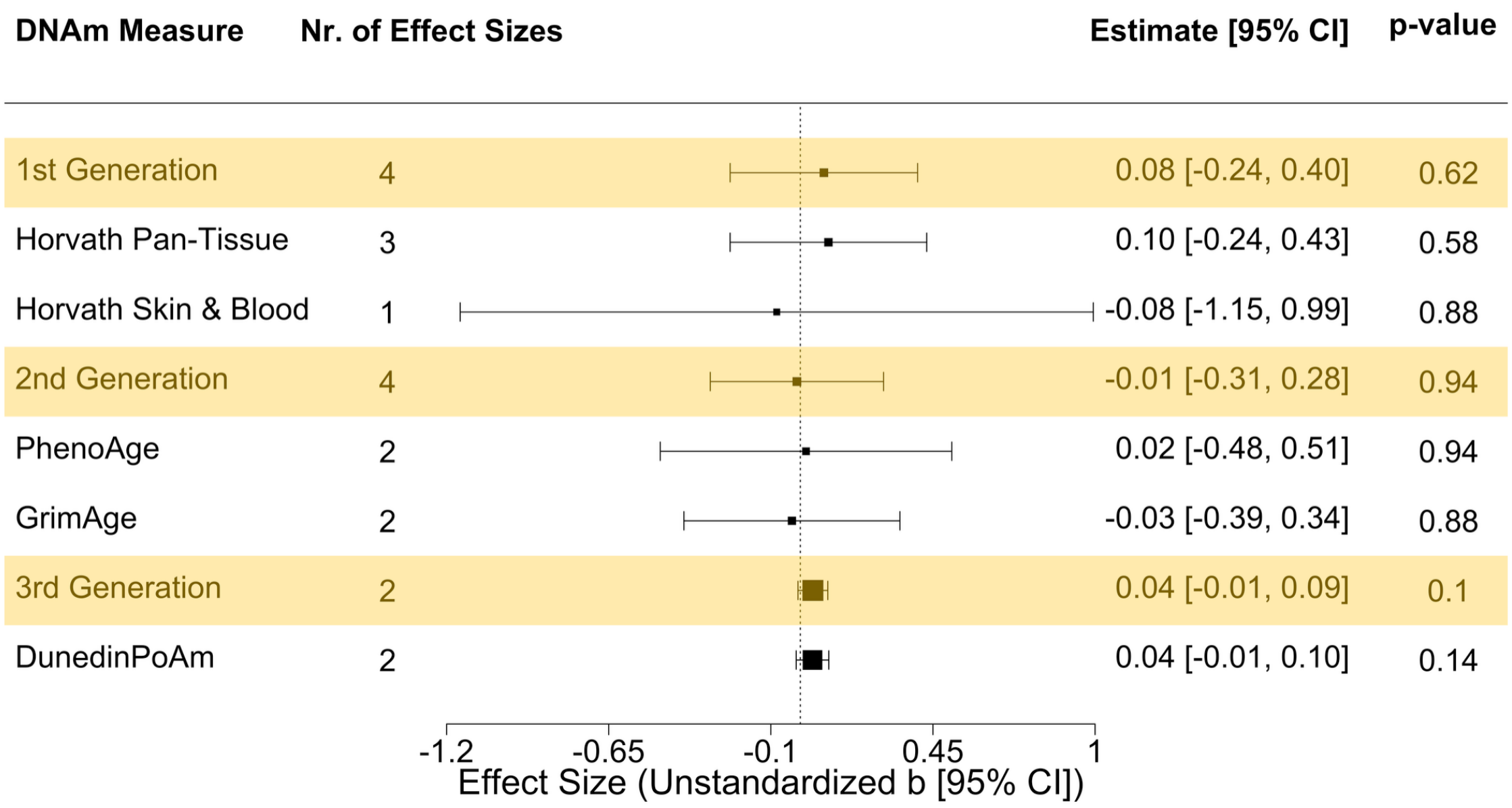

B. Associations between repeated measures of SES and repeated measures of epigenetic clocks within individuals

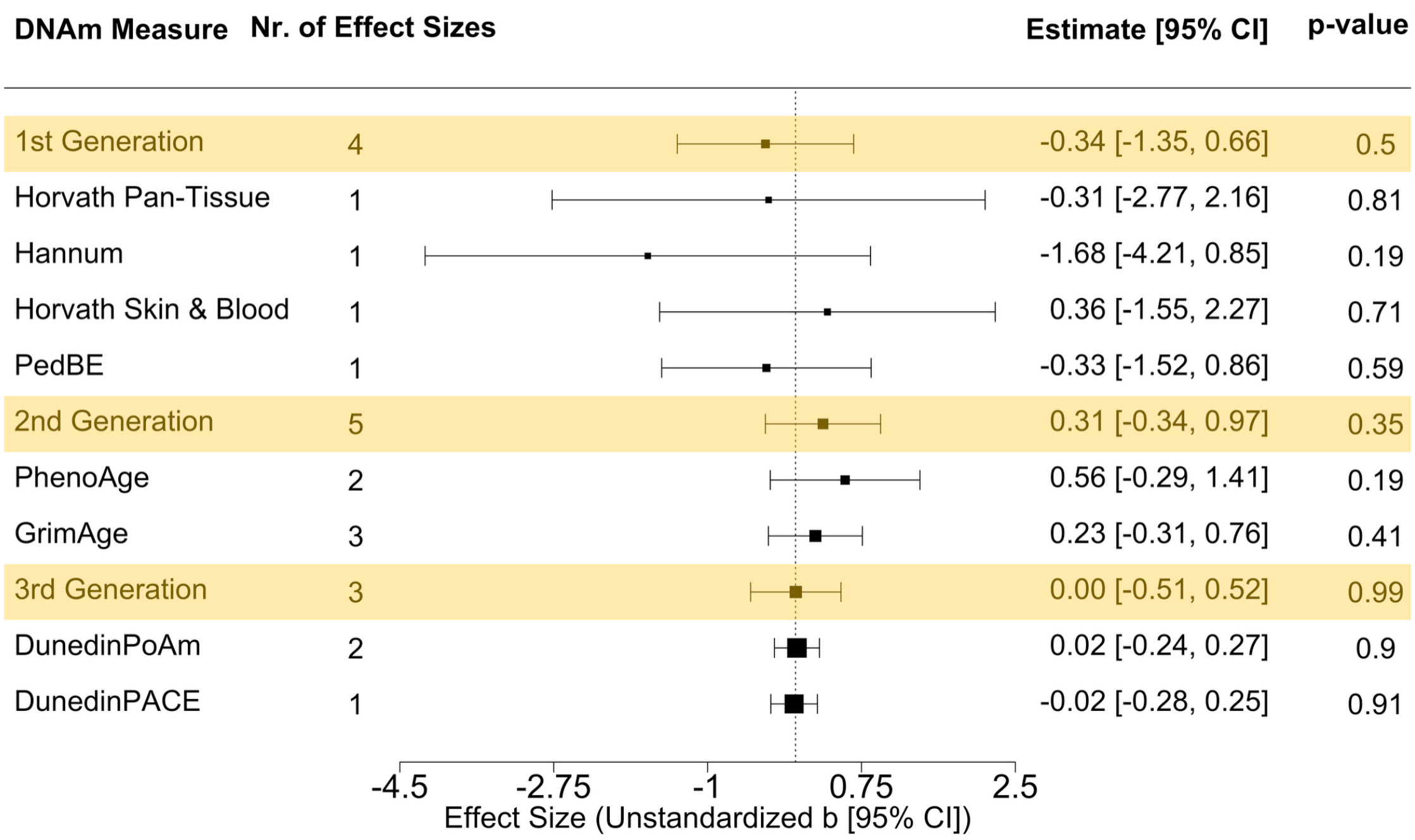

Supplemental Figure 8. Associations between repeated measures of socioeconomic status (SES) and epigenetic clocks. Forest plot in section A depicts the meta-analytic associations between SES and repeated measures of epigenetic clocks within individuals. Forest plot in section B depicts the meta-analytic associations of repeated measures of SES and repeated measures of epigenetic clocks within individuals. Yellow panels indicate the meta-analyzed effect size estimates for first-, second-, and third-generation clocks, followed by individual clocks within each category. As effect sizes for change data are mainly reported as unstandardized regression coefficients, and SDs for change in age acceleration and SES are not reported in any of the studies, change results are meta-analyzed as unstandardized regression coefficients separately using two-sided hypothesis testing. Effect size estimates are bounded by 95% confidence intervals (CIs).* denotes significant difference from 0 at $p < 0.05$ (not corrected for multiple comparisons). The size of the boxes in the middle panel represents the precision of the effect size estimate (i.e., the smaller the CI, the more precise the estimate).

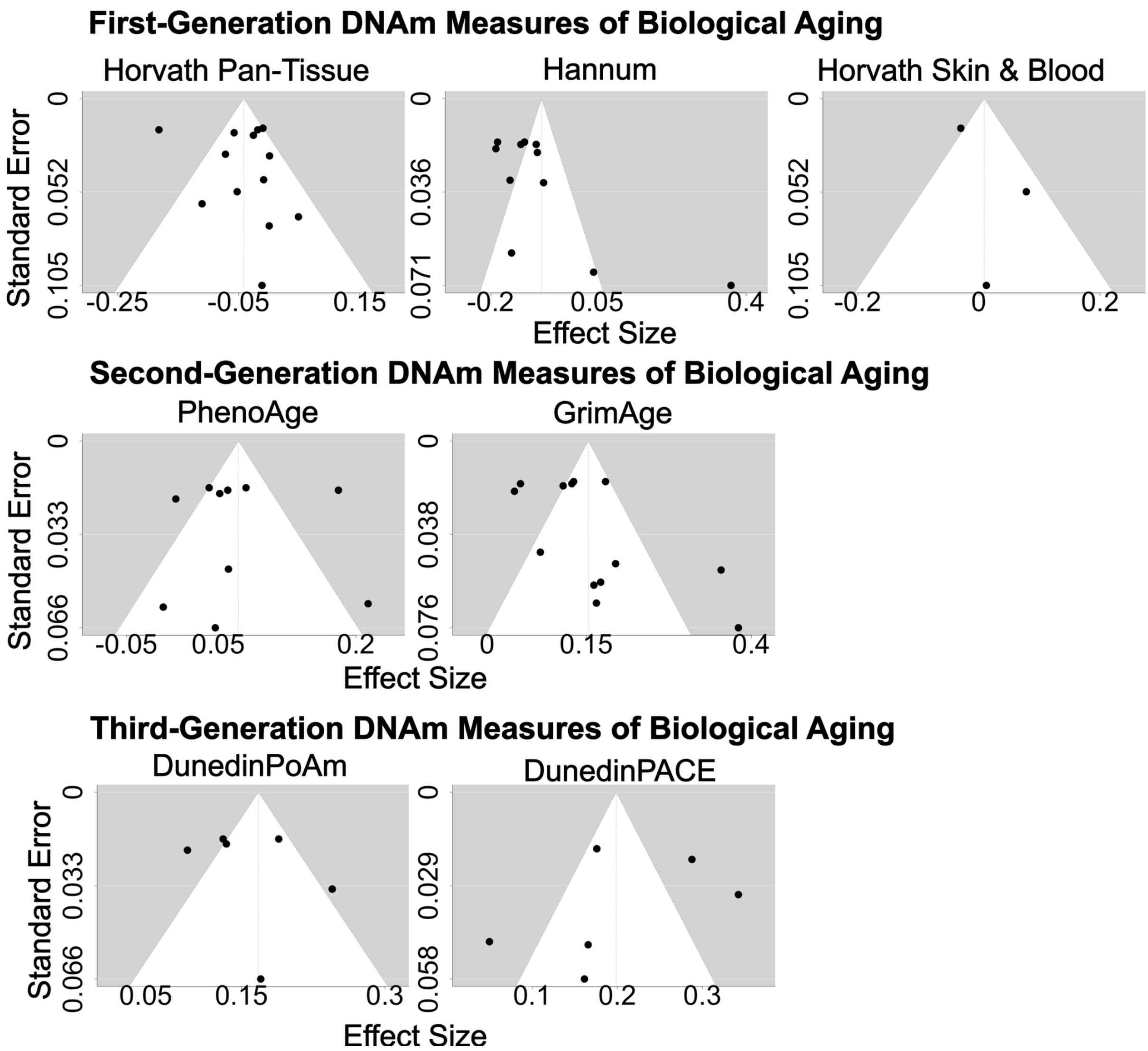

Supplemental Figure 9. Funnel plots to assess publication bias in the relationship between Black-White comparisons and epigenetic-clock-measured biological aging.

These plots display individual effect sizes on the x-axis and their standard errors on the y-axis. Tests could not be run for Zhang clock due to model constraints. Results of Egger's regression tests can be found in Supplemental Table 5C.

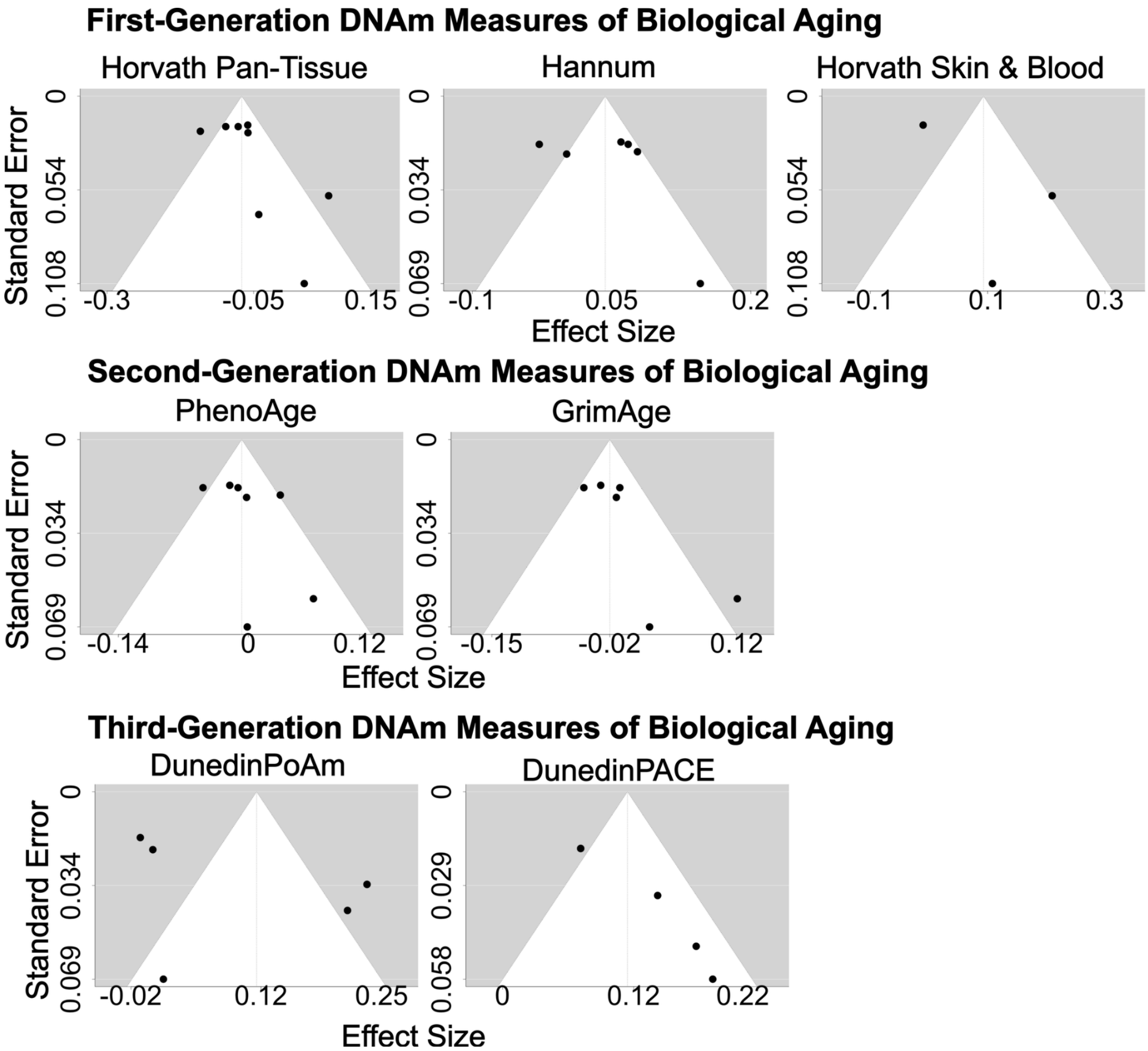

Supplemental Figure 10. Funnel plots to assess publication bias in the relationship between Latinx-White comparisons and epigenetic-clock-measured biological aging.
These plots display individual effect sizes on the x-axis and their standard errors on the y-axis. Results of Egger's regression tests can be found in Supplemental Table 5E.

A. Meta-analytic associations between Black-White comparisons and epigenetic clocks (excluding preprints)

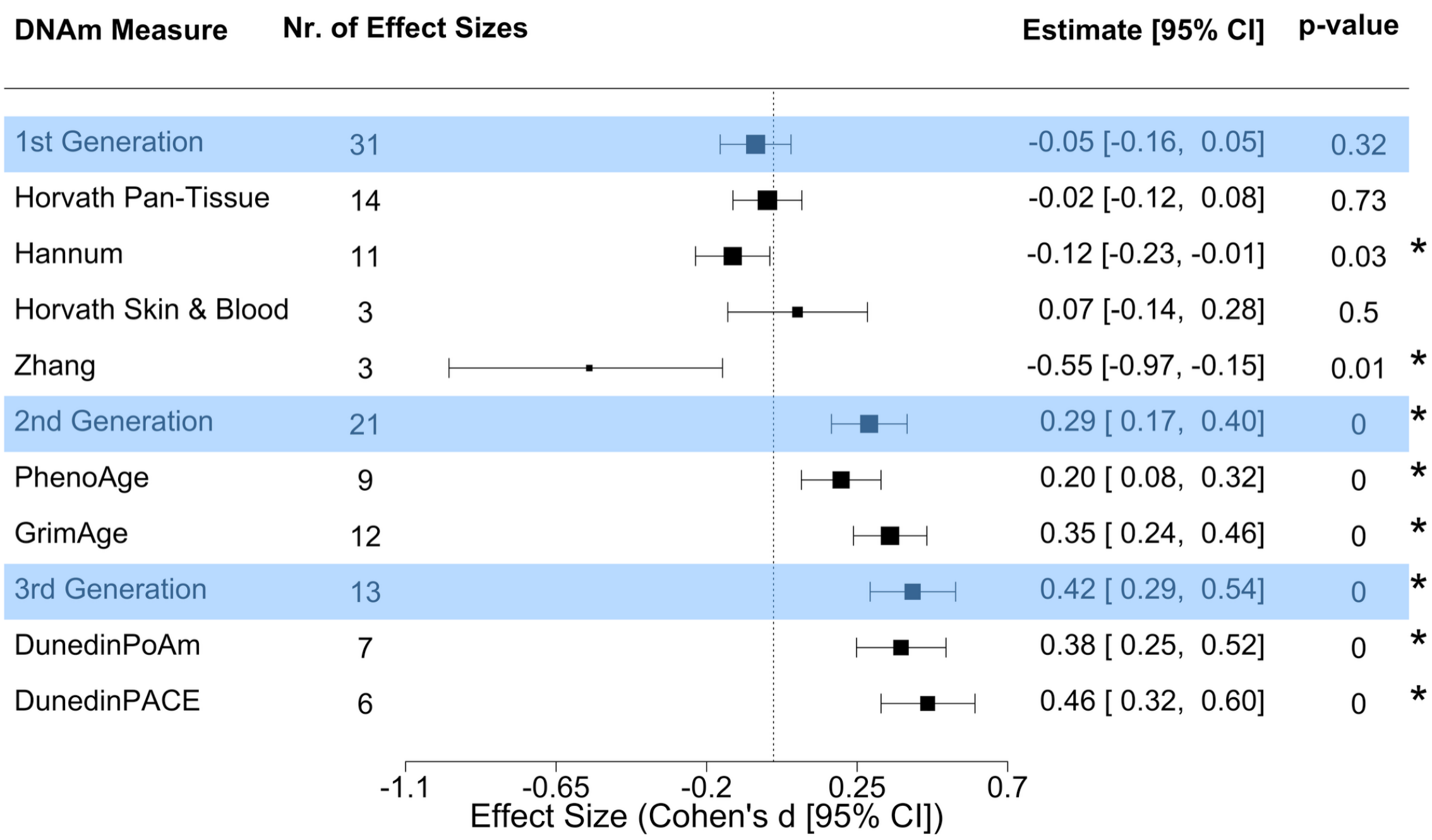

B. Meta-analytic associations between Latinx-White comparisons and epigenetic clocks (excluding preprints)

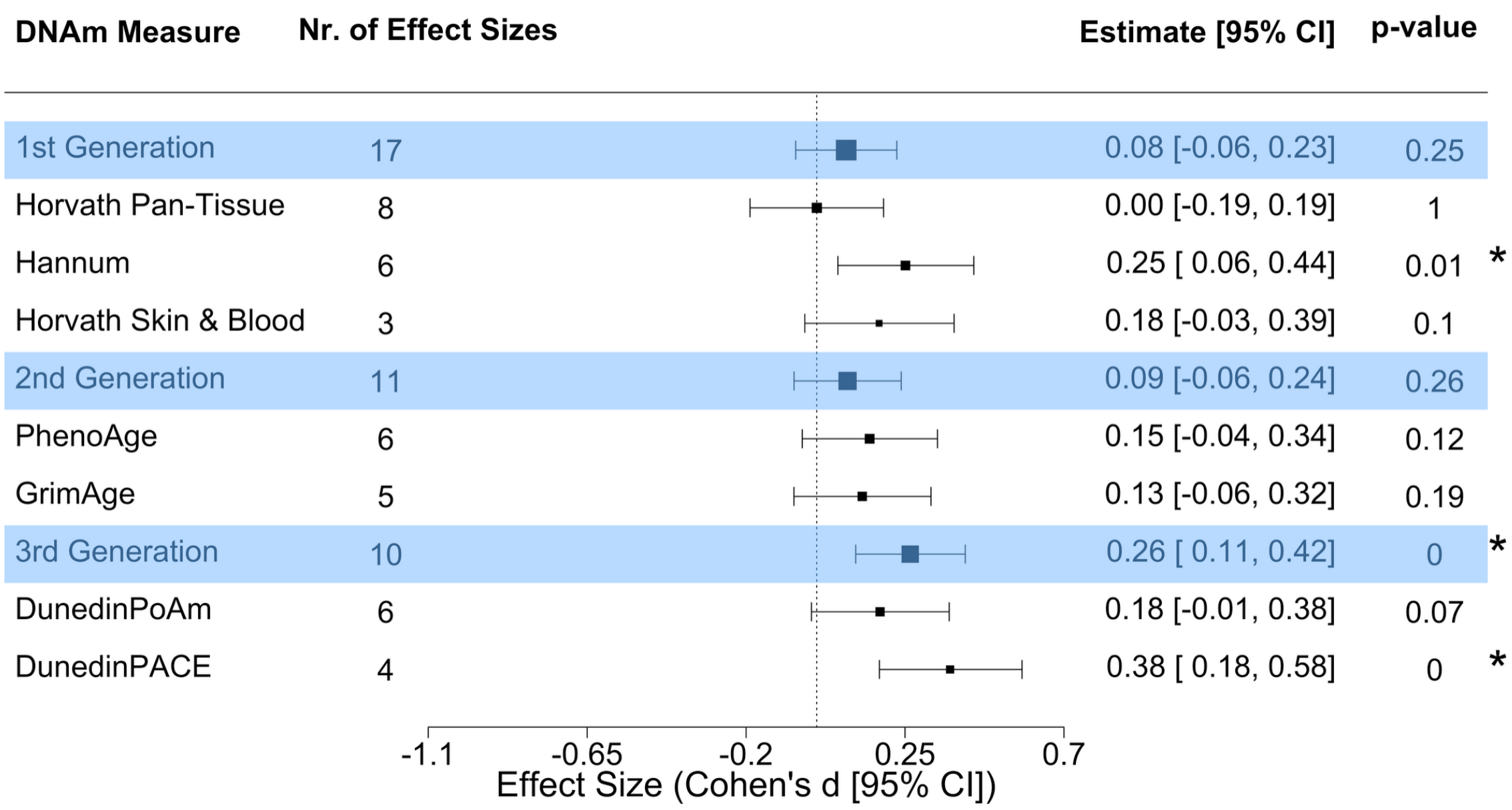

Supplemental Figure 11. Meta-analytic associations between race/ethnicity and epigenetic clocks, excluding effect sizes from preprints. Forest plot depicts the meta-analytic associations between (A) Black-White comparisons and generations of epigenetic clocks and (B) Latinx-White comparisons and generations of epigenetic clocks, after 3 effect sizes contributed by preprints are removed from both datasets. Blue panels indicate the meta-analyzed effect size estimates for first-, second-, and third-generation clocks, followed by individual clocks within each category. Analyses were conducted using two-sided Fisher-Z transformation and later transformed to Cohen's d to ease interpretation. Effect size estimates are bounded by 95% confidence intervals (CIs).* denotes significant difference from 0 at $p < 0.05$ (not corrected for multiple comparisons). The size of the boxes in the middle panel represents the precision of the effect size estimate (i.e., the smaller the CI, the more precise the estimate).

A. Meta-analytic associations between Black-White comparisons and epigenetic clocks (excluding effect sizes with small sample sizes)

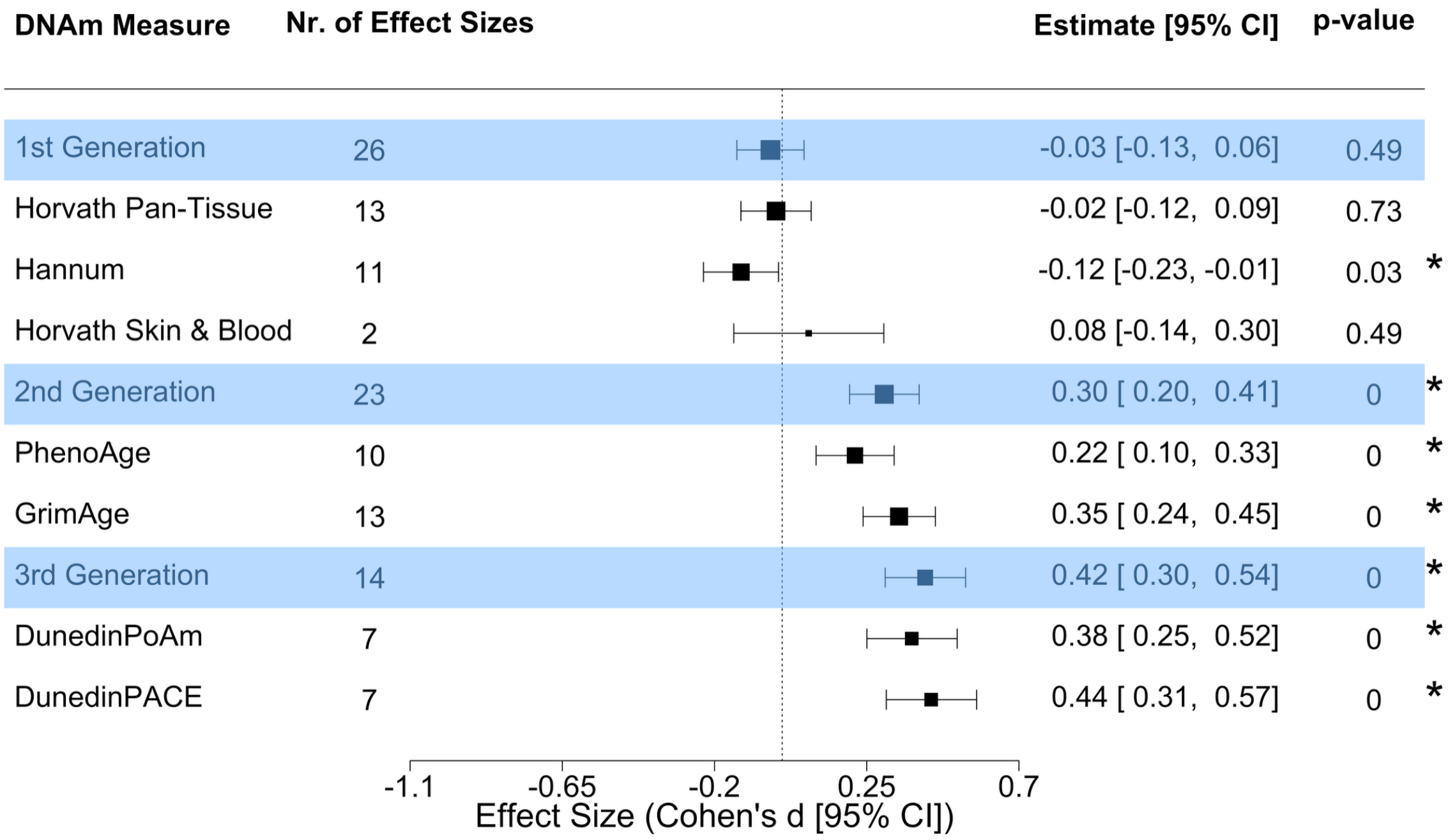

B. Meta-analytic associations between Latinx-White comparisons and epigenetic clocks (excluding effect sizes with small sample sizes)

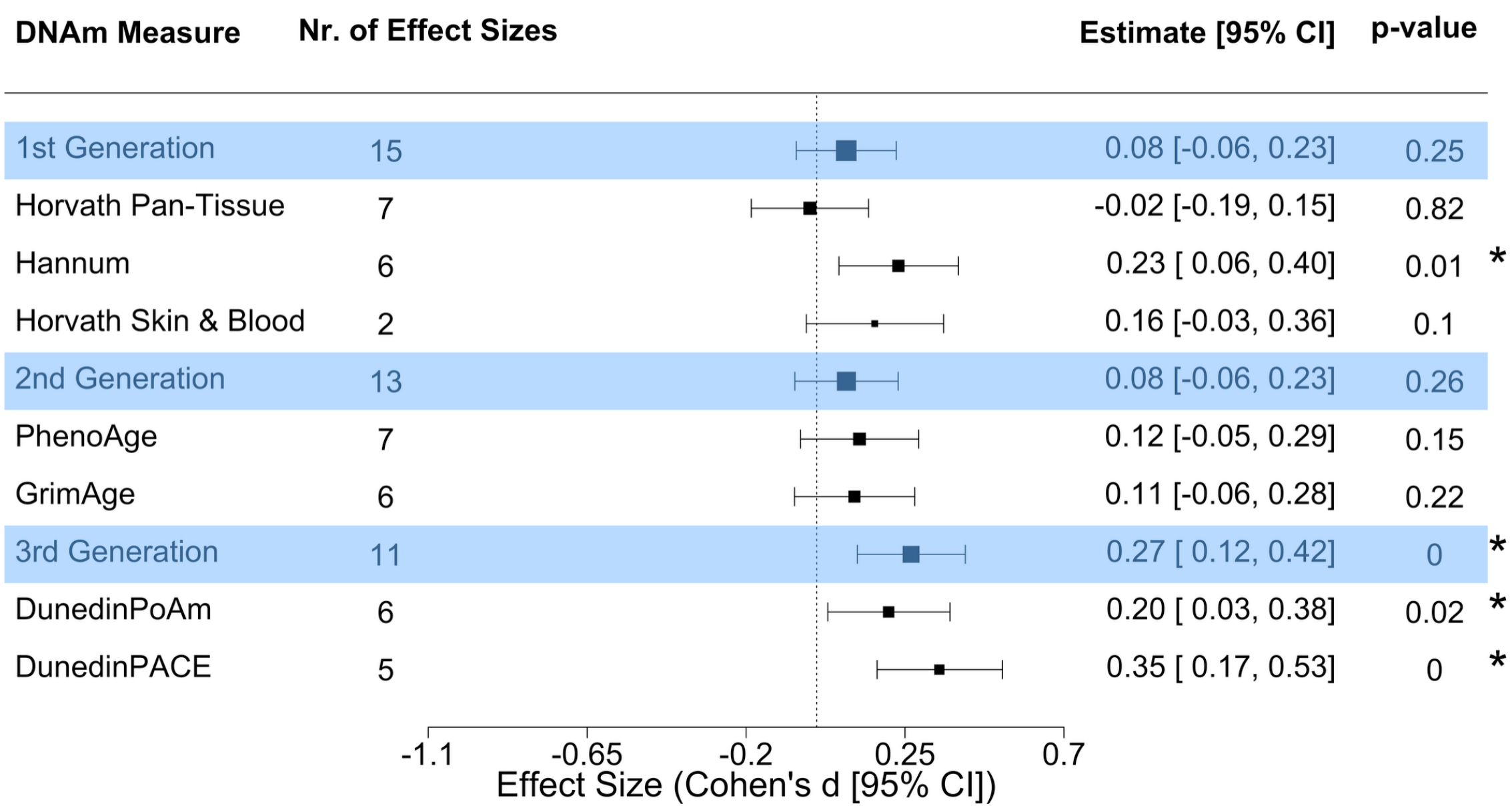

Supplemental Figure 12. Meta-analytic associations between race/ethnicity and epigenetic clocks, excluding effect sizes with small sample sizes (n<100). Forest plot depicts the meta-analytic associations between (A) Black-White comparisons and generations of epigenetic clocks and (B) Latinx-White comparisons and generations of epigenetic clocks, after 5 and 2 effect sizes with small sample sizes

are removed, respectively. Blue panels indicate the meta-analyzed effect size estimates for first-, second-, and third-generation clocks, followed by individual clocks within each category. Analyses were conducted using two-sided Fisher-Z transformation and later transformed to Cohen's d to ease interpretation. Effect size estimates are bounded by 95% confidence intervals (CIs).* denotes significant difference from 0 at $p < 0.05$ (not corrected for multiple comparisons). The size of the boxes in the middle panel represents the precision of the effect size estimate (i.e., the smaller the CI, the more precise the estimate).

A. Meta-analytic associations between Black-White comparisons and epigenetic clocks (excluding effect sizes from the Health and Retirement Study)

DNAm Measure	Nr. of Effect Sizes	Estimate [95% CI]	p-value	
1st Generation	20	0.01 [-0.14, 0.16]	0.9	
Horvath Pan-Tissue	9	0.03 [-0.12, 0.19]	0.67	
Hannum	6	-0.02 [-0.20, 0.16]	0.86	
Horvath Skin & Blood	2	0.18 [-0.18, 0.54]	0.33	
Zhang	3	-0.55 [-1.06, -0.07]	0.02	*
2nd Generation	12	0.32 [0.16, 0.48]	0	*
PhenoAge	5	0.24 [0.06, 0.43]	0.01	*
GrimAge	7	0.42 [0.25, 0.60]	0	*
3rd Generation	6	0.30 [0.09, 0.51]	0	*
DunedinPoAm	3	0.42 [0.17, 0.67]	0	*
DunedinPACE	3	0.23 [-0.01, 0.46]	0.06	

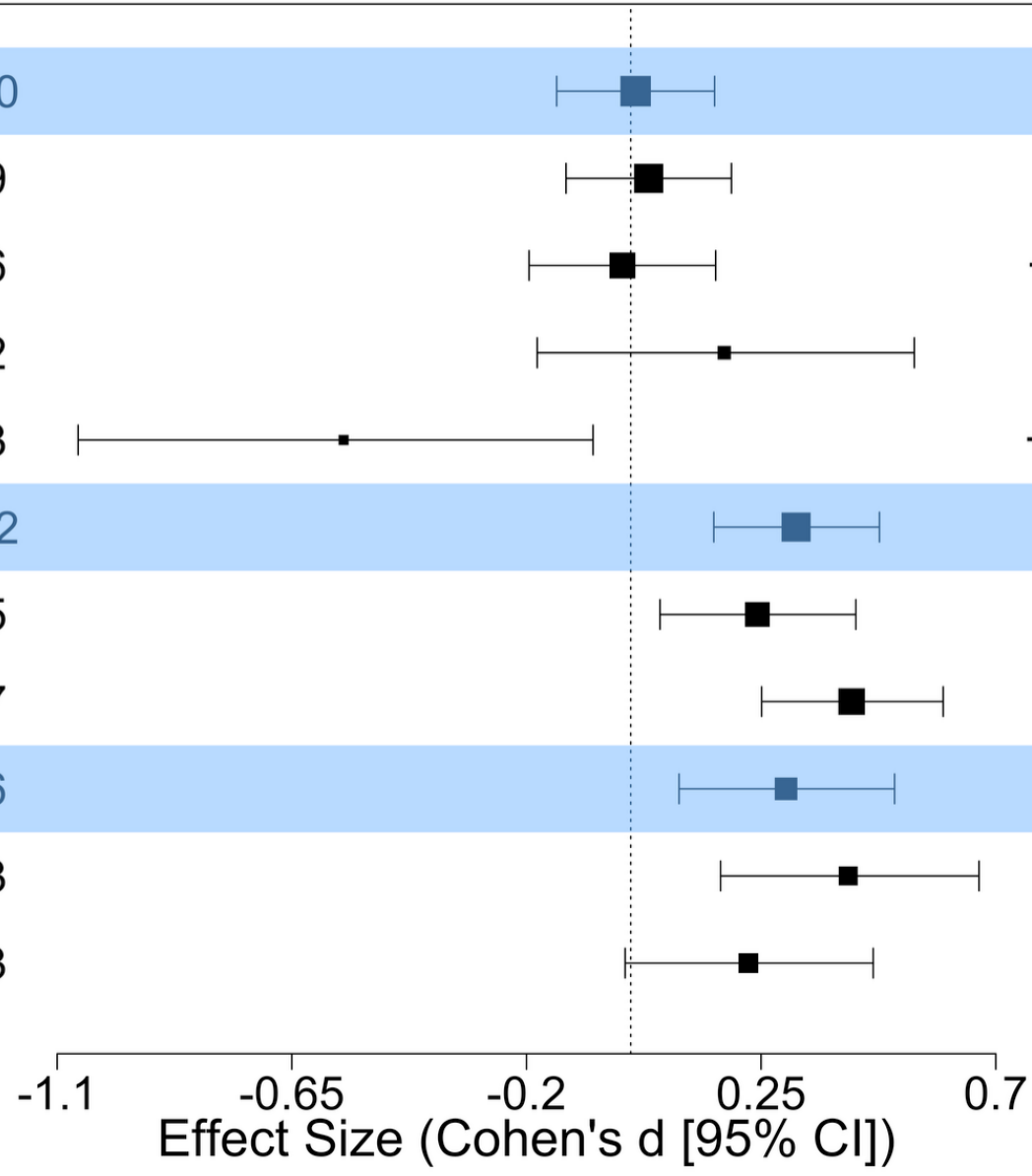

B. Meta-analytic associations between Latinx-White comparisons and epigenetic clocks (excluding effect sizes from the Health and Retirement Study)

DNAm Measure	Nr. of Effect Sizes	Estimate [95% CI]	p-value	
1st Generation	8	0.11 [-0.07, 0.29]	0.23	
Horvath Pan-Tissue	4	-0.11 [-0.30, 0.09]	0.29	
Hannum	2	0.31 [0.11, 0.52]	0	*
Horvath Skin & Blood	2	0.20 [-0.10, 0.51]	0.2	
2nd Generation	5	0.12 [-0.08, 0.32]	0.25	
PhenoAge	3	0.18 [-0.01, 0.38]	0.07	
GrimAge	2	0.16 [-0.10, 0.42]	0.22	
3rd Generation	5	0.32 [0.10, 0.55]	0	*
DunedinPoAm	4	0.36 [0.14, 0.58]	0	*
DunedinPACE	1	0.31 [-0.01, 0.63]	0.05	

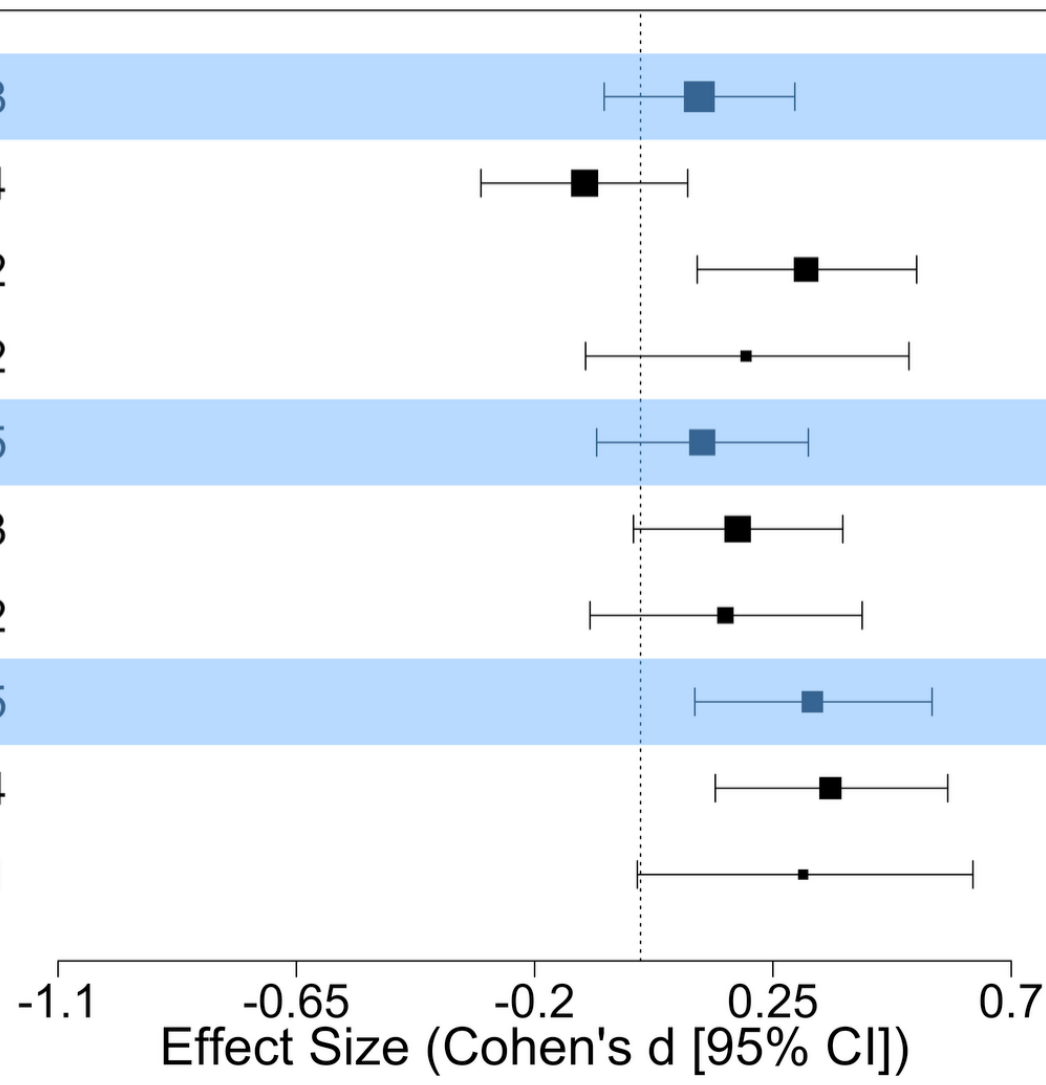

Supplemental Figure 13. Meta-analytic associations between race/ethnicity and epigenetic clocks, excluding effect sizes from the Health and Retirement Study. Forest plot depicts the meta-analytic associations between (A) Black-White comparisons and generations of epigenetic clocks and (B) Latinx-White comparisons and generations of epigenetic clocks, after 30 and 23 effect sizes contributed by the Health and Retirement Study are removed, respectively. Blue panels indicate the meta-analyzed effect size estimates for first-, second-, and third-generation clocks, followed by individual clocks within each category. Analyses were conducted using two-sided Fisher-Z transformation and later transformed to Cohen's d to ease interpretation. Effect size estimates are bounded by 95% confidence intervals (CIs).* denotes significant difference from 0 at $p < 0.05$ (not corrected for multiple comparisons). The size of the boxes in the middle panel represents the precision of the effect size estimate (i.e., the smaller the CI, the more precise the estimate).

A. Meta-analytic associations between socioeconomic status (SES) and epigenetic clocks (EPIC array)

DNAm Measure	Nr. of Effect Sizes	Estimate [95% CI]	p-value	
1st Generation	218	-0.02 [-0.04, -0.00]	0.04	*
Horvath Pan-Tissue	96	-0.01 [-0.04, 0.01]	0.27	
Hannum	93	-0.03 [-0.05, -0.00]	0.04	*
Horvath Skin & Blood	12	-0.05 [-0.11, 0.00]	0.06	
Zhang	10	-0.02 [-0.11, 0.06]	0.58	
PedBE	7	-0.01 [-0.10, 0.08]	0.83	
2nd Generation	230	-0.11 [-0.13, -0.09]	0	*
PhenoAge	104	-0.07 [-0.10, -0.05]	0	*
GrimAge	126	-0.15 [-0.17, -0.13]	0	*
3rd Generation	101	-0.14 [-0.16, -0.11]	0	*
DunedinPoAm	75	-0.14 [-0.17, -0.11]	0	*
DunedinPACE	26	-0.14 [-0.18, -0.10]	0	*

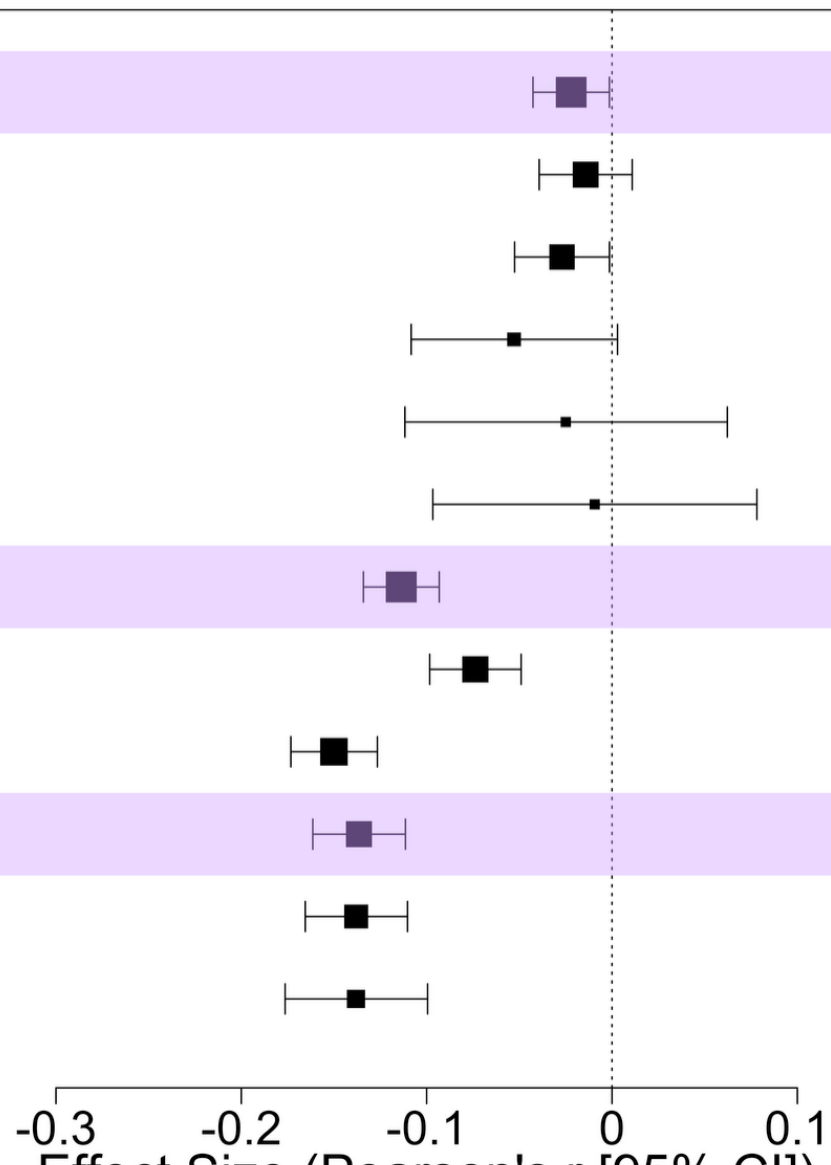

B. Meta-analytic associations between SES and epigenetic clocks (450k array)

DNAm Measure	Nr. of Effect Sizes	Estimate [95% CI]	p-value	
1st Generation	171	-0.04 [-0.07, -0.02]	0	*
Horvath Pan-Tissue	79	-0.03 [-0.06, 0.00]	0.06	
Hannum	70	-0.05 [-0.08, -0.02]	0	*
Horvath Skin & Blood	7	-0.04 [-0.12, 0.03]	0.25	
Zhang	12	-0.06 [-0.12, 0.01]	0.07	
PedBE	3	-0.06 [-0.21, 0.10]	0.47	
2nd Generation	86	-0.12 [-0.15, -0.09]	0	*
PhenoAge	36	-0.08 [-0.12, -0.04]	0	*
GrimAge	50	-0.15 [-0.19, -0.11]	0	*
3rd Generation	40	-0.16 [-0.20, -0.12]	0	*
DunedinPoAm	34	-0.14 [-0.18, -0.10]	0	*
DunedinPACE	6	-0.22 [-0.29, -0.14]	0	*

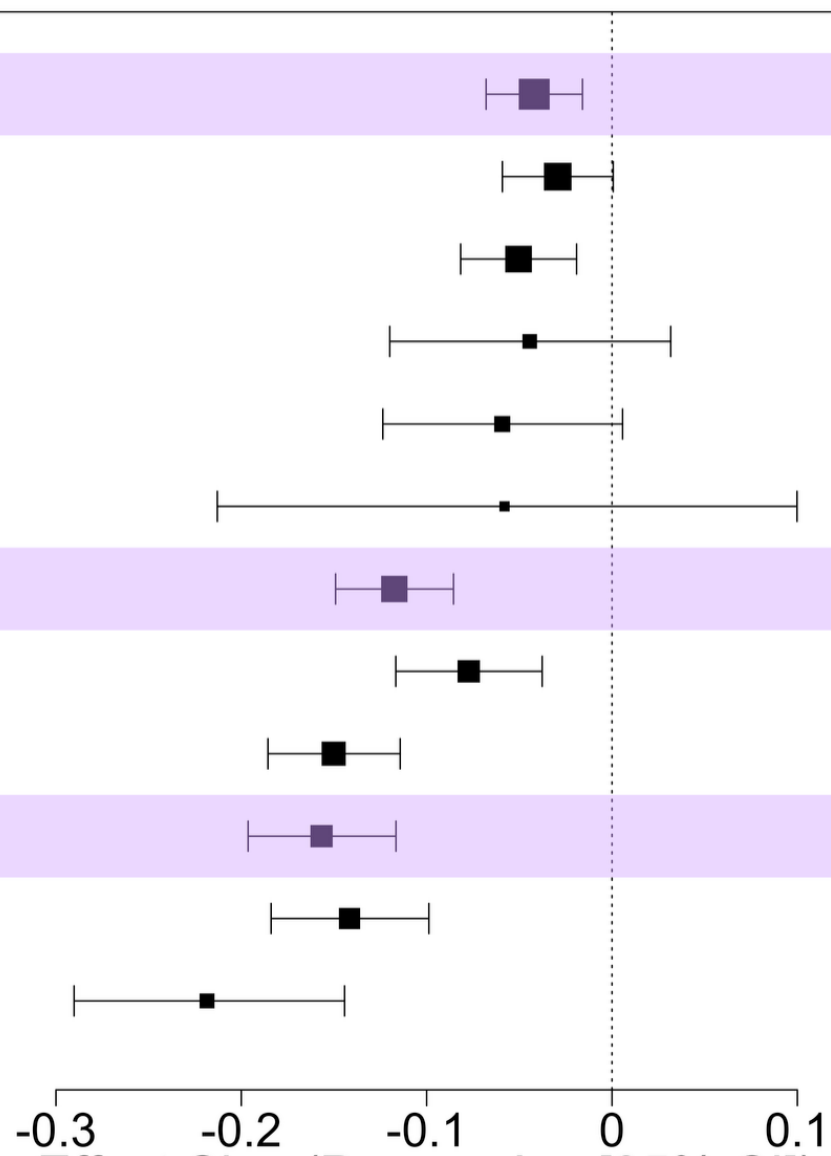

Supplemental Figure 14. Meta-analytic associations between socioeconomic status (SES) and epigenetic clocks, separately for EPIC and 450k arrays. Forest plot depicts the meta-analytic associations of SES and epigenetic clocks for EPIC array (section A) and 450k array (section B). Purple panels indicate the meta-analyzed effect size estimates for first-, second-, and third-generation clocks, followed by individual clocks within each category. Analyses were conducted using two-sided Fisher-Z transformation and later transformed to Cohen's d to ease interpretation. Effect size estimates are bounded by 95% confidence intervals (CIs).* denotes significant difference from 0 at $p < 0.05$ (not corrected for multiple comparisons). The size of the boxes in the middle panel represents the precision of the effect size estimate (i.e., the smaller the CI, the more precise the estimate).

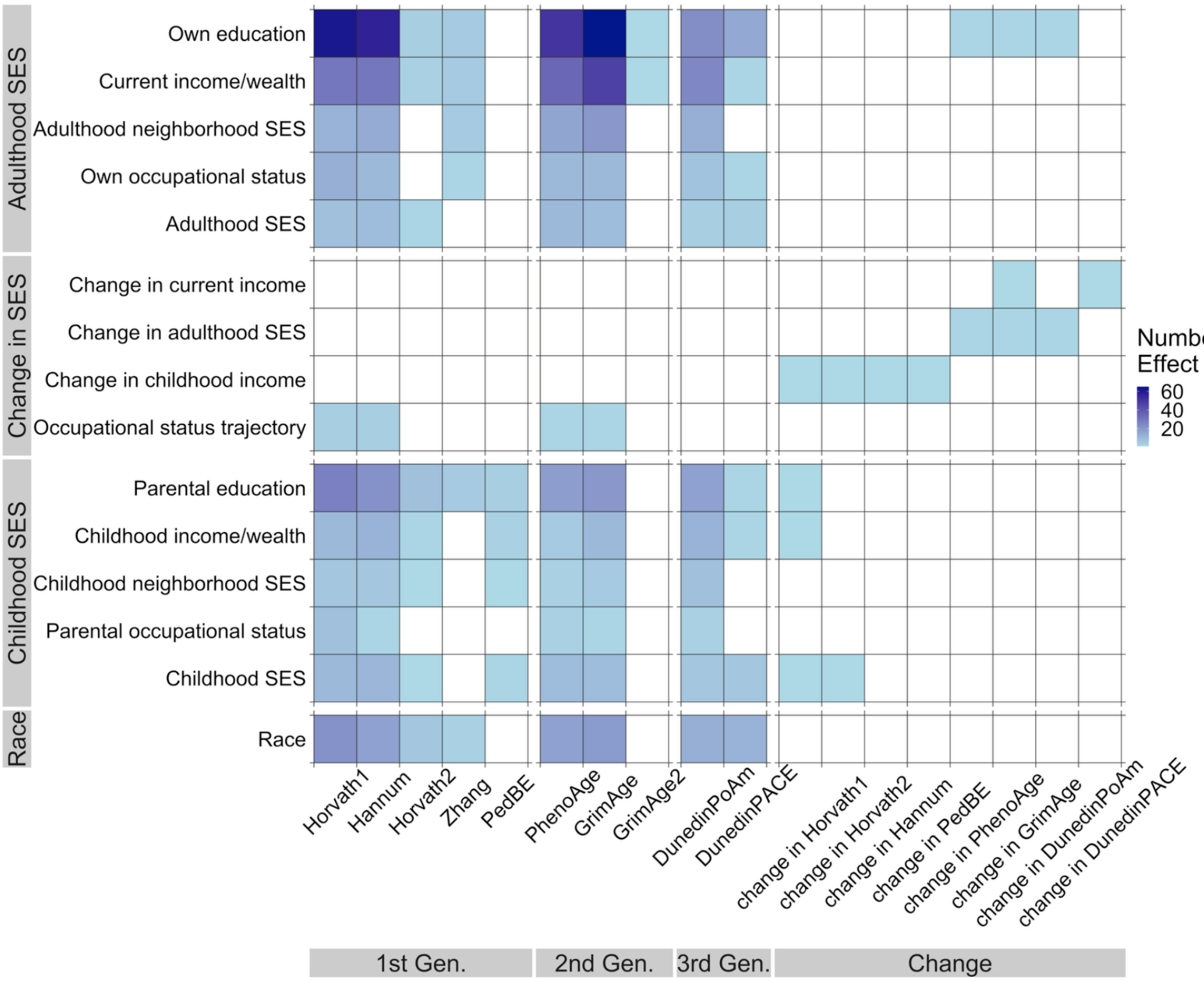

Supplemental Figure 15. Heatmap showing the number of effect sizes for epigenetic-clock-measured biological aging (x-axis) and social determinants of health (y-axis). The number of effect sizes per category ranges from 60 (dark blue) to 1 (light blue). White squares indicate no effect sizes.

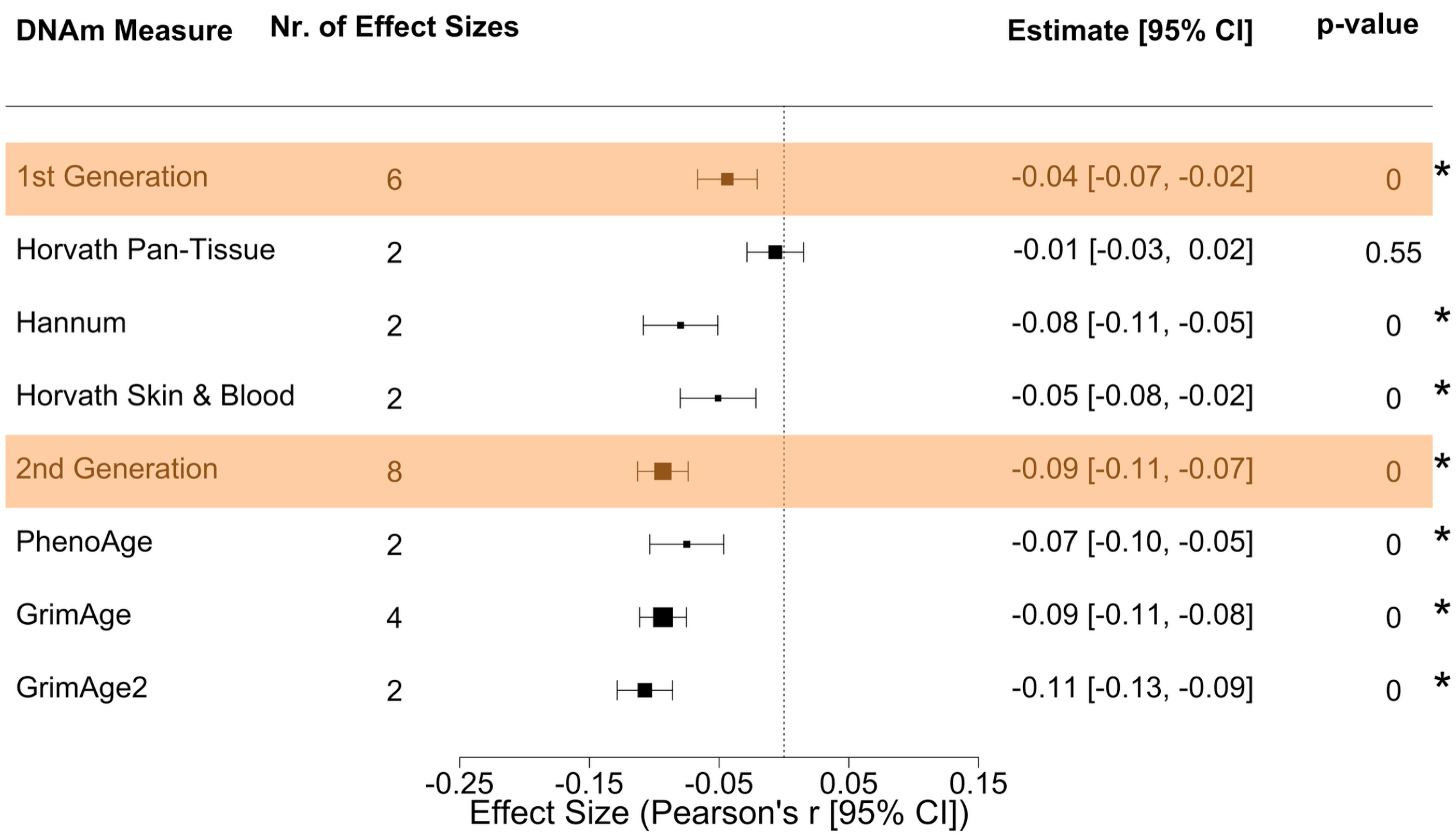

Supplemental Figure 16. Meta-analytic associations between socioeconomic status (SES) and epigenetic clocks – biweight midcorrelation (bicor) estimates. Forest plot depicts the meta-analytic associations between SES and epigenetic clocks. As a reliable conversion from bicor to Pearson's r could not be found, bicor effect sizes are meta-analyzed separately using two-sided hypothesis testing. Orange panels indicate the meta-analyzed effect size estimates for first- and second-generation clocks, followed by individual clocks within each category. Effect size estimates are bounded by 95% confidence intervals (CIs). Effect size estimates are bounded by 95% confidence intervals (CIs).* denotes significant difference from 0 at $p < 0.05$ (not corrected for multiple comparisons). The size of the boxes in the middle panel represents the precision of the effect size estimate (i.e., the smaller the CI, the more precise the estimate).

A. Meta-analytic associations between SES and epigenetic clocks (excluding effect sizes from Avon Longitudinal Study of Parents and Children)

DNAm Measure	Nr. of Effect Sizes	Estimate [95% CI]	p-value	
1st Generation	397	-0.03 [-0.04, -0.01]	0	*
Horvath Pan-Tissue	178	-0.01 [-0.03, 0.00]	0.14	
Hannum	168	-0.03 [-0.05, -0.02]	0	*
Horvath Skin & Blood	19	-0.05 [-0.09, -0.00]	0.04	*
Zhang	22	-0.04 [-0.09, 0.01]	0.11	
PedBE	10	-0.02 [-0.09, 0.05]	0.58	
2nd Generation	351	-0.11 [-0.12, -0.09]	0	*
PhenoAge	158	-0.07 [-0.09, -0.05]	0	*
GrimAge	193	-0.14 [-0.16, -0.12]	0	*
3rd Generation	139	-0.14 [-0.16, -0.12]	0	*
DunedinPoAm	106	-0.14 [-0.16, -0.11]	0	*
DunedinPACE	33	-0.15 [-0.18, -0.12]	0	*

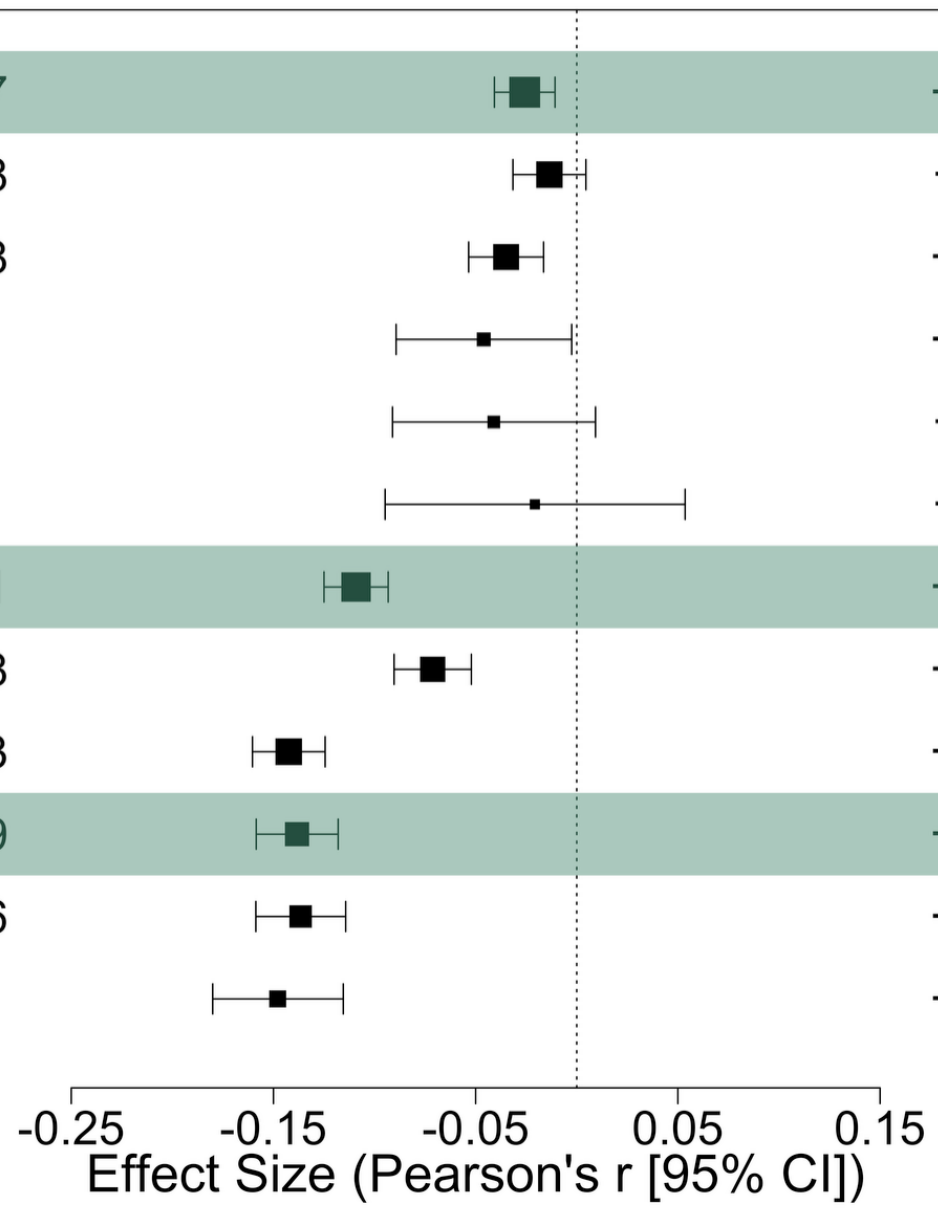

B. Meta-analytic associations between SES and epigenetic clocks (excluding effect sizes from Birmingham Youth Violence Study)

DNAm Measure	Nr. of Effect Sizes	Estimate [95% CI]	p-value	
1st Generation	396	-0.03 [-0.04, -0.01]	0	*
Horvath Pan-Tissue	179	-0.01 [-0.03, 0.01]	0.16	
Hannum	166	-0.04 [-0.05, -0.02]	0	*
Horvath Skin & Blood	19	-0.04 [-0.09, -0.00]	0.05	*
Zhang	22	-0.04 [-0.09, 0.01]	0.13	
PedBE	10	-0.02 [-0.09, 0.06]	0.6	
2nd Generation	341	-0.11 [-0.12, -0.09]	0	*
PhenoAge	153	-0.07 [-0.09, -0.05]	0	*
GrimAge	188	-0.14 [-0.16, -0.12]	0	*
3rd Generation	151	-0.13 [-0.15, -0.11]	0	*
DunedinPoAm	118	-0.13 [-0.15, -0.11]	0	*
DunedinPACE	33	-0.15 [-0.18, -0.11]	0	*

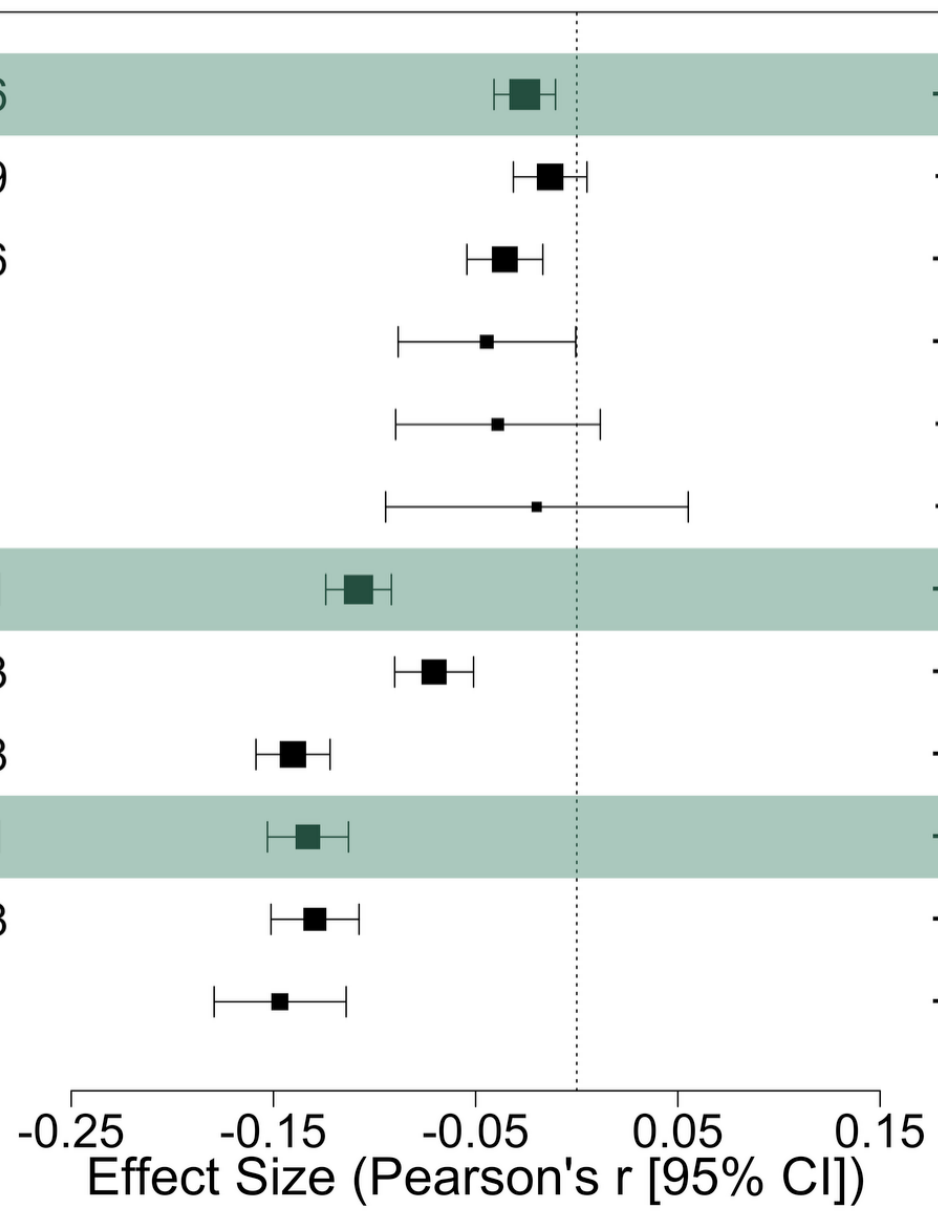

C. Meta-analytic associations between SES and epigenetic clocks (excluding effect sizes from Canadian Longitudinal Study on Aging)

DNAm Measure	Nr. of Effect Sizes	Estimate [95% CI]	p-value	
1st Generation	398	-0.03 [-0.04, -0.01]	0	*
Horvath Pan-Tissue	180	-0.02 [-0.03, 0.00]	0.1	
Hannum	167	-0.03 [-0.05, -0.01]	0	*
Horvath Skin & Blood	19	-0.04 [-0.09, -0.00]	0.05	*
Zhang	22	-0.04 [-0.09, 0.01]	0.13	
PedBE	10	-0.02 [-0.09, 0.05]	0.6	
2nd Generation	341	-0.11 [-0.12, -0.09]	0	*
PhenoAge	153	-0.07 [-0.09, -0.05]	0	*
GrimAge	188	-0.14 [-0.16, -0.12]	0	*
3rd Generation	149	-0.13 [-0.15, -0.11]	0	*
DunedinPoAm	116	-0.13 [-0.15, -0.11]	0	*
DunedinPACE	33	-0.15 [-0.18, -0.11]	0	*

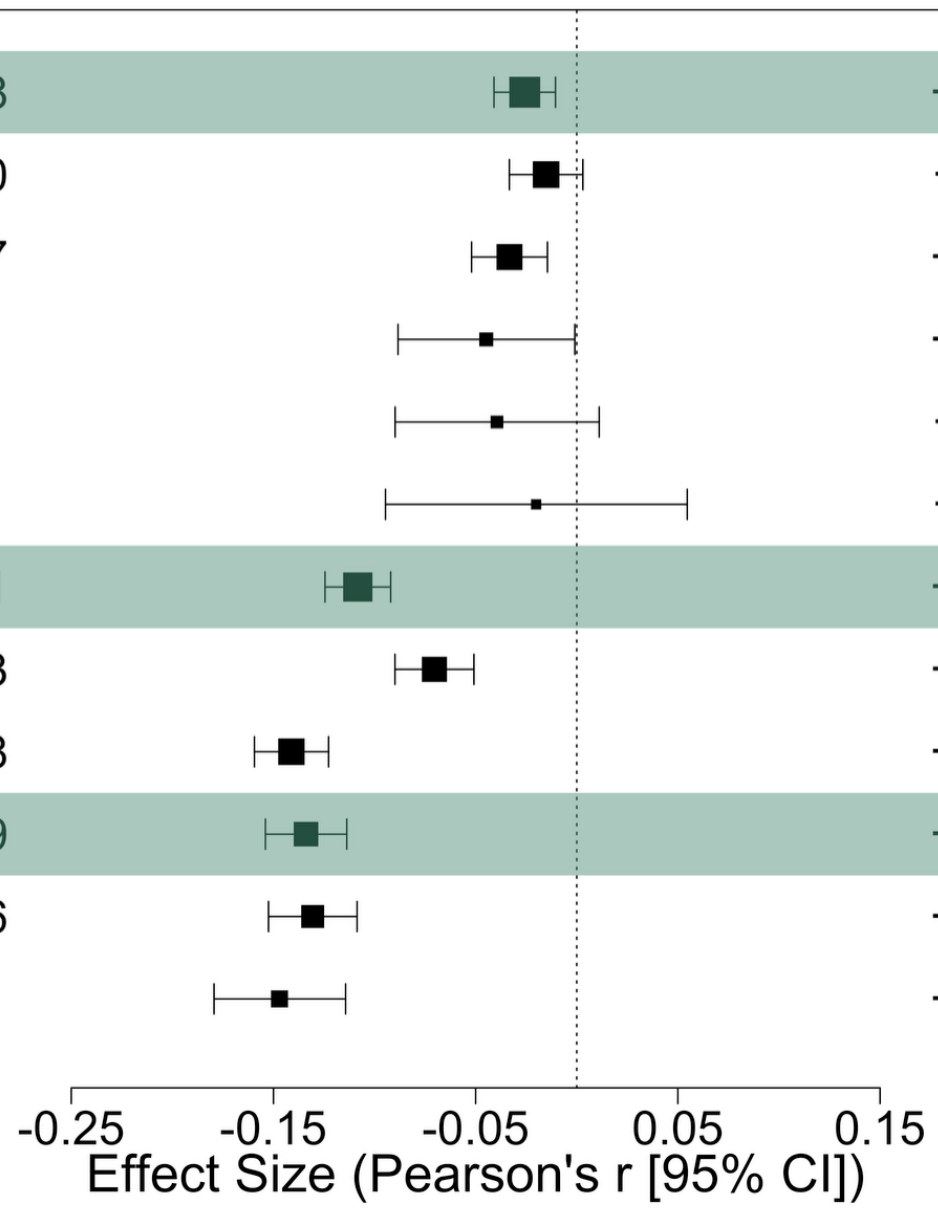

D. Meta-analytic associations between SES and epigenetic clocks (excluding effect sizes from Coronary Artery Risk Development in Young Adults)

DNAm Measure	Nr. of Effect Sizes	Estimate [95% CI]	p-value	
1st Generation	394	-0.02 [-0.04, -0.01]	0	*
Horvath Pan-Tissue	178	-0.01 [-0.03, 0.00]	0.11	
Hannum	165	-0.03 [-0.05, -0.01]	0	*
Horvath Skin & Blood	19	-0.05 [-0.09, -0.00]	0.04	*
Zhang	22	-0.04 [-0.09, 0.01]	0.12	
PedBE	10	-0.02 [-0.10, 0.05]	0.59	
2nd Generation	337	-0.11 [-0.13, -0.09]	0	*
PhenoAge	151	-0.07 [-0.09, -0.05]	0	*
GrimAge	186	-0.14 [-0.16, -0.13]	0	*
3rd Generation	145	-0.14 [-0.16, -0.12]	0	*
DunedinPoAm	112	-0.13 [-0.16, -0.11]	0	*
DunedinPACE	33	-0.15 [-0.18, -0.11]	0	*

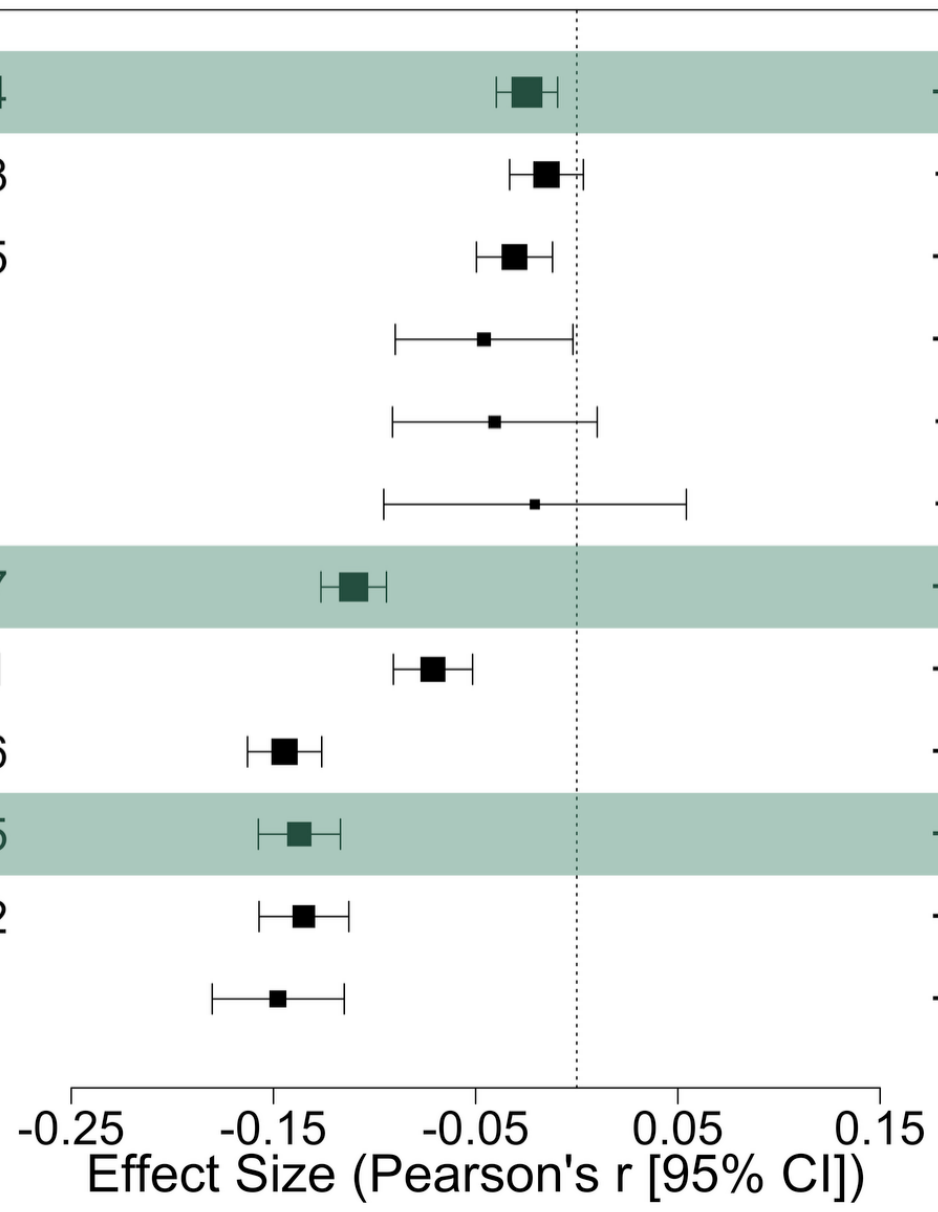

E. Meta-analytic associations between SES and epigenetic clocks (excluding effect sizes from E-Risk)

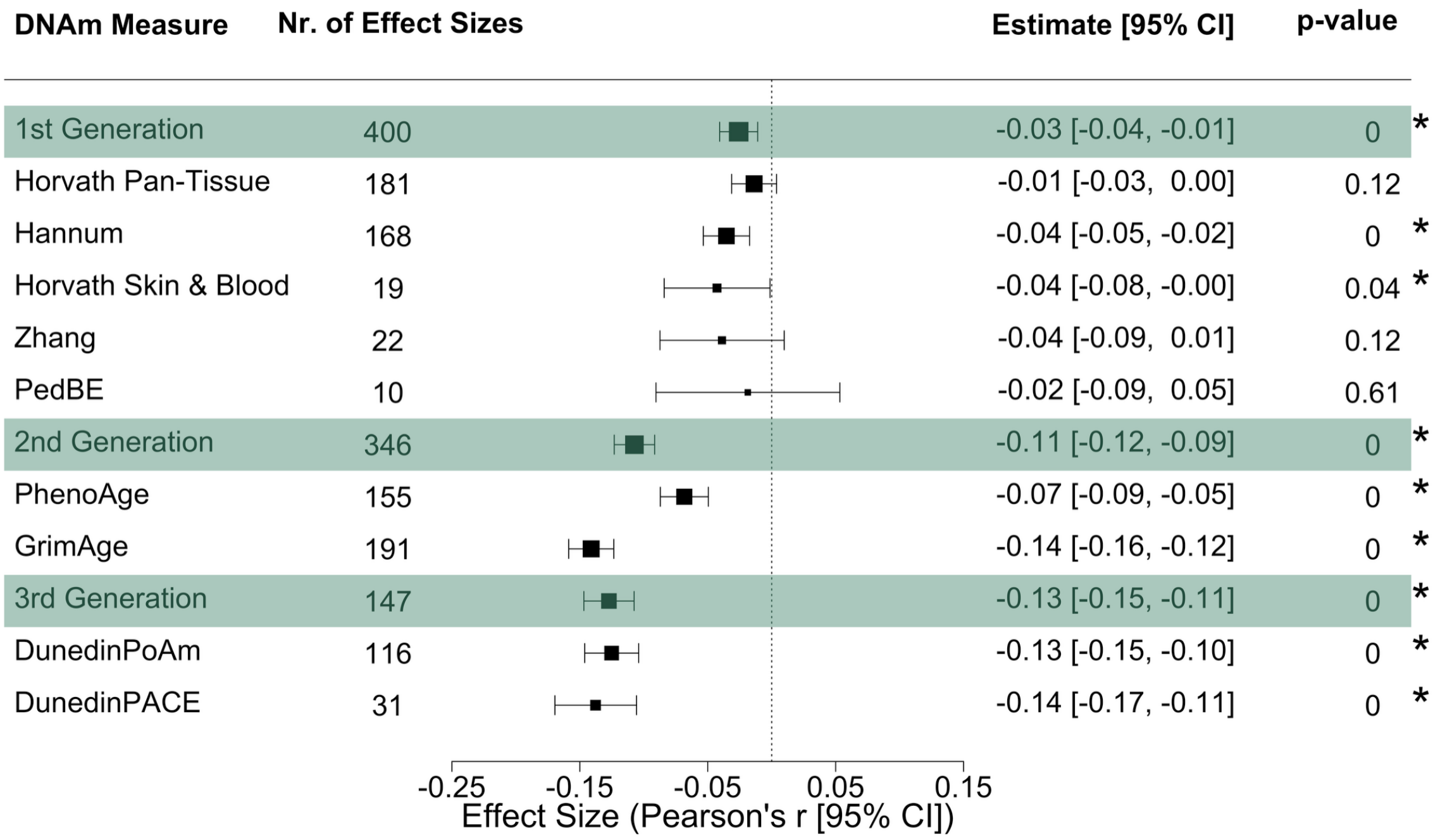

F. Meta-analytic associations between SES and epigenetic clocks (excluding effect sizes from Family and Community Health Study)

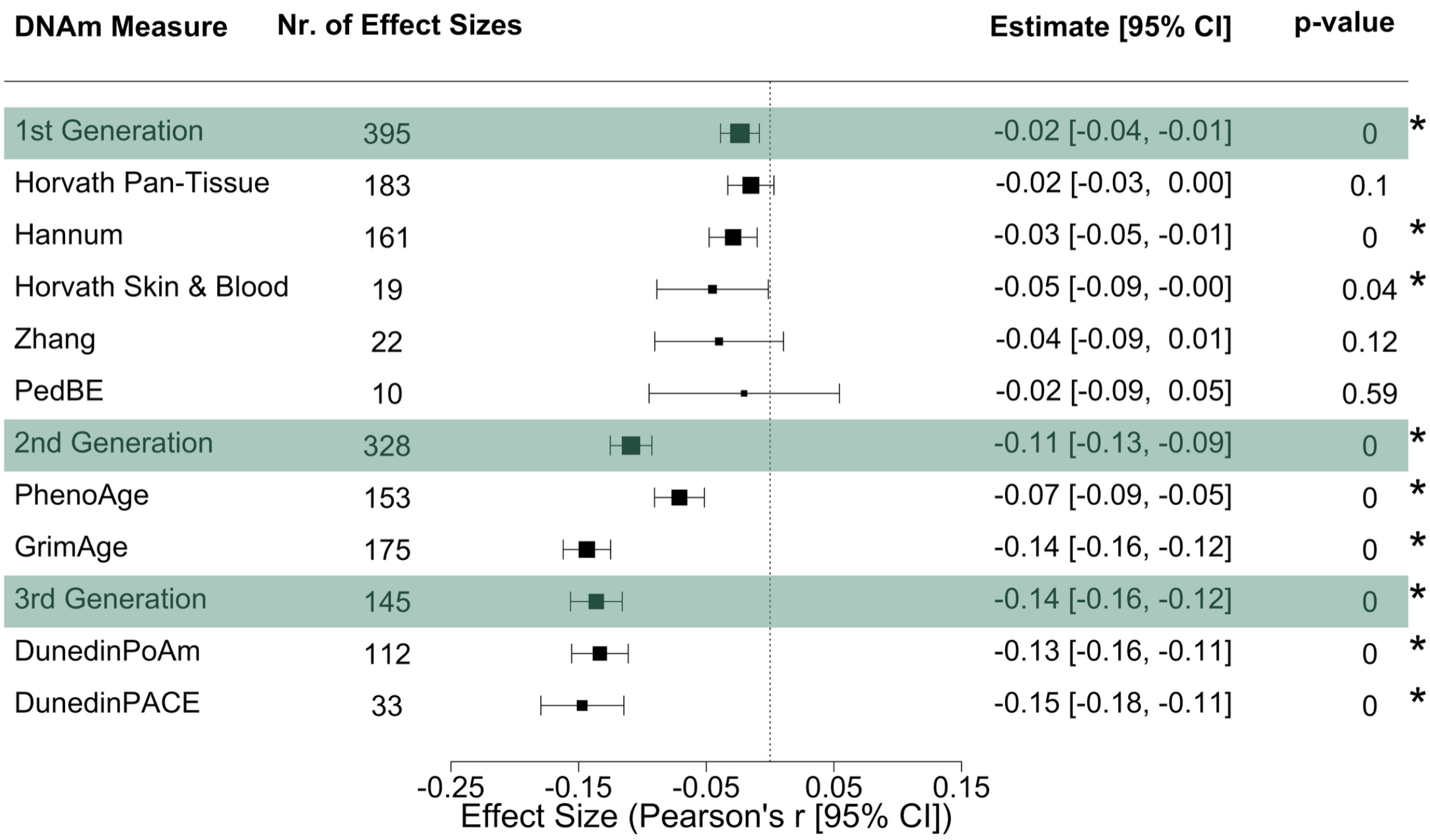

G. Meta-analytic associations between SES and epigenetic clocks (excluding effect sizes from Female Growth and Development Study)

DNAm Measure	Nr. of Effect Sizes	Estimate [95% CI]	p-value	
1st Generation	400	-0.02 [-0.04, -0.01]	0	*
Horvath Pan-Tissue	181	-0.01 [-0.03, 0.00]	0.16	
Hannum	168	-0.03 [-0.05, -0.02]	0	*
Horvath Skin & Blood	19	-0.04 [-0.08, 0.00]	0.06	
Zhang	22	-0.03 [-0.08, 0.02]	0.17	
PedBE	10	-0.02 [-0.09, 0.05]	0.61	
2nd Generation	345	-0.10 [-0.12, -0.09]	0	*
PhenoAge	155	-0.07 [-0.08, -0.05]	0	*
GrimAge	190	-0.14 [-0.15, -0.12]	0	*
3rd Generation	148	-0.13 [-0.15, -0.11]	0	*
DunedinPoAm	115	-0.12 [-0.15, -0.10]	0	*
DunedinPACE	33	-0.14 [-0.18, -0.11]	0	*

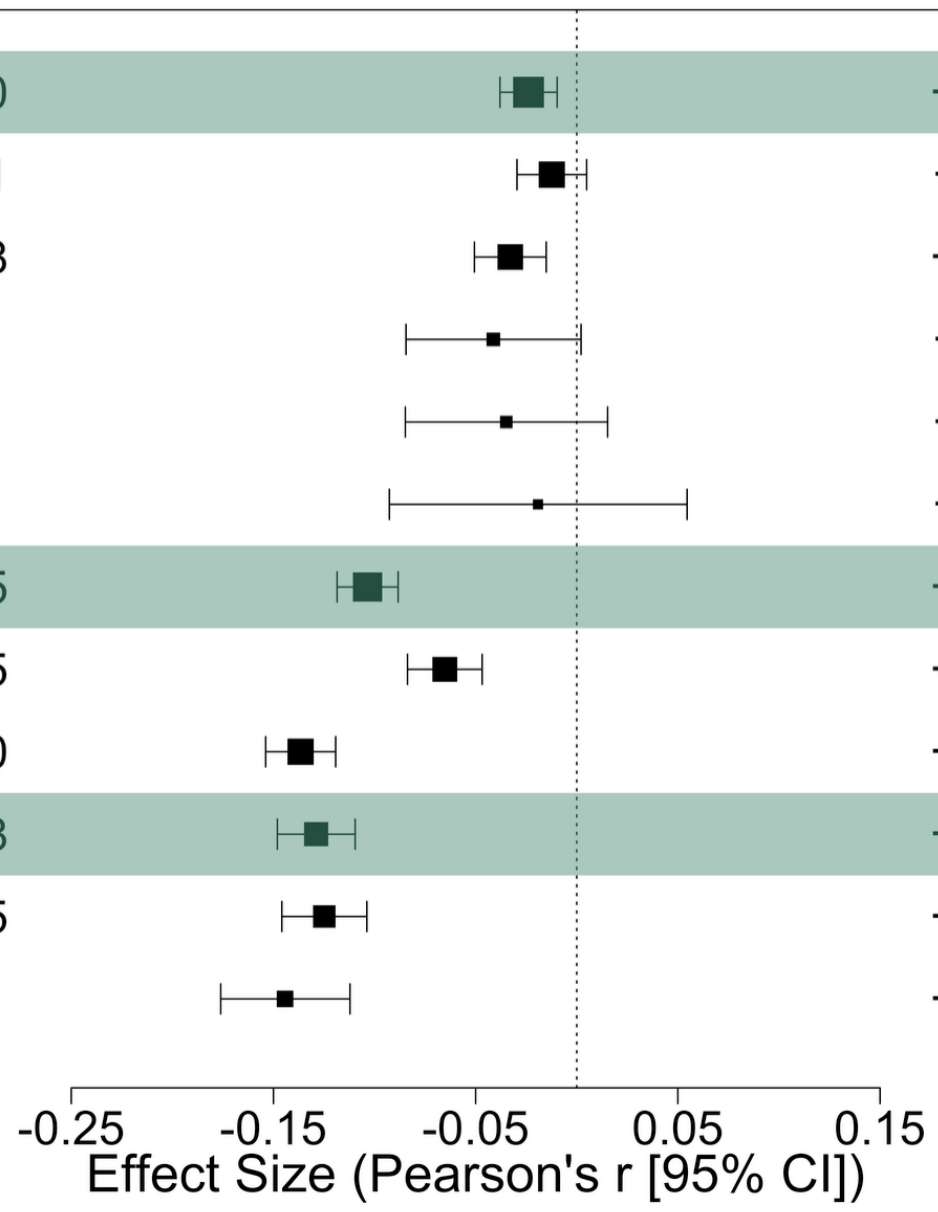

H. Meta-analytic associations between SES and epigenetic clocks (excluding effect sizes from Finnish Twin Cohort)

DNAm Measure	Nr. of Effect Sizes	Estimate [95% CI]	p-value	
1st Generation	402	-0.03 [-0.04, -0.01]	0	*
Horvath Pan-Tissue	181	-0.02 [-0.03, 0.00]	0.09	
Hannum	170	-0.03 [-0.05, -0.02]	0	*
Horvath Skin & Blood	19	-0.05 [-0.09, -0.00]	0.04	*
Zhang	22	-0.04 [-0.09, 0.01]	0.12	
PedBE	10	-0.02 [-0.09, 0.05]	0.6	
2nd Generation	346	-0.11 [-0.12, -0.09]	0	*
PhenoAge	156	-0.07 [-0.09, -0.05]	0	*
GrimAge	190	-0.14 [-0.16, -0.12]	0	*
3rd Generation	151	-0.13 [-0.15, -0.11]	0	*
DunedinPoAm	118	-0.13 [-0.15, -0.11]	0	*
DunedinPACE	33	-0.15 [-0.18, -0.11]	0	*

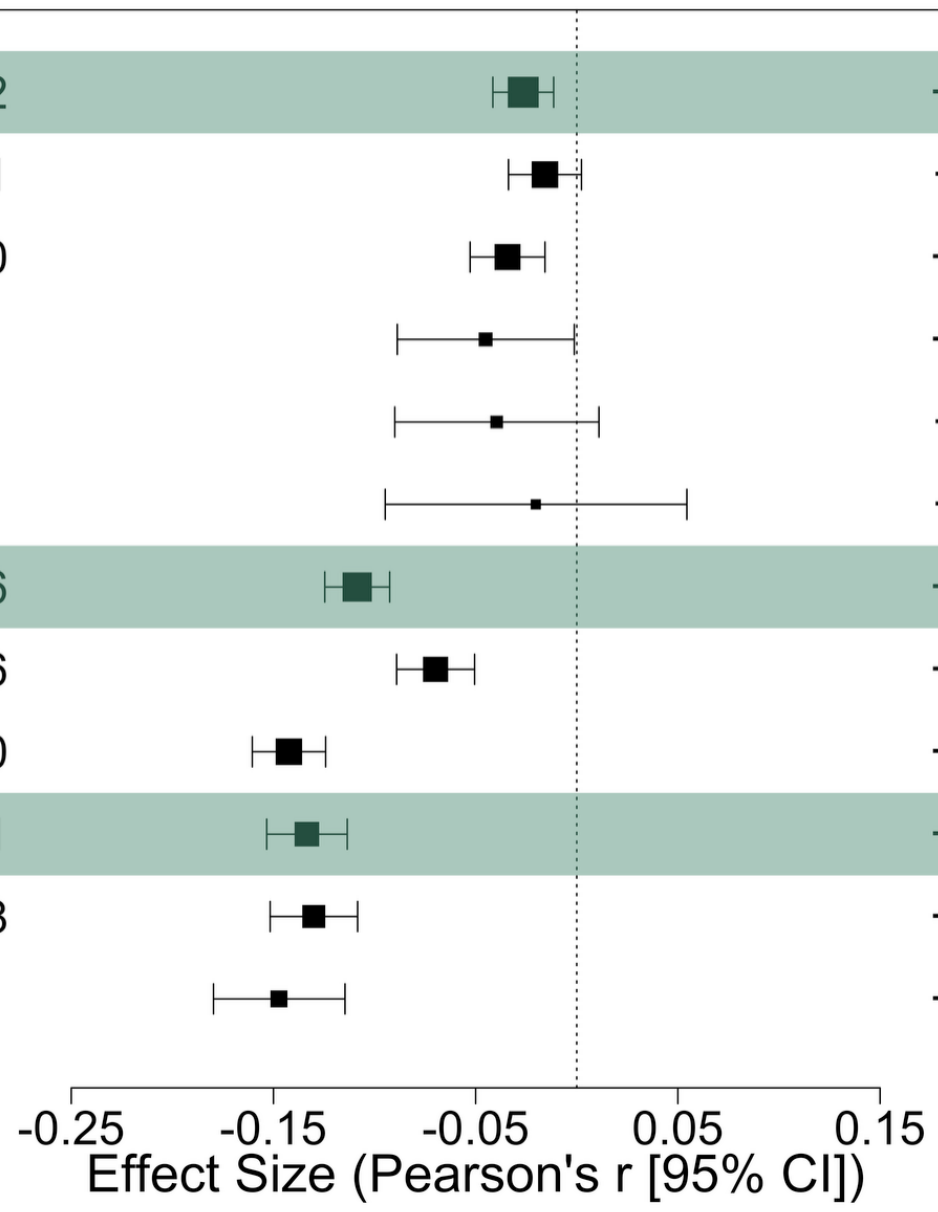

I. Meta-analytic associations between SES and epigenetic clocks (excluding effect sizes from Generation Scotland)

DNAm Measure	Nr. of Effect Sizes	Estimate [95% CI]	p-value	
1st Generation	398	-0.03 [-0.04, -0.01]	0	*
Horvath Pan-Tissue	180	-0.02 [-0.03, 0.00]	0.07	
Hannum	167	-0.03 [-0.05, -0.01]	0	*
Horvath Skin & Blood	19	-0.04 [-0.09, -0.00]	0.05	*
Zhang	22	-0.04 [-0.09, 0.01]	0.13	
PedBE	10	-0.02 [-0.09, 0.05]	0.6	
2nd Generation	347	-0.11 [-0.12, -0.09]	0	*
PhenoAge	156	-0.07 [-0.09, -0.05]	0	*
GrimAge	191	-0.14 [-0.16, -0.12]	0	*
3rd Generation	149	-0.13 [-0.15, -0.11]	0	*
DunedinPoAm	118	-0.13 [-0.15, -0.11]	0	*
DunedinPACE	31	-0.14 [-0.17, -0.11]	0	*

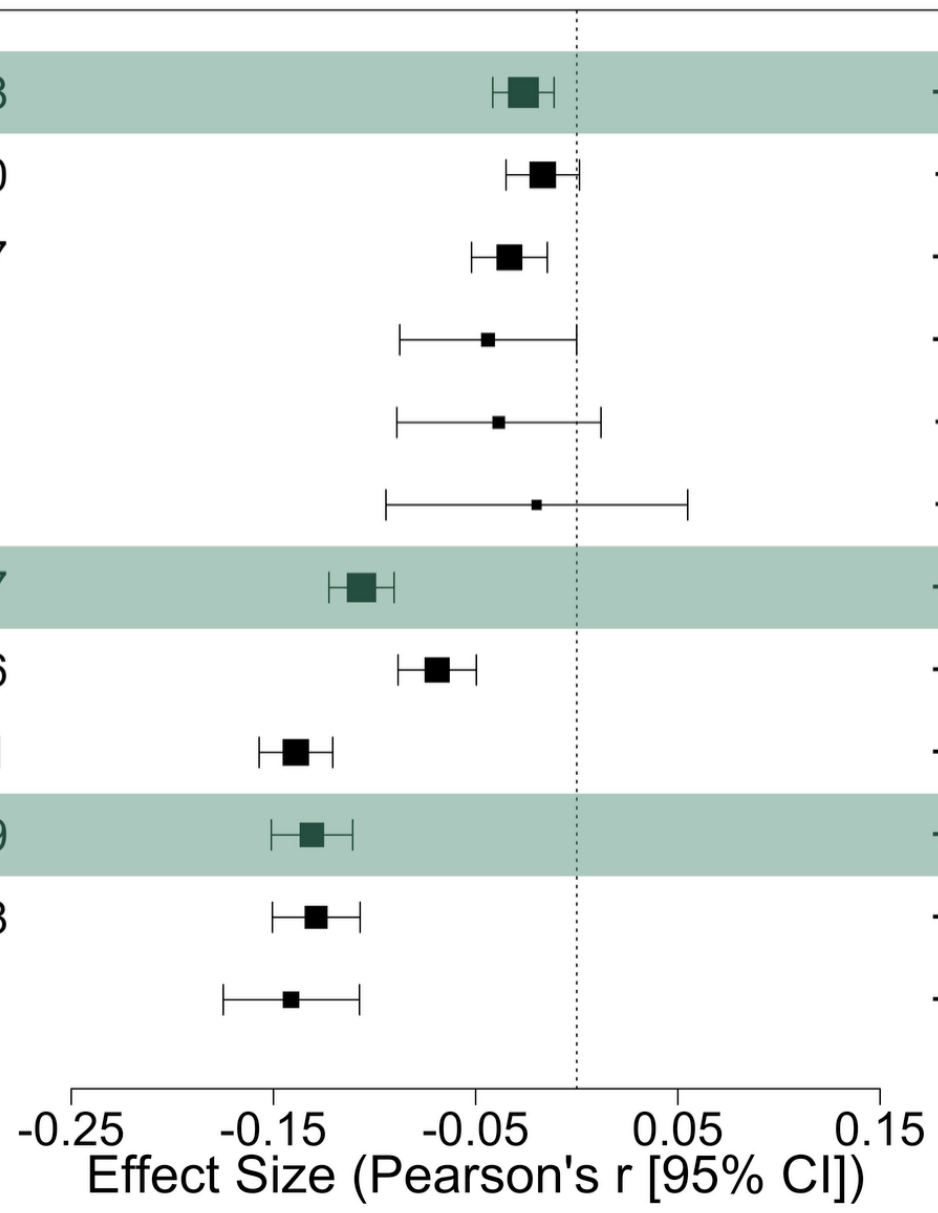

J. Meta-analytic associations between SES and epigenetic clocks (excluding effect sizes from Lothian Birth Cohort 1936)

DNAm Measure	Nr. of Effect Sizes	Estimate [95% CI]	p-value	
1st Generation	402	-0.03 [-0.04, -0.01]	0	*
Horvath Pan-Tissue	182	-0.01 [-0.03, 0.00]	0.11	
Hannum	169	-0.03 [-0.05, -0.02]	0	*
Horvath Skin & Blood	19	-0.04 [-0.09, -0.00]	0.05	*
Zhang	22	-0.04 [-0.09, 0.01]	0.13	
PedBE	10	-0.02 [-0.09, 0.05]	0.6	
2nd Generation	348	-0.11 [-0.12, -0.09]	0	*
PhenoAge	156	-0.07 [-0.09, -0.05]	0	*
GrimAge	192	-0.14 [-0.16, -0.12]	0	*
3rd Generation	151	-0.13 [-0.15, -0.11]	0	*
DunedinPoAm	118	-0.13 [-0.15, -0.11]	0	*
DunedinPACE	33	-0.15 [-0.18, -0.11]	0	*

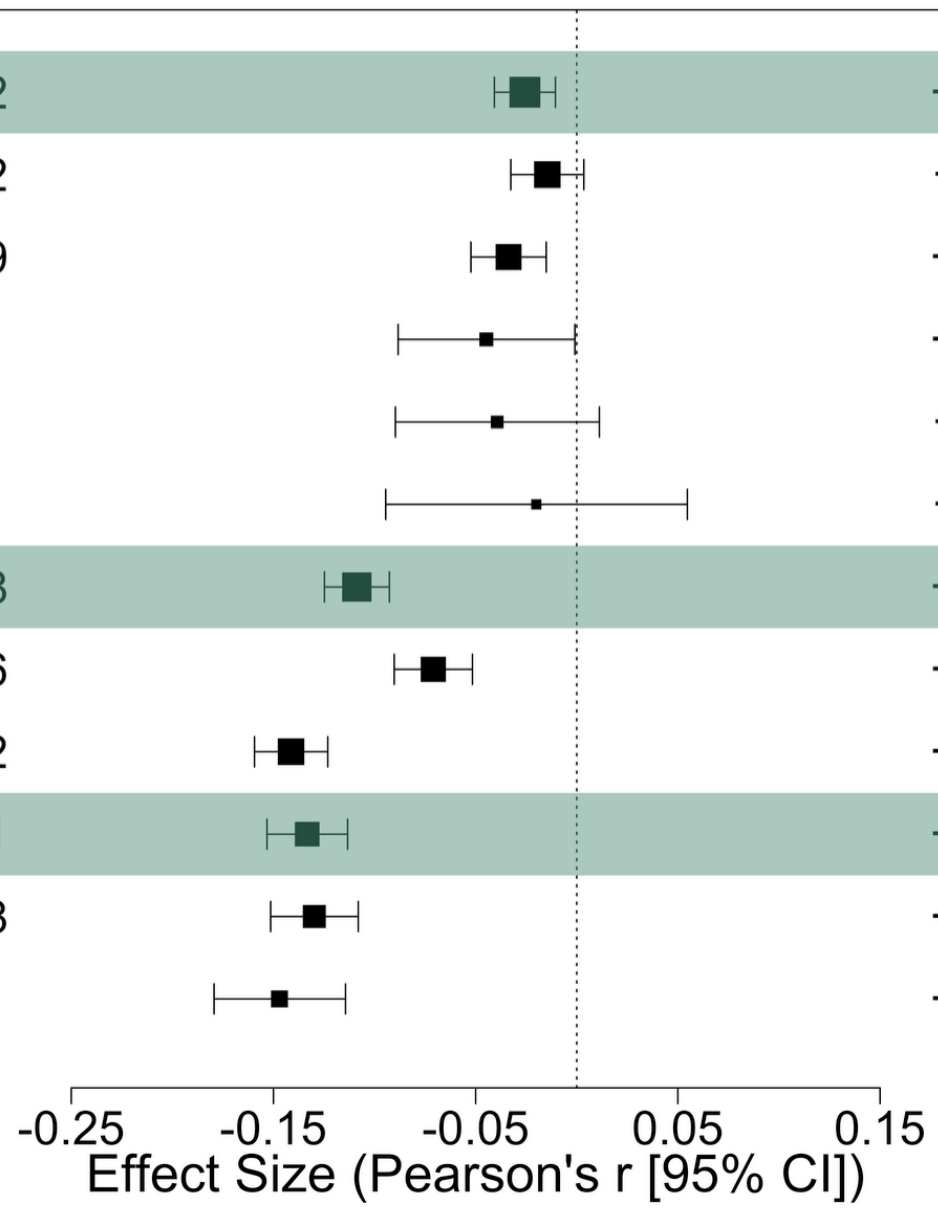

K. Meta-analytic associations between SES and epigenetic clocks (excluding effect sizes from Multi-Ethnic Study of Atherosclerosis)

DNAm Measure	Nr. of Effect Sizes	Estimate [95% CI]	p-value	
1st Generation	359	-0.03 [-0.04, -0.01]	0	*
Horvath Pan-Tissue	168	-0.02 [-0.03, 0.00]	0.09	
Hannum	155	-0.03 [-0.05, -0.01]	0	*
Horvath Skin & Blood	16	-0.06 [-0.11, -0.01]	0.02	*
Zhang	10	-0.02 [-0.11, 0.06]	0.62	
PedBE	10	-0.02 [-0.10, 0.06]	0.6	
2nd Generation	311	-0.11 [-0.13, -0.09]	0	*
PhenoAge	142	-0.07 [-0.09, -0.05]	0	*
GrimAge	169	-0.14 [-0.16, -0.12]	0	*
3rd Generation	131	-0.13 [-0.15, -0.11]	0	*
DunedinPoAm	98	-0.13 [-0.15, -0.11]	0	*
DunedinPACE	33	-0.15 [-0.18, -0.11]	0	*

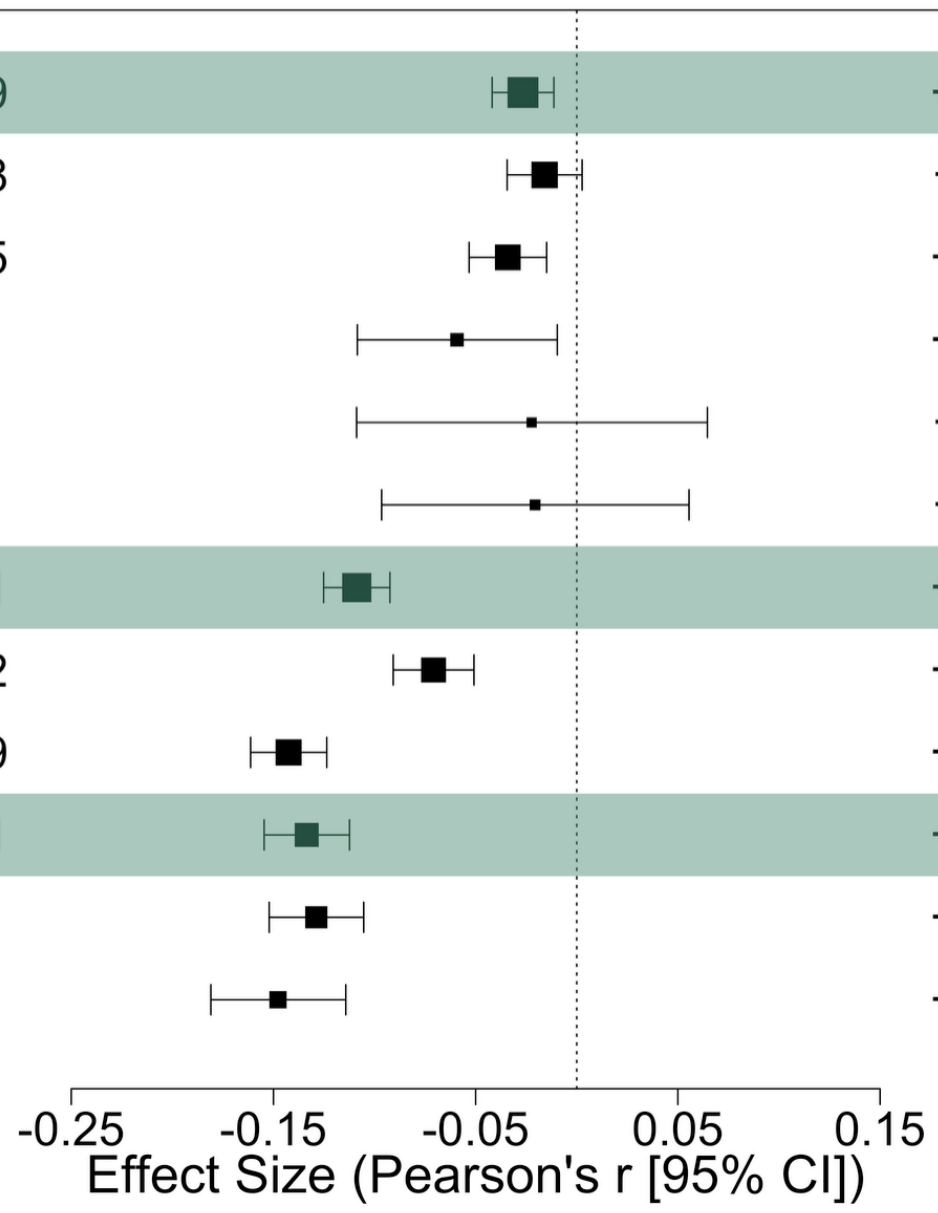

L. Meta-analytic associations between SES and epigenetic clocks (excluding effect sizes from MRC National Survey of Health and Development)

DNAm Measure	Nr. of Effect Sizes	Estimate [95% CI]	p-value	
1st Generation	391	-0.03 [-0.04, -0.01]	0	*
Horvath Pan-Tissue	173	-0.02 [-0.03, 0.00]	0.1	
Hannum	167	-0.03 [-0.05, -0.02]	0	*
Horvath Skin & Blood	19	-0.04 [-0.09, -0.00]	0.05	*
Zhang	22	-0.04 [-0.09, 0.01]	0.14	
PedBE	10	-0.02 [-0.09, 0.05]	0.6	
2nd Generation	343	-0.11 [-0.12, -0.09]	0	*
PhenoAge	154	-0.07 [-0.09, -0.05]	0	*
GrimAge	189	-0.14 [-0.16, -0.12]	0	*
3rd Generation	151	-0.13 [-0.15, -0.11]	0	*
DunedinPoAm	118	-0.13 [-0.15, -0.11]	0	*
DunedinPACE	33	-0.15 [-0.18, -0.11]	0	*

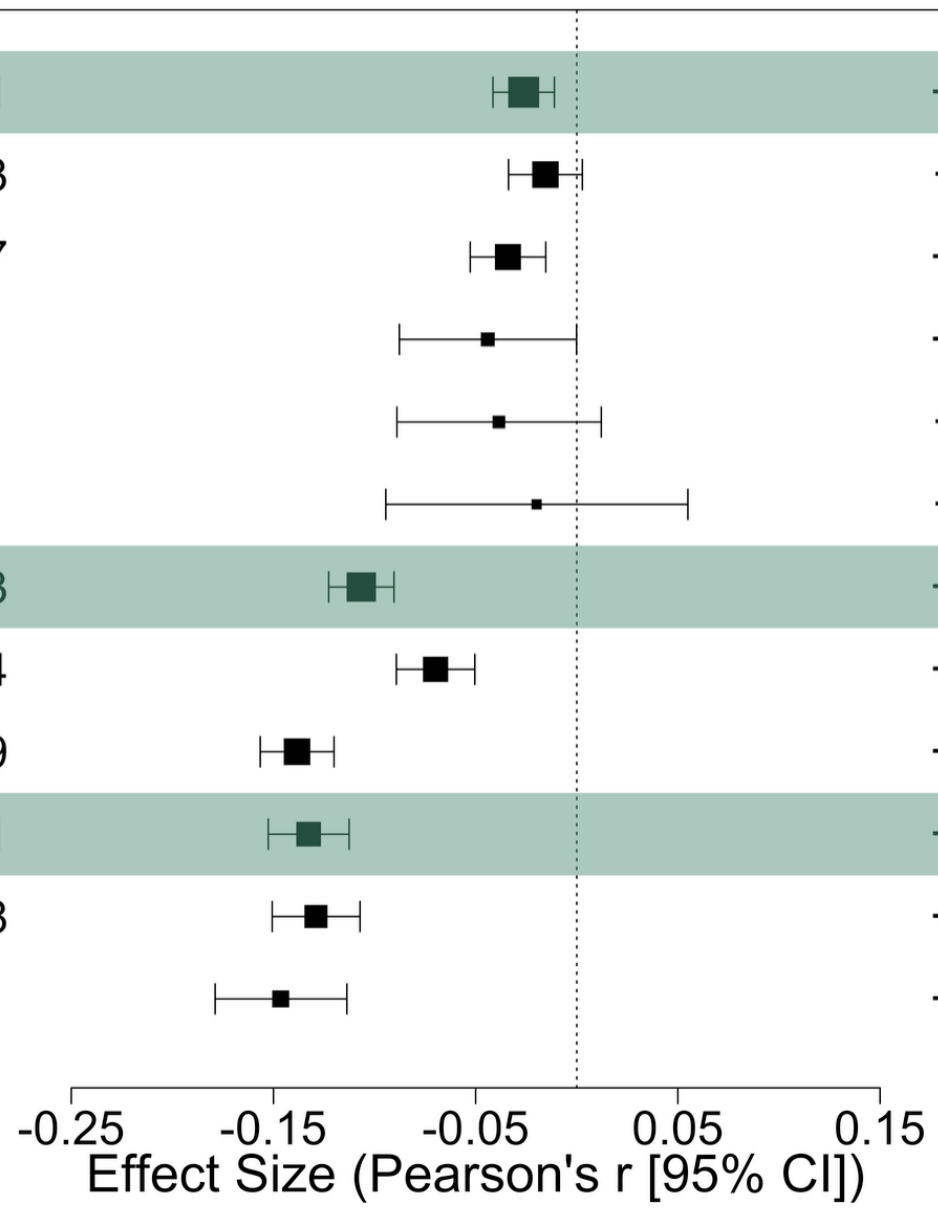

M. Meta-analytic associations between SES and epigenetic clocks (excluding effect sizes from My Body My Story)

DNAm Measure	Nr. of Effect Sizes	Estimate [95% CI]	p-value
1st Generation	376	-0.03 [-0.04, -0.01]	0 *
Horvath Pan-Tissue	174	-0.01 [-0.03, 0.00]	0.13
Hannum	161	-0.03 [-0.05, -0.02]	0 *
Horvath Skin & Blood	19	-0.05 [-0.09, -0.00]	0.04 *
Zhang	12	-0.05 [-0.11, 0.01]	0.12
PedBE	10	-0.02 [-0.10, 0.05]	0.59
2nd Generation	331	-0.11 [-0.13, -0.09]	0 *
PhenoAge	148	-0.07 [-0.09, -0.05]	0 *
GrimAge	183	-0.14 [-0.16, -0.12]	0 *
3rd Generation	141	-0.14 [-0.16, -0.12]	0 *
DunedinPoAm	108	-0.13 [-0.15, -0.11]	0 *
DunedinPACE	33	-0.15 [-0.18, -0.11]	0 *

-0.25 -0.15 -0.05 0.05 0.15

Effect Size (Pearson's r [95% CI])

N. Meta-analytic associations between SES and epigenetic clocks (excluding effect sizes from Project Viva)

DNAm Measure	Nr. of Effect Sizes	Estimate [95% CI]	p-value
1st Generation	388	-0.03 [-0.04, -0.01]	0 *
Horvath Pan-Tissue	175	-0.02 [-0.03, 0.00]	0.1
Hannum	165	-0.03 [-0.05, -0.02]	0 *
Horvath Skin & Blood	16	-0.04 [-0.08, 0.01]	0.12
Zhang	22	-0.04 [-0.09, 0.01]	0.13
PedBE	10	-0.02 [-0.09, 0.05]	0.6
2nd Generation	351	-0.11 [-0.12, -0.09]	0 *
PhenoAge	158	-0.07 [-0.09, -0.05]	0 *
GrimAge	193	-0.14 [-0.16, -0.12]	0 *
3rd Generation	151	-0.13 [-0.15, -0.11]	0 *
DunedinPoAm	118	-0.13 [-0.15, -0.11]	0 *
DunedinPACE	33	-0.15 [-0.18, -0.11]	0 *

-0.25 -0.15 -0.05 0.05 0.15

Effect Size (Pearson's r [95% CI])

O. Meta-analytic associations between SES and epigenetic clocks (excluding effect sizes from Swiss Kidney Project on Genes in Hypertension)

DNAm Measure	Nr. of Effect Sizes	Estimate [95% CI]	p-value	
1st Generation	406	-0.03 [-0.04, -0.01]	0	*
Horvath Pan-Tissue	184	-0.02 [-0.03, 0.00]	0.09	
Hannum	171	-0.03 [-0.05, -0.02]	0	*
Horvath Skin & Blood	19	-0.05 [-0.09, -0.00]	0.04	*
Zhang	22	-0.04 [-0.09, 0.01]	0.12	
PedBE	10	-0.02 [-0.10, 0.05]	0.59	
2nd Generation	333	-0.11 [-0.12, -0.09]	0	*
PhenoAge	149	-0.07 [-0.09, -0.05]	0	*
GrimAge	184	-0.14 [-0.16, -0.12]	0	*
3rd Generation	142	-0.14 [-0.16, -0.12]	0	*
DunedinPoAm	109	-0.13 [-0.16, -0.11]	0	*
DunedinPACE	33	-0.15 [-0.18, -0.11]	0	*

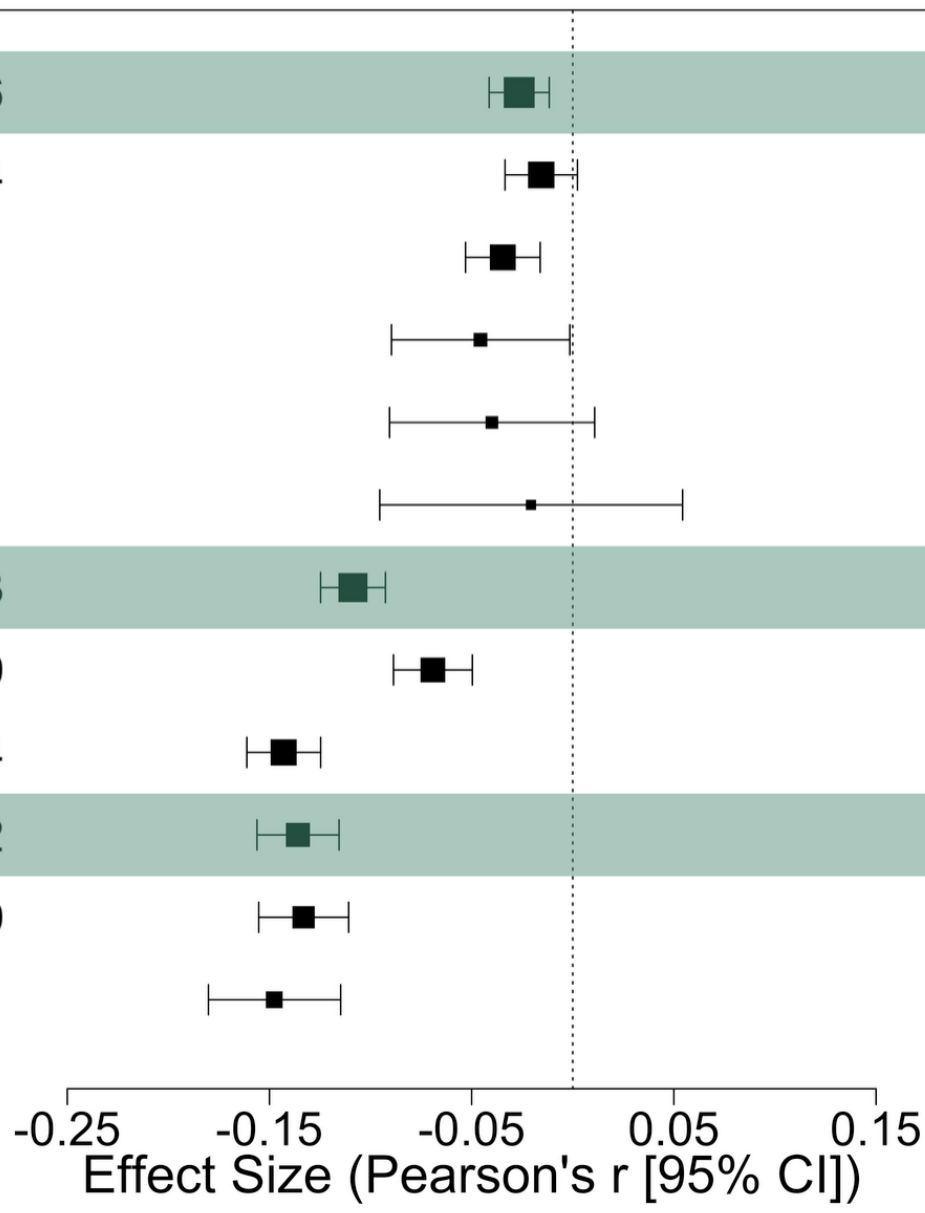

P. Meta-analytic associations between SES and epigenetic clocks (excluding effect sizes from Texas Twin Project)

DNAm Measure	Nr. of Effect Sizes	Estimate [95% CI]	p-value	
1st Generation	400	-0.03 [-0.04, -0.01]	0	*
Horvath Pan-Tissue	182	-0.01 [-0.03, 0.00]	0.13	
Hannum	169	-0.03 [-0.05, -0.02]	0	*
Horvath Skin & Blood	19	-0.04 [-0.09, -0.00]	0.05	*
Zhang	22	-0.04 [-0.09, 0.01]	0.14	
PedBE	8	-0.01 [-0.10, 0.08]	0.83	
2nd Generation	343	-0.11 [-0.13, -0.09]	0	*
PhenoAge	154	-0.07 [-0.09, -0.05]	0	*
GrimAge	189	-0.14 [-0.16, -0.13]	0	*
3rd Generation	141	-0.13 [-0.15, -0.11]	0	*
DunedinPoAm	110	-0.12 [-0.14, -0.10]	0	*
DunedinPACE	31	-0.16 [-0.19, -0.13]	0	*

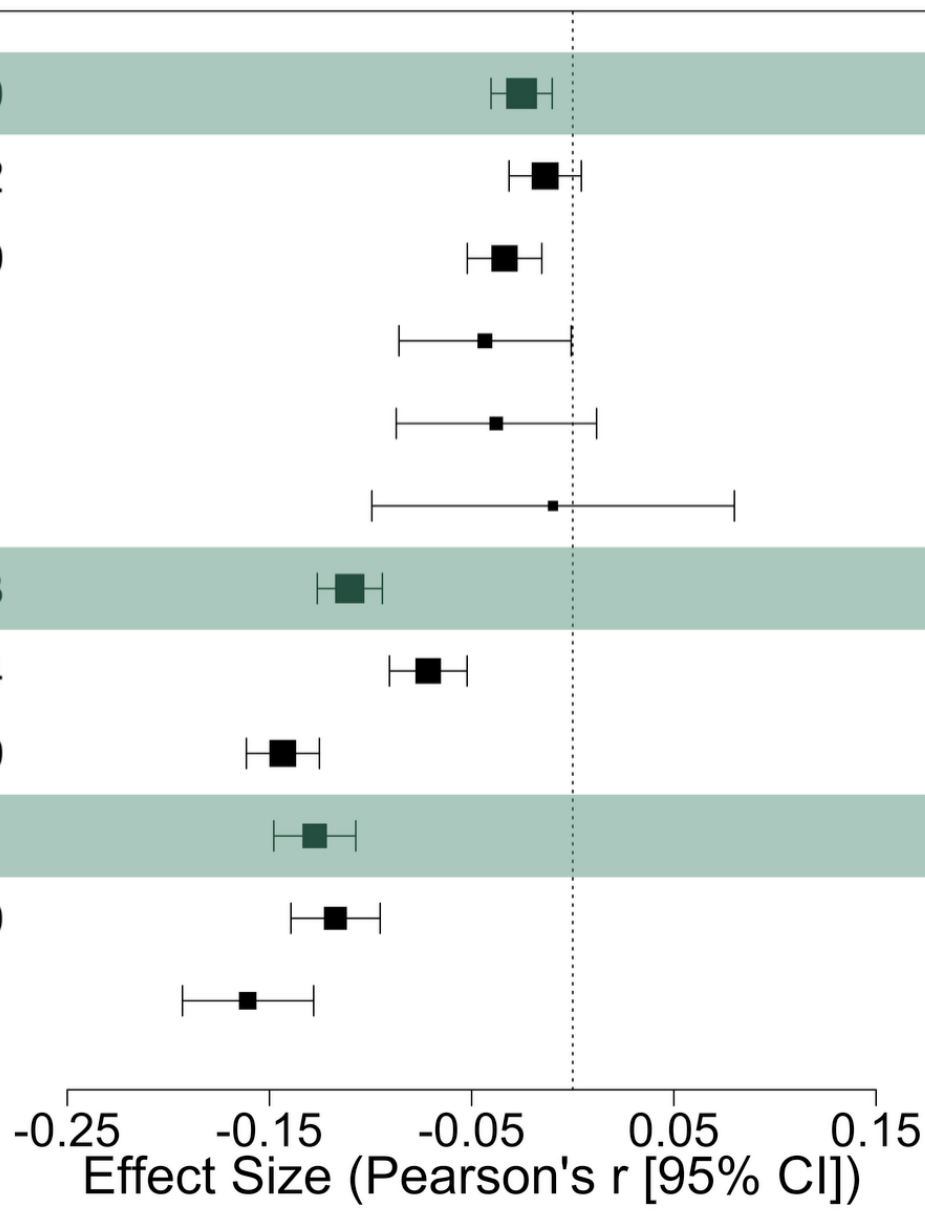

Q. Meta-analytic associations between SES and epigenetic clocks (excluding effect sizes from The Irish Longitudinal Study on Ageing)

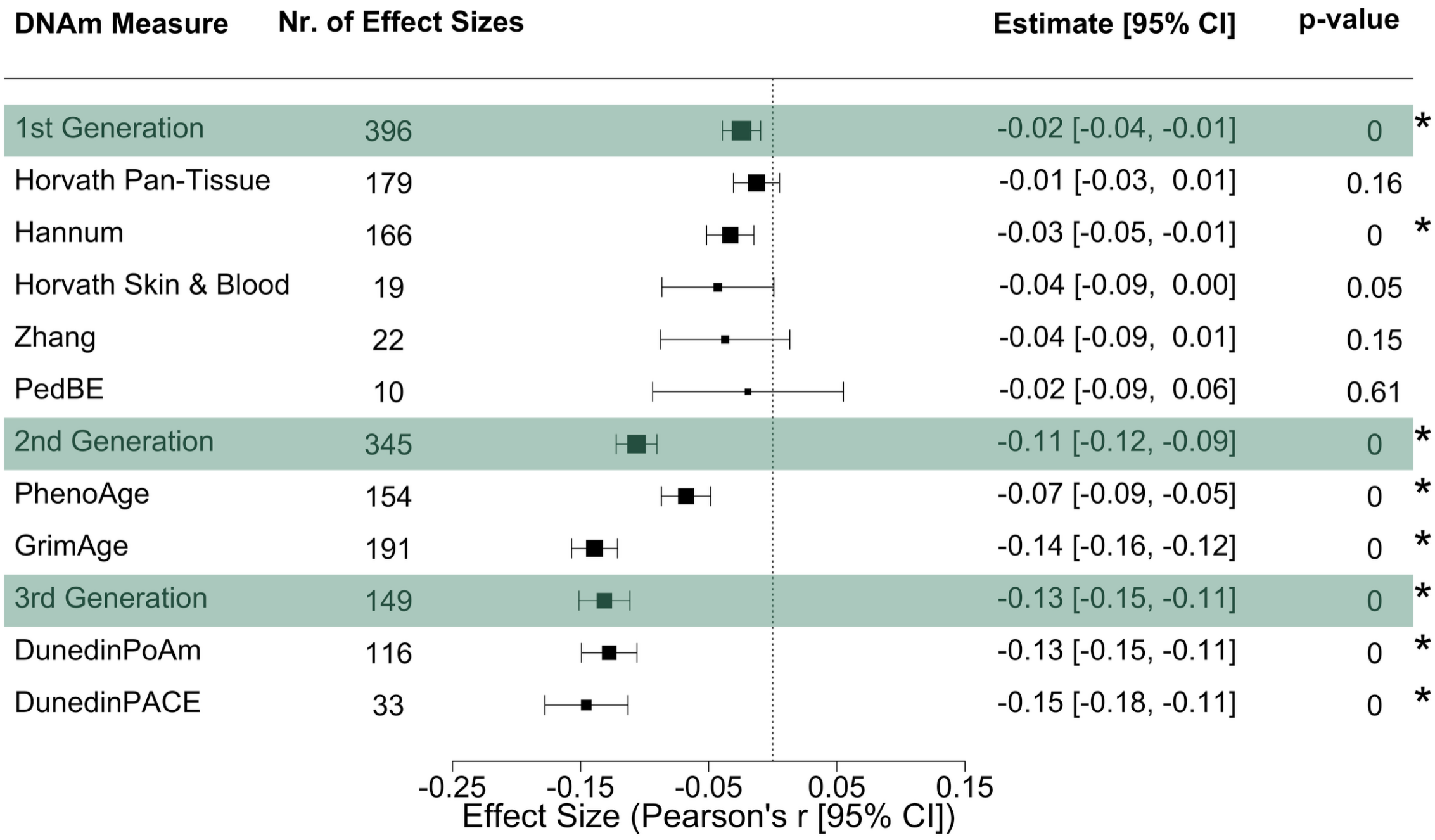

R. Meta-analytic associations between SES and epigenetic clocks (excluding effect sizes from Understanding Society)

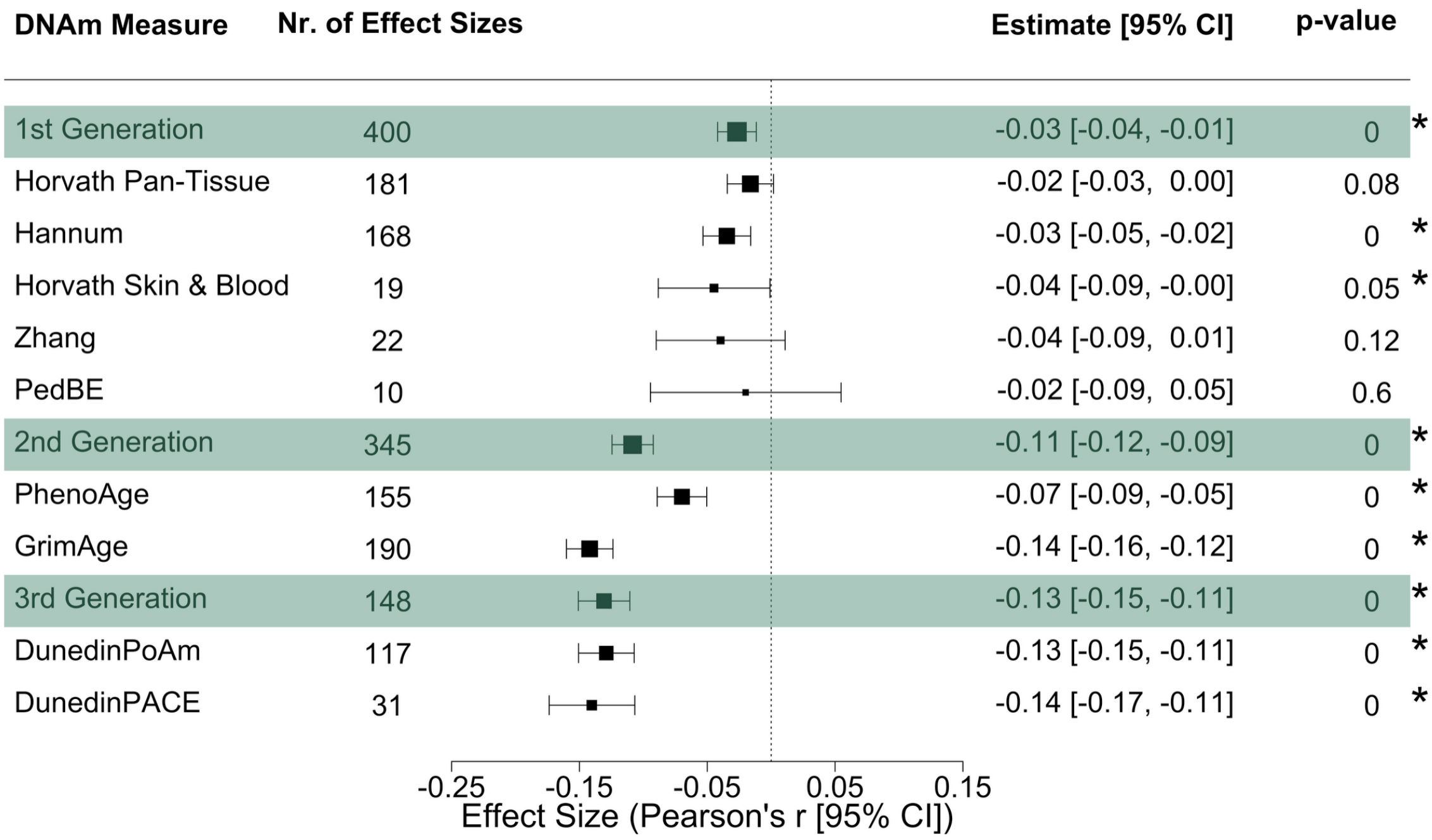

S. Meta-analytic associations between SES and epigenetic clocks (excluding effect sizes from Women's Health Initiative)

DNAm Measure	Nr. of Effect Sizes	Estimate [95% CI]	p-value	
1st Generation	404	-0.03 [-0.04, -0.01]	0	*
Horvath Pan-Tissue	183	-0.01 [-0.03, 0.00]	0.1	
Hannum	170	-0.03 [-0.05, -0.01]	0	*
Horvath Skin & Blood	19	-0.04 [-0.09, -0.00]	0.05	*
Zhang	22	-0.04 [-0.09, 0.01]	0.13	
PedBE	10	-0.02 [-0.09, 0.05]	0.6	
2nd Generation	351	-0.11 [-0.12, -0.09]	0	*
PhenoAge	158	-0.07 [-0.09, -0.05]	0	*
GrimAge	193	-0.14 [-0.16, -0.12]	0	*
3rd Generation	151	-0.13 [-0.15, -0.11]	0	*
DunedinPoAm	118	-0.13 [-0.15, -0.11]	0	*
DunedinPACE	33	-0.15 [-0.18, -0.11]	0	*

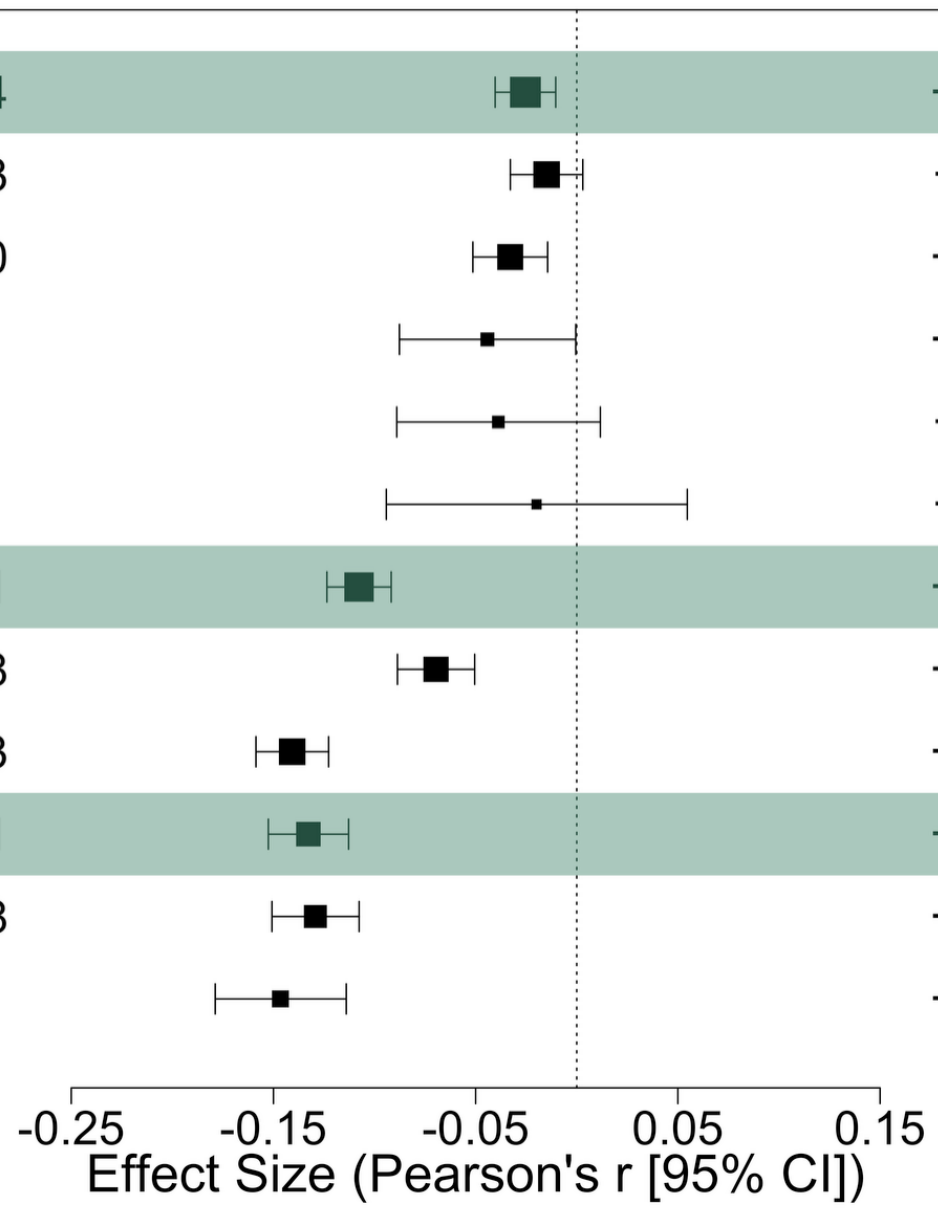

Supplemental Figure 17. Leave-One-Cohort-Out Analyses for the meta-analytic associations between SES and epigenetic clocks. Forest plot depicts the meta-analytic associations between SES and generations of epigenetic clocks excluding effect sizes from 19 cohorts that contributed at least 3 studies or at least 25 effect sizes. Green panels indicate the meta-analyzed effect size estimates for first-, second-, and third-generation clocks, followed by individual clocks within each category. Analyses were conducted using two-sided Fisher-Z transformation and later transformed to Pearson's r to ease interpretation. Effect size estimates are bounded by 95% confidence intervals (CIs).* denotes significant difference from 0 at $p < 0.05$ (not corrected for multiple comparisons). The size of the boxes in the middle panel represents the precision of the effect size estimate (i.e., the smaller the CI, the more precise the estimate).

A. Meta-analytic associations between Black- and White-identifying groups and epigenetic clocks excluding the Birmingham Youth Study

DNAm Measure	Nr. of Effect Sizes	Estimate [95% CI]	p-value	
1st Generation	29	-0.04 [-0.15, 0.07]	0.53	
Horvath Pan-Tissue	13	0.00 [-0.11, 0.11]	0.96	
Hannum	10	-0.10 [-0.22, 0.02]	0.09	
Horvath Skin & Blood	3	0.08 [-0.13, 0.29]	0.43	
Zhang	3	-0.55 [-0.97, -0.15]	0.01	*
2nd Generation	21	0.30 [0.19, 0.42]	0	*
PhenoAge	9	0.24 [0.12, 0.36]	0	*
GrimAge	12	0.35 [0.24, 0.47]	0	*
3rd Generation	14	0.42 [0.29, 0.55]	0	*
DunedinPoAm	7	0.40 [0.26, 0.53]	0	*
DunedinPACE	7	0.45 [0.32, 0.59]	0	*

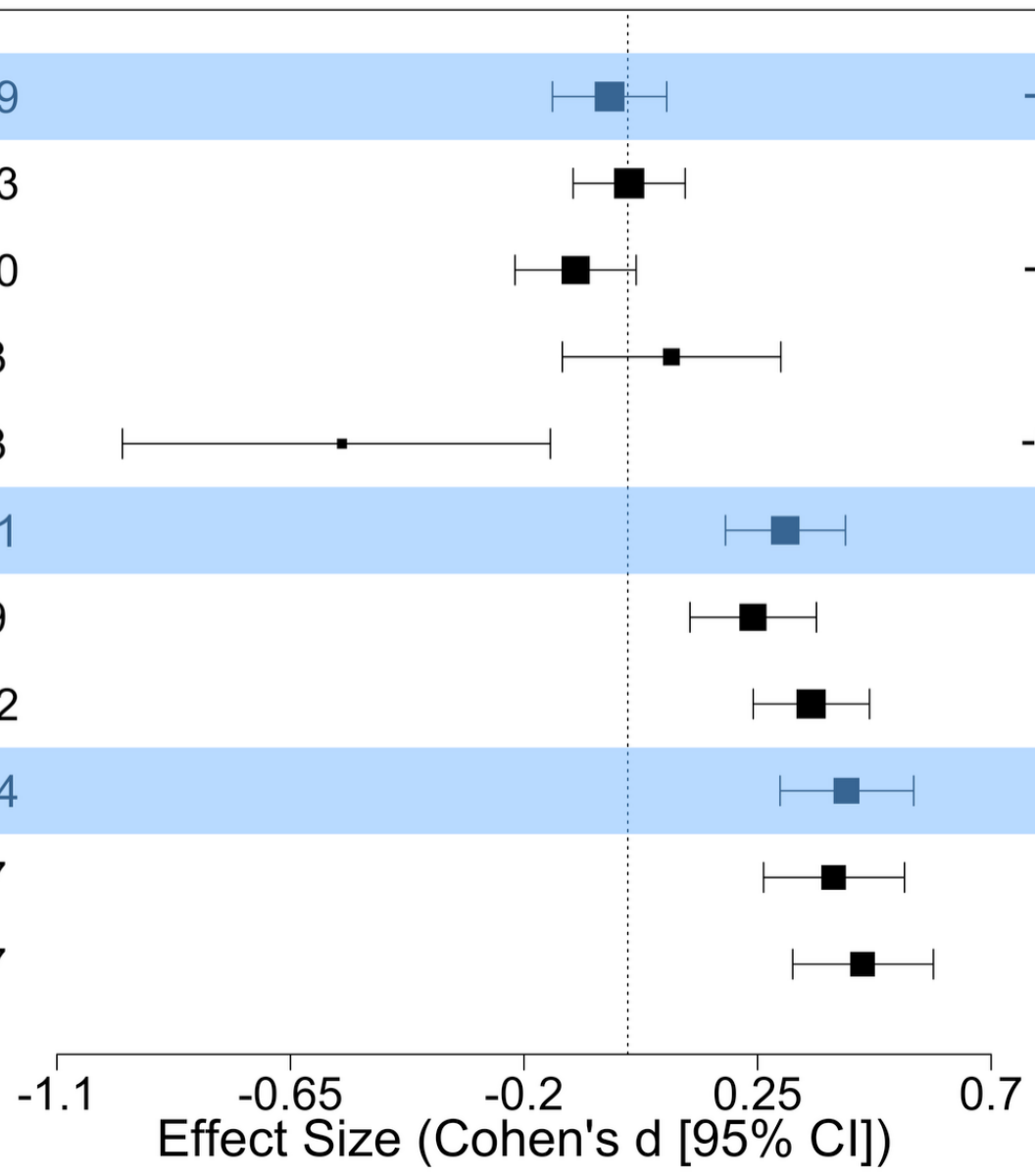

B. Meta-analytic associations between Black- and White-identifying groups and epigenetic clocks excluding Project Viva

DNAm Measure	Nr. of Effect Sizes	Estimate [95% CI]	p-value	
1st Generation	27	-0.06 [-0.17, 0.06]	0.32	
Horvath Pan-Tissue	12	-0.01 [-0.12, 0.10]	0.89	
Hannum	11	-0.12 [-0.23, -0.00]	0.05	*
Horvath Skin & Blood	1	0.03 [-0.25, 0.31]	0.83	
Zhang	3	-0.55 [-0.98, -0.14]	0.01	*
2nd Generation	23	0.29 [0.17, 0.40]	0	*
PhenoAge	10	0.22 [0.10, 0.34]	0	*
GrimAge	13	0.35 [0.24, 0.46]	0	*
3rd Generation	14	0.40 [0.27, 0.54]	0	*
DunedinPoAm	7	0.38 [0.25, 0.52]	0	*
DunedinPACE	7	0.44 [0.30, 0.58]	0	*

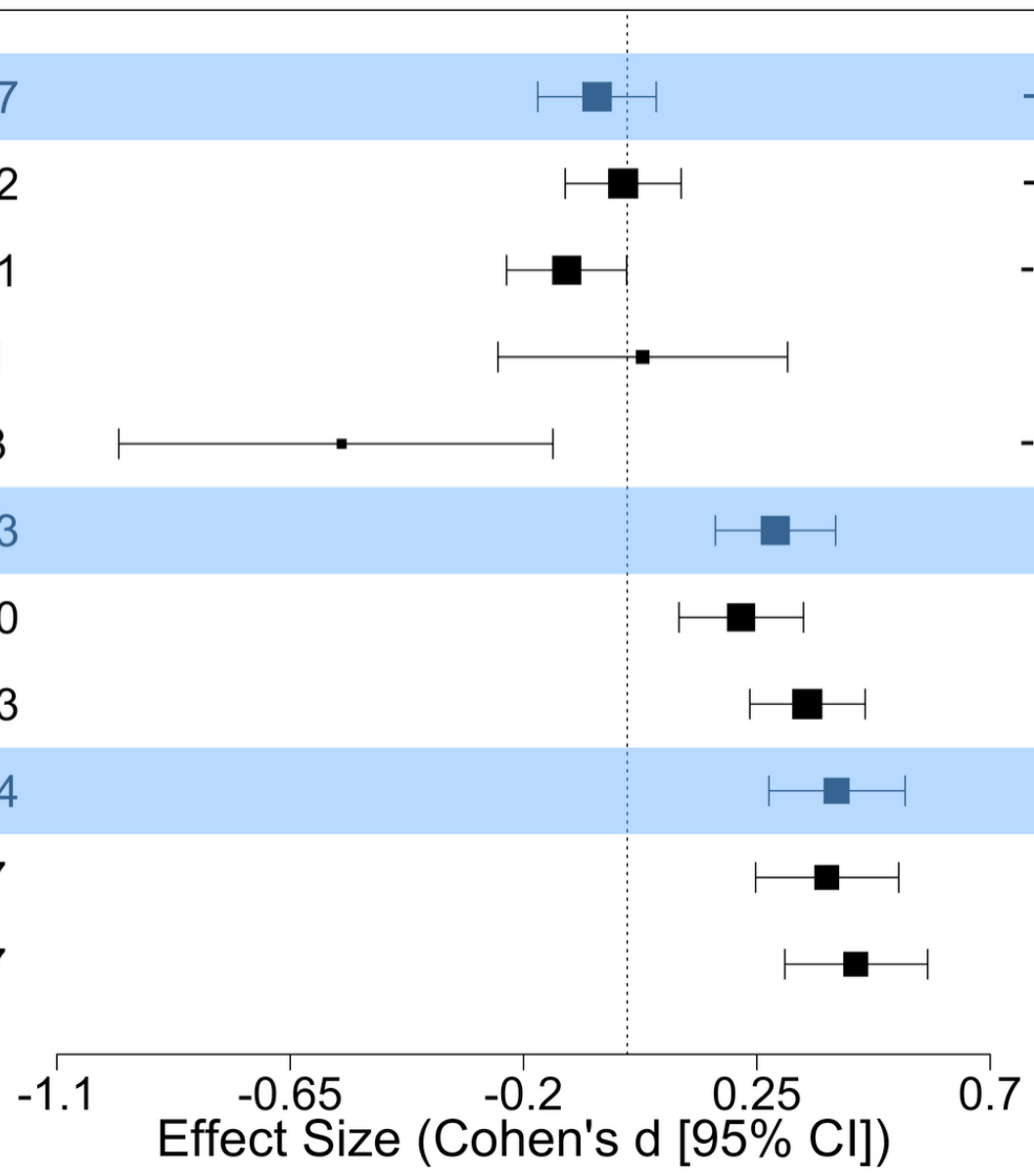

C. Meta-analytic associations between Black- and White-identifying groups and epigenetic clocks excluding the Texas Twin Project

DNAm Measure	Nr. of Effect Sizes	Estimate [95% CI]	p-value	
1st Generation	31	-0.06 [-0.17, 0.06]	0.33	
Horvath Pan-Tissue	14	-0.03 [-0.13, 0.07]	0.57	
Hannum	11	-0.13 [-0.25, -0.02]	0.02	*
Horvath Skin & Blood	3	0.06 [-0.15, 0.27]	0.57	
Zhang	3	-0.55 [-0.96, -0.16]	0.01	*
2nd Generation	21	0.28 [0.16, 0.40]	0	*
PhenoAge	9	0.19 [0.07, 0.31]	0	*
GrimAge	12	0.34 [0.23, 0.45]	0	*
3rd Generation	11	0.41 [0.26, 0.55]	0	*
DunedinPoAm	5	0.34 [0.19, 0.50]	0	*
DunedinPACE	6	0.45 [0.31, 0.59]	0	*

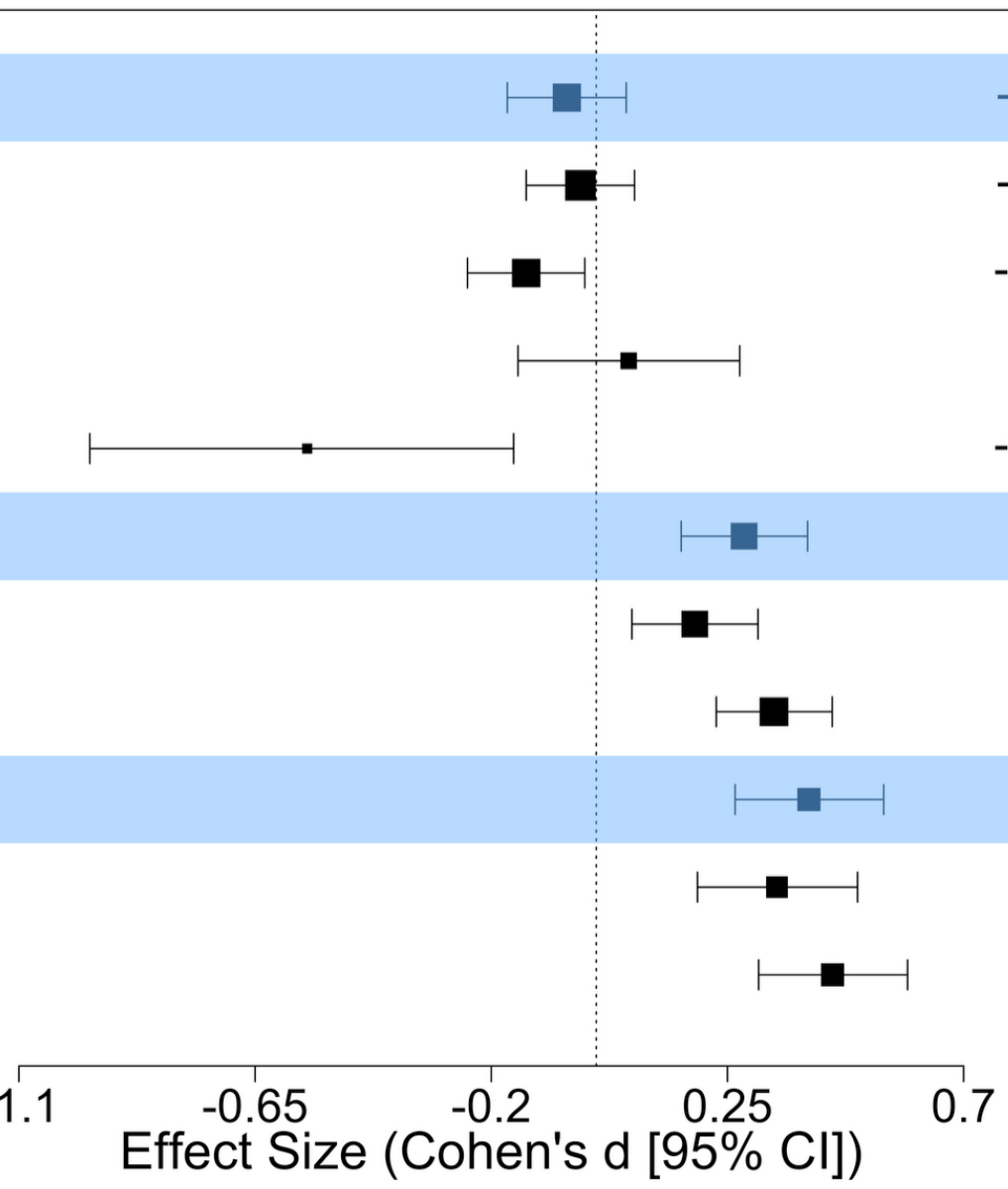

D. Meta-analytic associations between Black- and White-identifying groups and epigenetic clocks excluding the Women's Health Initiative

DNAm Measure	Nr. of Effect Sizes	Estimate [95% CI]	p-value	
1st Generation	27	-0.03 [-0.14, 0.07]	0.53	
Horvath Pan-Tissue	12	-0.01 [-0.12, 0.10]	0.88	
Hannum	9	-0.08 [-0.20, 0.04]	0.2	
Horvath Skin & Blood	3	0.08 [-0.13, 0.29]	0.46	
Zhang	3	-0.55 [-0.96, -0.16]	0.01	*
2nd Generation	22	0.31 [0.19, 0.42]	0	*
PhenoAge	9	0.23 [0.10, 0.35]	0	*
GrimAge	13	0.36 [0.25, 0.47]	0	*
3rd Generation	14	0.42 [0.30, 0.55]	0	*
DunedinPoAm	7	0.40 [0.26, 0.53]	0	*
DunedinPACE	7	0.45 [0.32, 0.59]	0	*

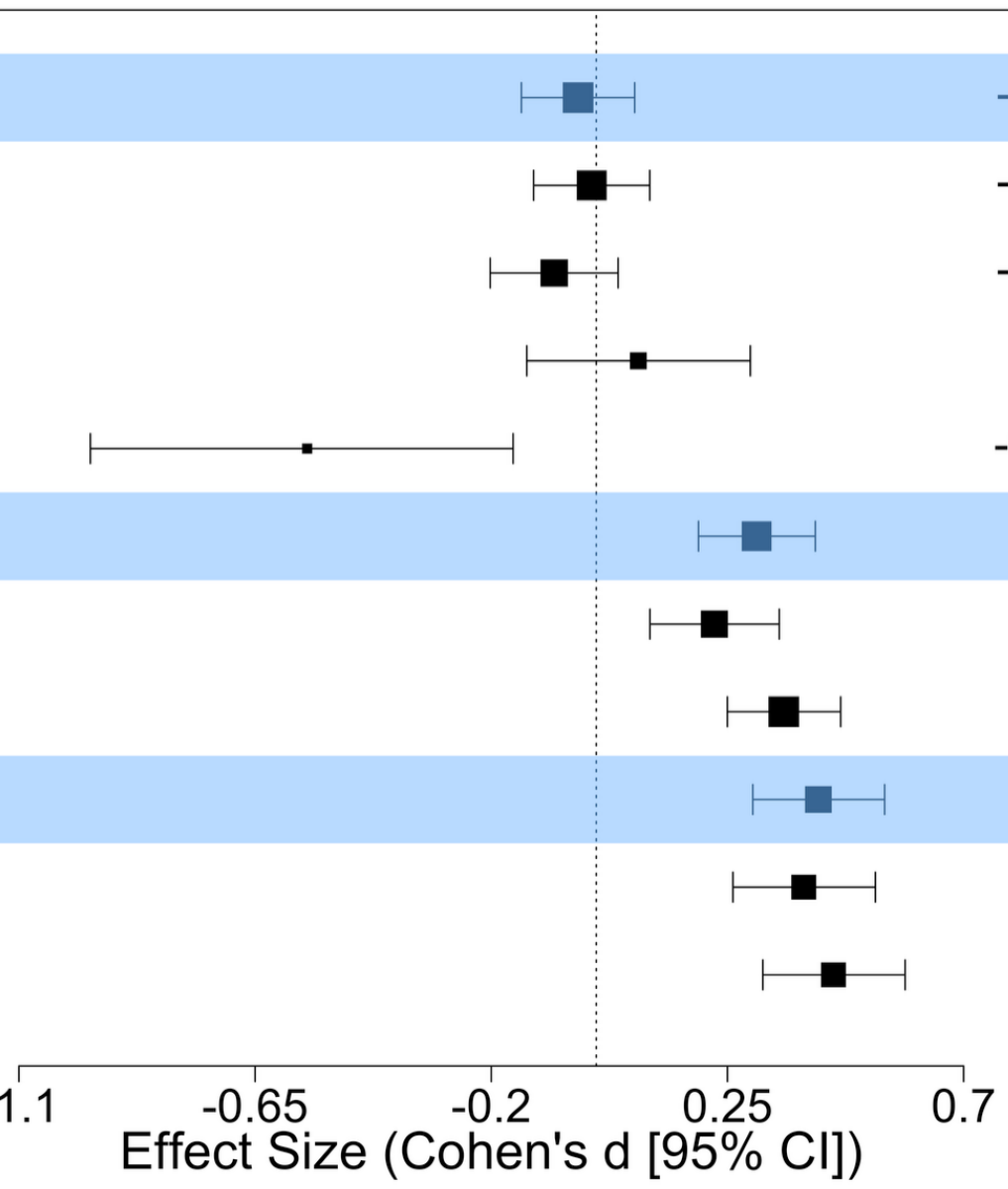

Supplemental Figure 18. Leave-One-Cohort-Out Analyses for the meta-analytic associations between Black- and White-identifying groups and epigenetic clocks.

Forest plots depict the meta-analytic Black-White comparisons and epigenetic clocks excluding effect sizes from cohorts that contributed at least 3 studies or at least 25 effect sizes. Excluding (A) Birmingham Youth Study, (B) Project Viva, (C) Texas Twin Project, (D) Women's Health Initiative. Blue panels indicate the meta-analyzed effect size estimates for first-, second-, and third-generation clocks, followed by individual clocks within each category. Analyses were conducted using two-sided Fisher-Z transformation and later transformed to Cohen's d to ease interpretation. Effect size estimates are bounded by 95% confidence intervals (CIs).* denotes significant difference from 0 at $p < 0.05$ (not corrected for multiple comparisons). The size of the boxes in the middle panel represents the precision of the effect size estimate (i.e., the smaller the CI, the more precise the estimate).

A. Meta-analytic associations between Latinx- and White-identifying groups and epigenetic clocks excluding Project Viva

DNAm Measure	Nr. of Effect Sizes	Estimate [95% CI]	p-value	
1st Generation	13	0.04 [-0.10, 0.18]	0.58	
Horvath Pan-Tissue	6	-0.07 [-0.23, 0.10]	0.44	
Hannum	6	0.19 [0.02, 0.35]	0.03	*
Horvath Skin & Blood	1	0.09 [-0.11, 0.30]	0.37	
2nd Generation	13	0.05 [-0.09, 0.19]	0.5	
PhenoAge	7	0.08 [-0.09, 0.24]	0.37	
GrimAge	6	0.06 [-0.11, 0.23]	0.48	
3rd Generation	11	0.24 [0.09, 0.38]	0	*
DunedinPoAm	6	0.16 [-0.01, 0.33]	0.06	
DunedinPACE	5	0.30 [0.13, 0.48]	0	*

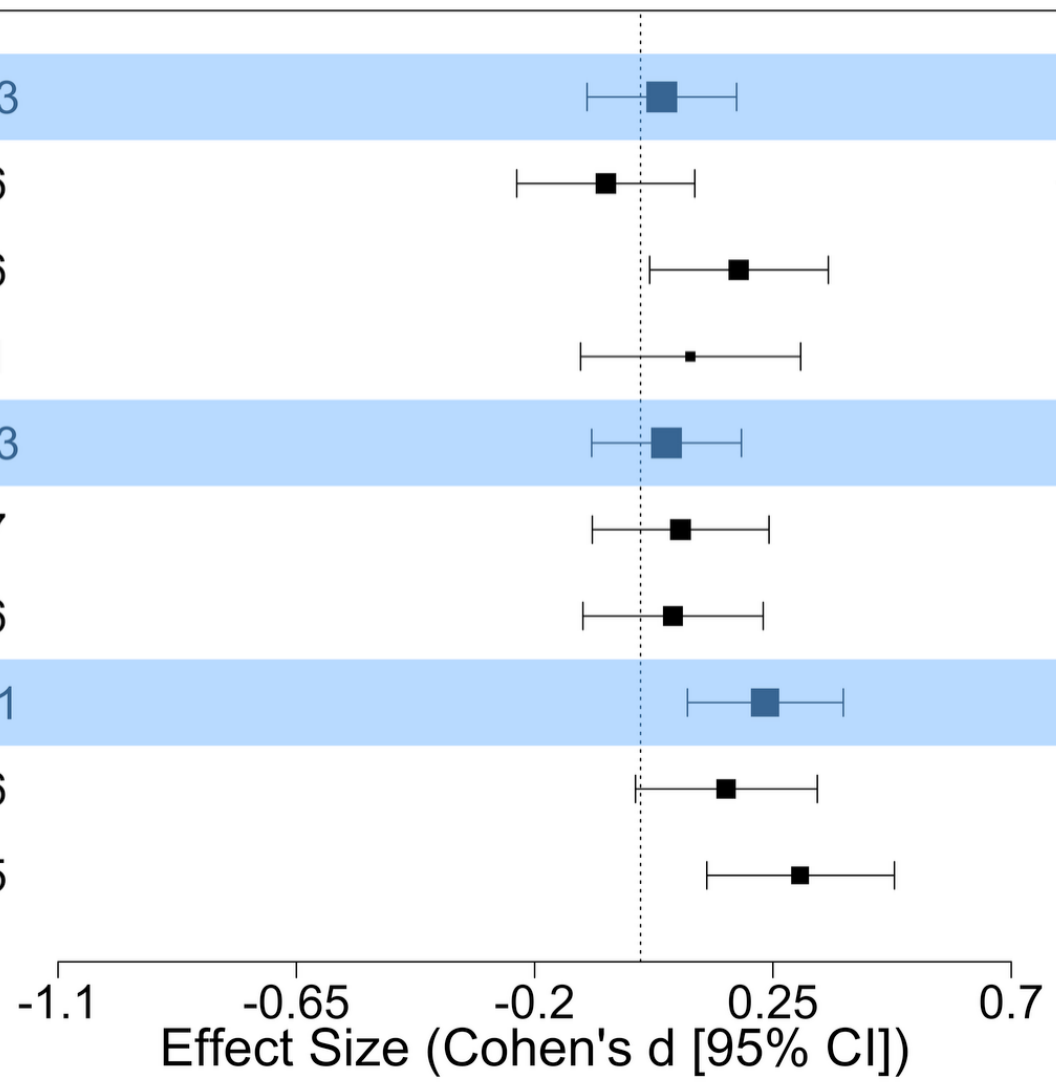

B. Meta-analytic associations between Latinx- and White-identifying groups and epigenetic clocks excluding the Texas Twin Project

DNAm Measure	Nr. of Effect Sizes	Estimate [95% CI]	p-value	
1st Generation	17	0.03 [-0.08, 0.15]	0.58	
Horvath Pan-Tissue	8	-0.08 [-0.22, 0.07]	0.32	
Hannum	6	0.17 [0.02, 0.33]	0.03	*
Horvath Skin & Blood	3	0.11 [-0.07, 0.29]	0.22	
2nd Generation	11	0.03 [-0.10, 0.16]	0.66	
PhenoAge	6	0.07 [-0.08, 0.22]	0.35	
GrimAge	5	0.05 [-0.10, 0.21]	0.52	
3rd Generation	7	0.19 [0.04, 0.33]	0.01	*
DunedinPoAm	3	0.09 [-0.08, 0.26]	0.3	
DunedinPACE	4	0.30 [0.14, 0.46]	0	*

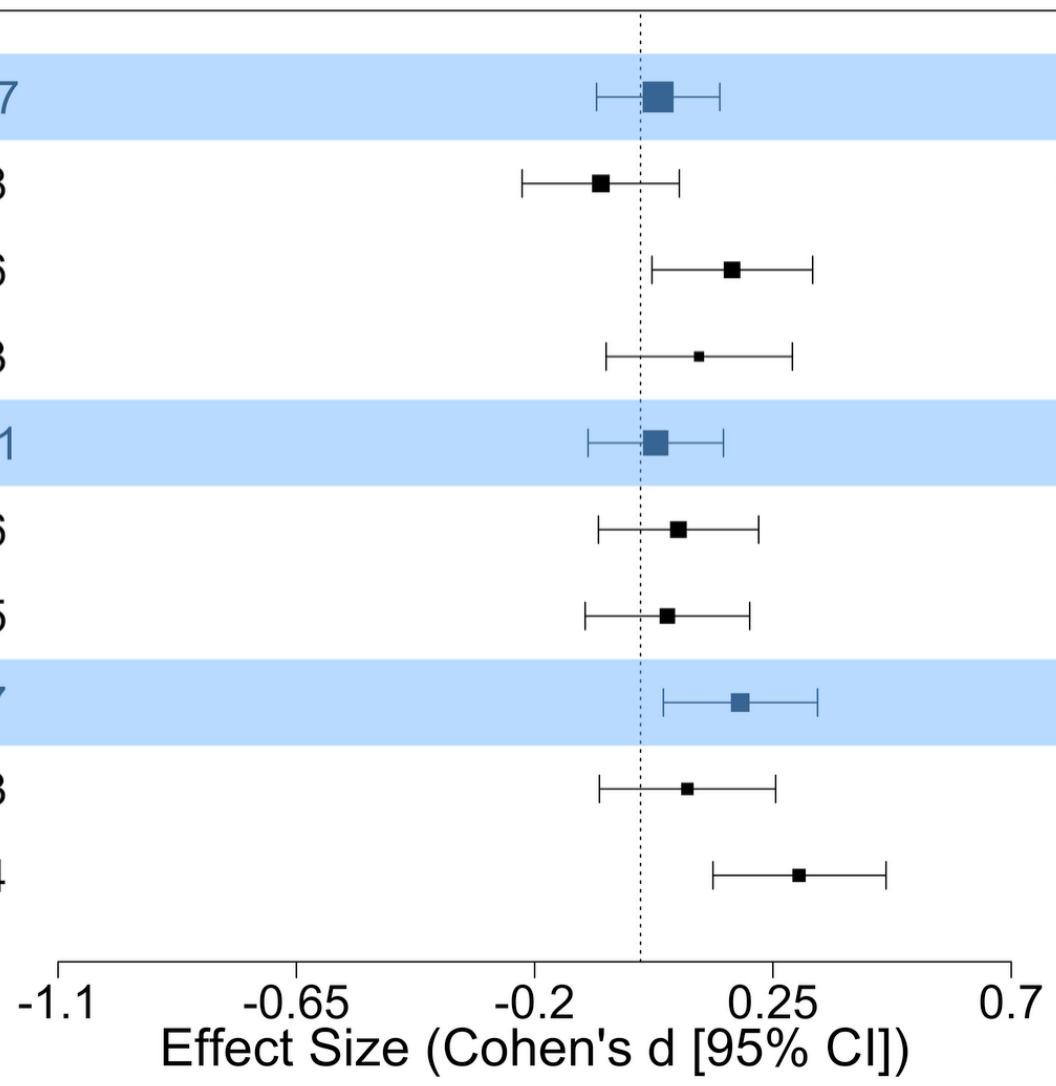

C. Meta-analytic associations between Black- and White-identifying groups and epigenetic clocks excluding the Women's Health Initiative

DNAm Measure	Nr. of Effect Sizes	Estimate [95% CI]	p-value	
1st Generation	15	0.12 [-0.04, 0.28]	0.15	
Horvath Pan-Tissue	7	0.04 [-0.15, 0.23]	0.67	
Hannum	5	0.25 [0.06, 0.44]	0.01	*
Horvath Skin & Blood	3	0.19 [-0.01, 0.39]	0.07	
2nd Generation	12	0.10 [-0.06, 0.27]	0.22	
PhenoAge	6	0.14 [-0.05, 0.33]	0.15	
GrimAge	6	0.14 [-0.05, 0.33]	0.15	
3rd Generation	11	0.29 [0.12, 0.45]	0	*
DunedinPoAm	6	0.22 [0.03, 0.42]	0.02	*
DunedinPACE	5	0.37 [0.18, 0.57]	0	*

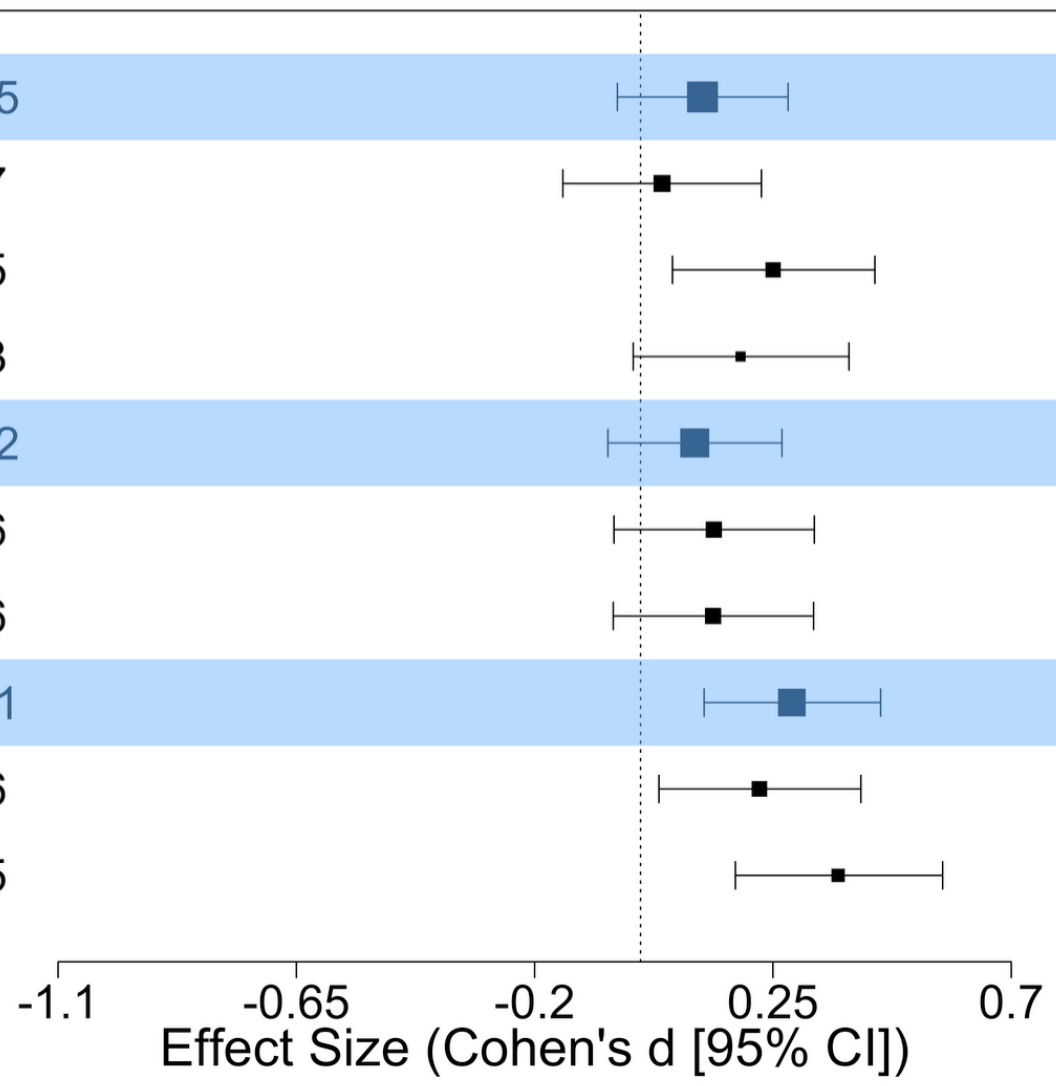

Supplemental Figure 19. Leave-One-Cohort-Out Analyses for the meta-analytic associations between Latinx- and White-identifying groups and epigenetic clocks.
Forest plots depict the meta-analytic Latinx-White comparisons and epigenetic clocks excluding effect sizes from cohorts that contributed at least 3 studies or at least 25 effect sizes. Excluding (A) Project Viva, (B) Texas Twin Project, (C) Women's Health Initiative. Blue panels indicate the meta-analyzed effect size estimates for first-, second-, and third-generation clocks, followed by individual clocks within each category. Analyses were conducted using two-sided Fisher-Z transformation and later transformed to Cohen's d to ease interpretation. Effect size estimates are bounded by 95% confidence intervals (CIs).* denotes significant difference from 0 at $p < 0.05$ (not corrected for multiple comparisons). The size of the boxes in the middle panel represents the precision of the effect size estimate (i.e., the smaller the CI, the more precise the estimate).

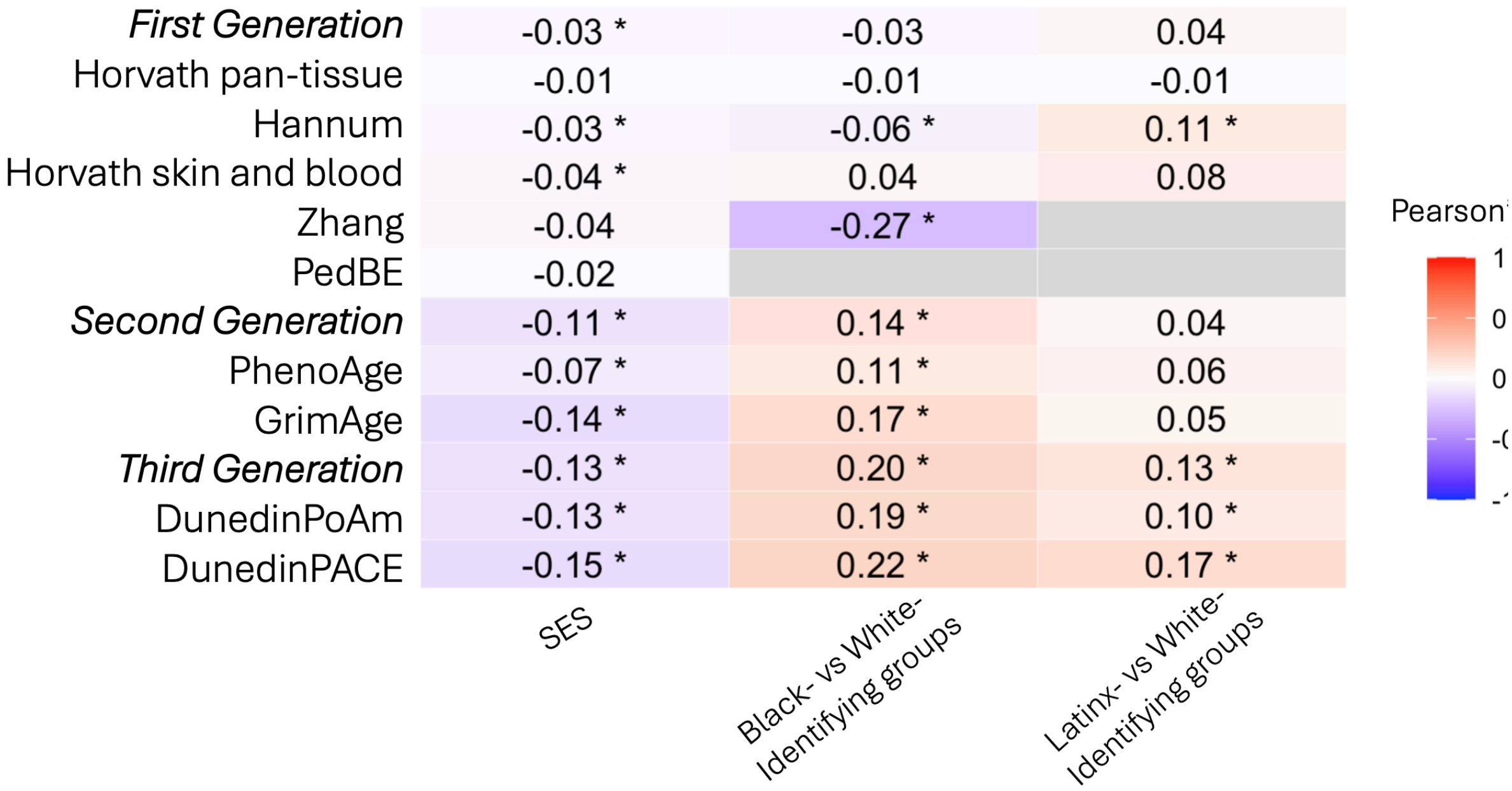

Supplemental Figure 20. Pearson's *r* values for the associations between SES/race/ethnicity and DNAm generations and measures of biological aging. *Note:* Estimates reflect Pearson's r to compare estimates across social determinants of health. Estimate's in Cohen's d can be found in Figure 4.
