# Supplementary material for: Social determinants of health and epigenetic clocks: a systematic review and meta-analysis of 140 studies
Source: Nat Hum Behav. 2026 Jun 12;10(10):2016–28. doi: 10.1038/s41562-026-02477-6 (PMC13683040; doi:10.1038/s41562-026-02477-6)
Supplement: Supplementary file 3 [file 41562_2026_2477_MOESM3_ESM.pdf]

nature portfolio

Peer Review File

Social determinants of health and epigenetic clocks: A systematic review and meta-analysis of 140 studies

Corresponding Author: Dr Laurel Raffington

Version 0:

Decision Letter:

27th August 2025

Dear Dr Raffington,

Thank you once again for your manuscript, entitled "Social determinants of health and epigenetic clocks: Meta-analysis of 140 studies," and for your patience during the peer review process.

Your manuscript has now been evaluated by 3 reviewers, whose comments are included at the end of this letter. Although the reviewers find your work to be of interest, they also raise some important concerns. We are very interested in the possibility of publishing your study in Nature Human Behaviour, but would like to consider your response to these concerns in the form of a revised manuscript before we make a decision on publication.

Editorially we ask that in your revised manuscript you: update your search, adjust for multiple comparisons, report risk of bias assessment, remove causal language, examine the influence of inconsistent covariate control, systematically consider the effect of tissue type, and acknowledge limitations (e.g. dependence on US cohorts for race/ethnicity estimates).

Finally, your revised manuscript must comply fully with our editorial policies and formatting requirements. Failure to do so will result in your manuscript being returned to you, which will delay its consideration. To assist you in this process, I have attached a checklist that lists all of our requirements. If you have any questions about any of our policies or formatting, please don't hesitate to contact me.

In sum, we invite you to revise your manuscript taking into account all reviewer and editor comments. We are committed to providing a fair and constructive peer-review process. Do not hesitate to contact us if there are specific requests from the reviewers that you believe are technically impossible or unlikely to yield a meaningful outcome.

We hope to receive your revised manuscript within two months. I would be grateful if you could contact us as soon as possible if you foresee difficulties with meeting this target resubmission date.

With your revision, please:

• Include a “Response to the editors and reviewers” document detailing, point-by-point, how you addressed each editor and referee comment. If no action was taken to address a point, you must provide a compelling argument. When formatting this document, please respond to each reviewer comment individually, including the full text of the reviewer comment verbatim followed by your response to the individual point. This response will be used by the editors to evaluate your revision and sent back to the reviewers along with the revised manuscript.

• Highlight all changes made to your manuscript or provide us with a version that tracks changes.

• EXTENDED DATA FIGURES
When re-submitting your manuscript, please ensure that any supplementary figures and tables that are crucial to the manuscript’s conclusions are converted into Extended Data figures and tables to increase visibility of these data. Extended Data figures and tables are online-only (present in the online PDF and full-text HTML versions of the paper), peer-reviewed display items that provide essential background to the article but are not included in the main article due to space constraints. A maximum of ten Extended Data display items (figures and tables) is permitted.

Please use the link below to submit your revised manuscript and related files:

Link Redacted

Note: This URL links to your confidential home page and associated information about manuscripts you may have submitted, or that you are reviewing for us. If you wish to forward this email to co-authors, please delete the link to your homepage.

We look forward to seeing the revised manuscript and thank you for the opportunity to review your work. Please do not hesitate to contact me if you have any questions or would like to discuss these revisions further.

Sincerely,

[redacted]

[redacted]
Nature Human Behaviour

Reviewer expertise:

Reviewer #1: epigenetic aging

Reviewer #2: epigenetic clocks, biological aging

Reviewer #3: meta-analysis, aging, epidemiology

REVIEWER COMMENTS:

Reviewer #1 (Remarks to the Author):

The authors provide a comprehensive meta-analysis of associations between various methylation-based indicators of biological aging with SES and race/ethnicity. They find associations between accelerated aging with lower SES and for Black and Latinx relative to White identifying groups. Associations tended to be larger for more recent measures, especially the two Dunedin indices and GrimAge. In addition, differences across age, sex, and technical factors were explored. It is very valuable to have these new meta-analytic estimates. The pre-registered analyses are well executed with only very minor deviations. I appreciate how well the results are reported in thorough tables and elegant and intuitive figures. I think this is a great contribution and I have only a few suggestions.

My primary concern is the framing of the "speedometers" outperforming the second-generation clocks as a key result. While this is technically true, it is driven by their specific outperformance of PhenoAge, as they rarely (if ever?) significantly outperform GrimAge. In prior studies, GrimAge tends to correlate more strongly with DunedinPACE than with PhenoAge. While I have no issue with framing the Dunedin indices as a distinctive generation of speedometers, I found a few of the claims to be too strong in light of this pattern of results. The incremental validity may well be related to the potency of the training phenotypes for both GrimAge and DunedinPACE/PoAm relative to the other (even second generation) measures rather than indexing pace as opposed to accumulation. Therefore, I found it slightly misleading to include the following in the Abstract:
"The strongest associations were observed for a third generation of epigenetic clocks, sometimes referred to as "epigenetic speedometers", developed to predict the pace of aging"

Similarly, I think the following overstates the pace versus accumulation distinction in the Discussion, given that neither Dunedin index had significantly larger associations than GrimAge in the analyses of childhood DNAm:
"One interpretation of these data is that acceleration to aging processes caused by early adversity is immediately apparent in "speedometer" type third-generation clocks, but does not become apparent until later in life in second-generation clocks. Collectively, findings suggest that third-generation clocks, such as DunedinPACE, are the most promising tools for tracing environmental impacts on aging and health early in life"

I'd suggest tempering the language that highlights second and third generation differences and providing some interpretation as to why GrimAge also appears to have strong predictive validity relative to other measures.

Overall, I thought the Introduction was great – a concise and effective summary. My suggestions for it are minor.
1. I think that saying epigenetic clocks are "among the most widely studied and best-validated predictors of healthy lifespan across a range of human populations" likely overstates their predictive power. Is there any evidence they outperform indices of aging like cognition, adaptive functioning, or inflammation in predicting mortality?

2. I thought more rationale could be given studying age differences. For instance, how does the concept of biological aging apply in childhood? Do developmental researchers think of things like early puberty as similar to biological age exceeding chronological age late in life?

3. I'm curious how the authors would feel about framing hypotheses 4 and 5 as more supplemental, rather than as central as the first three. Because the analyses related to sex differences and technical factors were hampered by methodological issues outside of the authors' control (I appreciate the transparency in noting this), I found myself less compelled and putting less stock into them.

Similarly, I thought the presentation of methods and results was excellent. I had one point of confusion that I think the authors could clear up in a revision, and one idea to consider.
1. I had a hard time following the number of effect sizes. I am probably missing something, but I summed 908 effect sizes in the SES analyses and an additional 109 for the race/ethnicity analyses. This sums to 1,017, which is less than the reported 1,065. In addition, the authors write "DNAm collected in adulthood (83% of effect sizes) is more common than DNAm in childhood (15%)", so I was curious why this didn't sum to 100%. In the figure, it looks 113 of 908 SES effect sizes were from childhood DNAm samples (12.4%). I think a sentence or two that clarifies what all went into the 1,065 figure (and how it is split up) would be helpful.

2. I thought Supplemental Table 3C summarized many of the key results in a nice and concise manner. I found myself going back to it more than the other tables. I wonder what the authors would think about making it more central and perhaps including it in the main text.

My only suggestion for the Discussion is also minor, aside from the concern discussed above.
1. I thought more attention could have been given to the null finding for repeated methylation measurements. I'd be curious what the other reviewers and editors think. While I understand there were fewer studies and results were less precise, I think these analyses are central to the endeavor as outlined in the Introduction (i.e., how responsive is biological aging to environmental change?). I think some discussion of the possibility that longitudinal associations may be null is worth exploring.

Thank you,
Colin Freilich

Reviewer #2 (Remarks to the Author):

This meta-analysis investigates the association between social determinants of biological aging (socioeconomic status (SES) and race/ethnicity) and biological aging as measured by epigenetic clocks. In addition, the authors explore potential moderators (e.g.. clock generation, timing, technical factors) of these associations. The meta-analysis uses summarized data from 140 studies comprising 65,919 participants. Lower SES and marginalized race/etnicity were associated with accelerated biological aging. The association of SES was strongest for second- and third-generation measures, and only third-generation clocks indicated association in children. To the best of my knowledge, there are no meta-analyses comprehensively examining the associations between SES/race and biological aging using DNAm-based measures from all three generations. Overall, the manuscript is well-written and presents a substantial amount of interesting and valuable information. My specific comments and concerns are listed below:

Abstract
Does the phrase "Effects of sex and technical factors were minimal…" refer to modifying effects of these factors? This should be clarified.

Introduction:

The term 'determinant' may imply a causal relationship. However, to my knowledge, there is no conclusive evidence establishing a causal effect of SES and biological aging. The present meta-analysis uses effect sizes from observational studies. It is also plausible that individuals with better underlying health are more likely to attain higher SES. I believe it would be appropriate at least briefly to acknowledge the potential confounding role of factors such as genetic influences in these associations.

Are the authors aware of any previous meta-analyses that have assessed the associations between socioeconomic status (SES) or race/ethnicity and biological aging as measured by epigenetic clocks?

Discussion:

If studies based on the latest EPICv2 platform were excluded, this should be acknowledged in the limitations section.

It would be appropriate to acknowledge that the meta-analysis was limited to studies from high-income countries thus potentially limiting the generalizability of findings.

The authors conclude that ”In children, only the third -generation speedometers showed consisted associations”, and suggest that their findings provide guidance for selecting epigenetic clocks for future studies. However, in the included child studies, the DNAm was primarily measured using saliva samples. It remains unclear whether the superior performance of the third-generation speedometers would hold if blood-based DNAm data were used instead in children.

Methods:
Based on the data provided in the supplement, there appears to be an inconsistency in the coding of the variable ‘Array’. According to the instructions, the categories are defined as: 1=EPIC, 2=EPIC v2, 3=450k, 0=n/a. However, the dataset contains values 0,1,3,4. In Table 3, the chip versions are 450k, EPIC, 450k and EPIC, n/a. Therefore, it is unclear whether studies using EPIC v2 platform were included in the meta-analysis, and if so, whether they were analysed together with studies using EPIC v1. This should be clarified. Additionally, it would be helpful to know whether the authors examined potential differences in associations across these platforms, as there are EPIC v2 has been shown to perform differently from earlier version (Tay et al., 2025).

Reference:
Tay, J. H., Chew, Y. E., Wang, W., Lim, Z. M., Guan, L., Dorajoo, R., Kennedy, B. K., Brooke, R., Gordevicius, J., Horvath, S., Sandalova, E., & Maier, A. B. (2025). DNAm age differences between infinium methylationEPICv1 vs EPICv2 in buffy coat, PBMC, and saliva samples. Communications Biology, 8(1). https://doi.org/10.1038/s42003-025-08021-y

Statistical analysis
If I understood correctly, the moderators were included in the regression model one at a time. The authors note in the methods that “child studies are substantially more likely to have saliva rather than blood DNAm”. This raises concern that tissue type may confound the moderating effect of timing on the association between SES and biological aging. It would be helpful to see a forest plot displaying effect sizes stratified by both age group (children vs. adults) and tissue type (saliva vs. blood), but I understand that there may not be enough effect sizes available for this kind of comparisons.

Minor:
In the supplemental tables, some decimal separators are presented as commas instead of dots. Abbreviations (eg. ES, Esz, Qe,…) should be spelled out in the table notes.

Reviewer #3 (Remarks to the Author):

I have carefully reviewed the manuscript entitled “Social determinants of health and epigenetic clocks: Meta-analysis of 140 studies.” The topic is timely and the dataset is substantial, drawing on 140 studies and over 65,000 participants. The effort to synthesize evidence across different generations of epigenetic clocks is commendable and has potential value for researchers interested in social determinants of health. However, the manuscript in its current form suffers from important weaknesses in writing, conceptual framing, methodology, and interpretation. Several limitations are insufficiently acknowledged, and some conclusions are presented too strongly given the available evidence. My detailed comments are as follows:

1. The abstract does not meet academic standards; it is overly simplistic, lacks quantitative detail, and does not end with a clear conclusive statement.
2. The manuscript claims SES and race/ethnicity are the most powerful social determinants but does not provide a convincing rationale for prioritizing them over other determinants.
3. Multiple effect size metrics were converted into Pearson’s r; this process may involve extensive estimation and imputation, yet methodological transparency and sensitivity analyses are insufficient.
4. In the “adults’ DNAm with childhood SES” analysis, reliance on retrospective self-reports introduces recall bias and likely underestimates effect sizes; this limitation should be explicitly discussed.
5. All effect sizes for race/ethnicity come from U.S.-based cohorts, severely limiting generalizability, yet conclusions are drawn broadly.
6. Heterogeneity statistics (e.g., I^2, τ^2) are not consistently reported, which undermines evaluation of the pooled results.
7. Sex differences are discussed in the text but no subgroup analyses across childhood vs. adulthood are performed; such analyses could add important insights.
8. Writing contains repetition (e.g., page 12, first paragraph) and redundancy; the manuscript is excessively long and could be streamlined.
9. Deviations from the preregistered protocol are only placed in supplementary tables; they should be explicitly detailed in the main text and compared with preregistered plans.
10. No systematic risk-of-bias assessment of included studies is presented; this is a serious omission for a meta-analysis of observational data.
11. Numerous moderator and subgroup analyses are performed, raising the risk of false positives; no correction for multiple comparisons is provided.
12. The three-level meta-analytic model is appropriate but variance components (level-2, level-3) are not reported; cluster-robust variance estimation or leave-one-cohort-out checks are missing.
13. Publication bias is only assessed for SES associations; no equivalent tests are applied for race/ethnicity, and alternative methods (e.g., selection models, p-curve) are absent.

14. SES measures are highly heterogeneous (education, income, composite indices) yet are pooled together without sufficient stratification; this may dilute or obscure effects.
15. Adjustment for key confounders (e.g., smoking, BMI) varies across studies; the influence of inconsistent covariate control is not evaluated.
16. First-generation clocks sometimes indicate “deceleration” of biological age among Black individuals, opposite to second- and third-generation clocks; this anomaly is not explained or tested.
17. Children’s samples rely heavily on saliva or buccal tissue, yet these tissue-specific limitations are not fully considered when interpreting results as life-course effects.
18. Effect sizes derived from studies with small samples or imputed SDs are included without adequate robustness checks; analyses restricted to large-sample or directly reported metrics would be useful.
19. Intergenerational SES and mobility analyses are mentioned but not developed; given limited effect sizes, these results should be presented more cautiously.
20. The literature search only extends to February 2024, omitting recent 2024–2025 studies and new clocks; either the search should be updated or conclusions limited accordingly.
21. Interpretive language is often causal (“disadvantage accelerates aging”), though the evidence is correlational; stronger caution is necessary.
22. Data sharing could be improved; while scripts are said to be available in supplements, depositing standardized datasets and code in an open repository would enhance reproducibility.

Version 1:

Decision Letter:

Our ref: NATHUMBEHAV-25052414A

6th January 2026

Dear Dr. Raffington,

Thank you for submitting your revised manuscript "Social determinants of health and epigenetic clocks: Meta-analysis of 140 studies" (NATHUMBEHAV-25052414A). It has now been seen by the original referees and their comments are below. As you can see, the reviewers find that the paper has improved in revision. We will therefore be happy in principle to publish it in Nature Human Behaviour, pending minor revisions to satisfy the referees' final requests and to comply with our editorial and formatting guidelines.

We are now performing detailed checks on your paper and will send you a checklist detailing our editorial and formatting requirements within two weeks. Please do not upload the final materials and make any revisions until you receive this additional information from us.

Please do not hesitate to contact me if you have any questions.

Sincerely,

[redacted]

[redacted] and Team Leader (Magazine)
Nature Human Behaviour

Reviewer #1 (Remarks to the Author):

Thank you for your careful and responsive revision. I appreciate that each of the points I raised in the first round were considered and thoughtfully integrated into the manuscript. I do not have any additional substantive suggestions. Thanks for this wonderful contribution.

Reviewer #2 (Remarks to the Author):

The authors have addressed all my concerns and I would like to thank them for their thorough revisions.

Reviewer #3 (Remarks to the Author):

The authors have satisfactorily addressed all editorial and reviewer concerns. The revised manuscript demonstrates improved conceptual clarity, methodological rigor, and appropriately tempered interpretation, particularly regarding generational differences in epigenetic clocks, pediatric samples, and heterogeneity due to covariate and technical factors. I have no further substantive concerns and support acceptance of the manuscript in its current form.

Open Access This Peer Review File is licensed under a Creative Commons Attribution 4.0 International License, which permits use, sharing, adaptation, distribution and reproduction in any medium or format, as long as you give appropriate credit to the original author(s) and the source, provide a link to the Creative Commons license, and indicate if changes were made.
In cases where reviewers are anonymous, credit should be given to 'Anonymous Referee' and the source.
The images or other third party material in this Peer Review File are included in the article’s Creative Commons license, unless indicated otherwise in a credit line to the material. If material is not included in the article’s Creative Commons license and your intended use is not permitted by statutory regulation or exceeds the permitted use, you will need to obtain permission directly from the copyright holder.
To view a copy of this license, visit https://creativecommons.org/licenses/by/4.0/

Comments by Reviewer 1

1) **The authors provide a comprehensive meta-analysis of associations between various methylation-based indicators of biological aging with SES and race/ethnicity. They find associations between accelerated aging with lower SES and for Black and Latinx relative to White identifying groups. Associations tended to be larger for more recent measures, especially the two Dunedin indices and GrimAge. In addition, differences across age, sex, and technical factors were explored. It is very valuable to have these new meta-analytic estimates. The pre-registered analyses are well executed with only very minor deviations. I appreciate how well the results are reported in thorough tables and elegant and intuitive figures. I think this is a great contribution and I have only a few suggestions.**

   We thank Reviewer 1 for carefully reading our manuscript and for constructive suggestions that have helped improve its quality. Our responses follow (reviewer comments appear in bold):

2) **My primary concern is the framing of the "speedometers" outperforming the second-generation clocks as a key result. While this is technically true, it is driven by their specific outperformance of PhenoAge, as they rarely (if ever?) significantly outperform GrimAge. In prior studies, GrimAge tends to correlate more strongly with DunedinPACE than with PhenoAge. While I have no issue with framing the Dunedin indices as a distinctive generation of speedometers, I found a few of the claims to be too strong in light of this pattern of results. The incremental validity may well be related to the potency of the training phenotypes for both GrimAge and DunedinPACE/PoAm relative to the other (even second generation) measures rather than indexing pace as opposed to accumulation. Therefore, I found it slightly misleading to include the following in the Abstract: "The strongest associations were observed for a third generation of epigenetic clocks, sometimes referred to as "epigenetic speedometers", developed to predict the pace of aging"**

   Thank you for bringing this to our attention. We agree with the reviewer's interpretation of the data. We rewrote the abstract and discussion (see reply to comment 3) to moderate claims about the third-generation "speedometers" outperforming the second-generation clocks.

   Abstract, page 2:

   "Social determinants of health - such as socioeconomic status (SES) and race/ethnicity - strongly shape health and survival. Individuals with lower SES or marginalized identities experience earlier onset of age-related disease and shorter lifespans. DNA methylation (DNAm) based "epigenetic clocks" measure biological aging and are increasingly used to study social influences on health. However, it remains unclear which generations of clocks are most sensitive to social factors. We conducted a preregistered multilevel meta-analysis of 140 studies (N = 65,919; 1,065 effect sizes) testing associations of

SES and race/ethnicity with three generations of clocks. Associations were weakest for first-generation clocks (r = –0.03, p < .001) and stronger for second- (r = –0.11, p < .001) and third-generation clocks (r = –0.13, p < .001). Sex, tissue, and array minimally modified results, and publication bias was negligible. Findings indicate newer clocks are more responsive to social inequality."

3) **Similarly, I think the following overstates the pace versus accumulation distinction in the Discussion, given that neither Dunedin index had significantly larger associations than GrimAge in the analyses of childhood DNAm:**
"One interpretation of these data is that acceleration to aging processes caused by early adversity is immediately apparent in "speedometer" type third-generation clocks, but does not become apparent until later in life in second-generation clocks. Collectively, findings suggest that third-generation clocks, such as DunedinPACE, are the most promising tools for tracing environmental impacts on aging and health early in life"

   I'd suggest tempering the language that highlights second and third generation differences and providing some interpretation as to why GrimAge also appears to have strong predictive validity relative to other measures.

   Thank you for this suggestion. We agree that, while only SES-DunedinPoAm associations were significantly different from zero, the meta-analysis effect size was not significantly different from SES-GrimAge in children (confidence intervals for these effect sizes are large due to the relatively small number and size of studies contributing data to this analysis). In line with your suggestion, we have rewritten this section to temper our language and highlight these nuances.

   Discussion, page 11:

   "Collectively, these findings suggest that third-generation clocks such as DunedinPACE, alongside second-generation clocks like GrimAge, are promising tools for tracing environmental effects on aging early in life."

4) **Overall, I thought the Introduction was great – a concise and effective summary. My suggestions for it are minor. I think that saying epigenetic clocks are "among the most widely studied and best-validated predictors of healthy lifespan across a range of human populations" likely overstates their predictive power. Is there any evidence they outperform indices of aging like cognition, adaptive functioning, or inflammation in predicting mortality?**

   We have carefully considered this suggestion and have included references to studies that indicate that clocks have strikingly high AUCs for many different health outcomes, substantially outperforming other biomarkers such as single-analyte biomarkers like C-reactive protein.

Introduction, page 3:

"a family of machine-learning-derived DNA methylation (DNAm) algorithms known as "epigenetic clocks" are widely studied and well-validated biomarker predictors of healthy lifespan across a range of human populations (Balachandran et al., 2025; Higgins-Chen et al., 2022)."

5) **I thought more rationale could be given studying age differences. For instance, how does the concept of biological aging apply in childhood? Do developmental researchers think of things like early puberty as similar to biological age exceeding chronological age late in life?**

These are excellent, foundational question. We agree they deserve more attention in the main text.

We have integrated our rationale into the Introduction on page 3:

"*Research increasingly shows that epigenetic clocks are sensitive to socioeconomic, racial, and ethnic disparities (Aikins et al., 2025; Crimmins, 2020; G. H. Graf et al., 2022; Mastrotheodoros et al., 2023; Shen et al., 2023). Yet, many clocks exist, and they do not provide identical information. In fact, correlations across generations of clocks are modest (Drewelies et al., 2024; G. H. Graf et al., 2022). Key knowledge gaps include which clocks best capture social determinants of health, when socioeconomic exposures most affect biological aging, and whether effects differ by sex. Researchers need guidance on selecting clocks suited to testing these hypotheses and identifying life stages where associations are strongest. Theory and evidence suggests that aging begins at conception (Gladyshev et al., 2021; Kerepesi et al., 2021). However, how molecular damage and hallmarks of aging relate to early-life exposures remains unclear. Addressing these questions will improve the use of clocks in studies testing interventions across the life course.*"

And provide more context in the Discussion on page 11:

"While aging likely begins at conception (Gladyshev, 2016; Kerepesi et al., 2021), its relation to development remains unclear. Accelerated aging may divert resources from growth, or developmental activity itself may induce damage. Molecular epidemiology is now linking aging biomarkers to developmental processes, including pubertal timing (Aikins et al., 2025; deSteiguer et al., 2025). Because clocks were trained in adults with different blood composition and no active developmental programs, pediatric estimates may be less accurate and reflect both aging and development, which is why we should interpret them with caution."

6) **I'm curious how the authors would feel about framing hypotheses 4 and 5 as more supplemental, rather than as central as the first three. Because the analyses related to sex differences and technical factors were hampered by methodological issues outside of the authors' control**

(I appreciate the transparency in noting this), I found myself less compelled and putting less stock into them.

Thank you for this helpful suggestion. We agree that Hypotheses 4 and 5 are better positioned as supplemental to the main analyses. In the revised manuscript, we have combined these hypotheses into a single subsection and discuss the findings with reduced emphasis. We have also added additional analyses on the included covariates to provide further context (see response to Reviewer 3, Comment 16).

See Introduction, page 4:

"Fourth, we explored potential factors modifying the associations, such as sex and technical factors (tissue type, array platform, included covariates like BMI and smoking), and whether there was evidence of publication bias."

See Results, page 8:

"Factors potentially modifying the magnitude of the association between SES-epigenetic clocks and Race/Ethnicity-epigenetic clocks"

7) **Similarly, I thought the presentation of methods and results was excellent. I had one point of confusion that I think the authors could clear up in a revision, and one idea to consider.**

I had a hard time following the number of effect sizes. I am probably missing something, but I summed 908 effect sizes in the SES analyses and an additional 109 for the race/ethnicity analyses. This sums to 1,017, which is less than the reported 1,065. In addition, the authors write "DNAm collected in adulthood (83% of effect sizes) is more common than DNAm in childhood (15%)", so I was curious why this didn't sum to 100%. In the figure, it looks 113 of 908 SES effect sizes were from childhood DNAm samples (12.4%). I think a sentence or two that clarifies what all went into the 1,065 figure (and how it is split up) would be helpful.

Thank you for pointing this out. We understand how this could have been confusing. In the original version, we did not clearly specify how we handled the effect sizes representing change (i.e., the associations between changes in SES and changes in clocks) or how we assessed biweight midcorrelations (described in the Supplementary Materials). In the revised manuscript, we have added a sentence clarifying the breakdown of these effect sizes and updated the corresponding figure to highlight this.

Results, 4:

"Of the 1,065 extracted effect sizes, 1,017 were included in the main analyses examining associations between epigenetic-clock–based biological aging and social determinants of health: 908 for socioeconomic status (SES) and 109 for race/ethnicity (68 Black–White and 41 Latinx–White comparisons). The

remaining 48 effect sizes were analyzed separately, including 34 assessing changes in biological aging and/or SES over time and 14 using biweight midcorrelation (bicor) estimates."

Results, page 5:

"We note that DNAm collected in adulthood (87.6% of effect sizes) is more common than DNAm in childhood (12.4%).

Supplemental Figure 2:
(we moved this from the main manuscript to the supplemental figures so we made space for the new figure requested in comment 8).

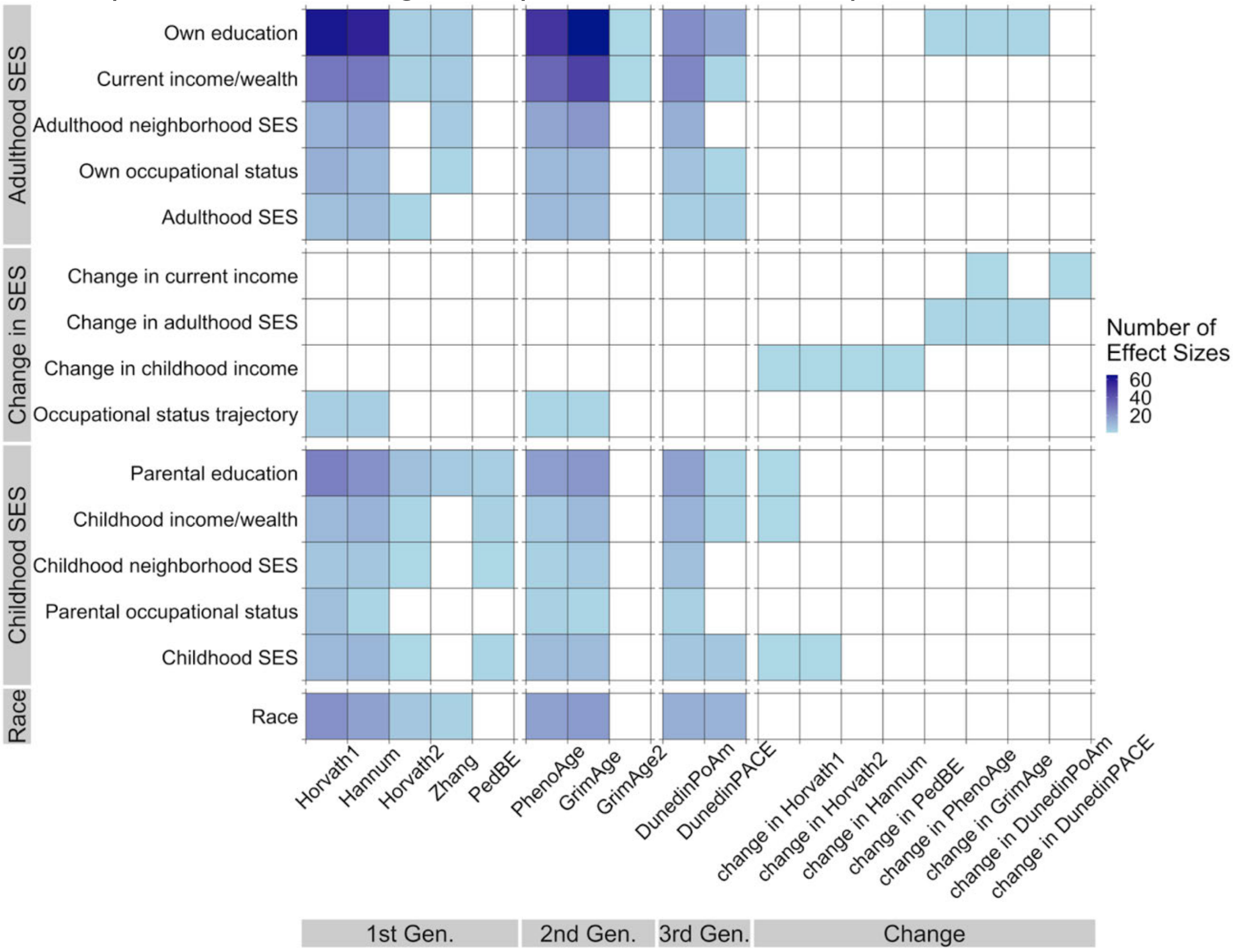

8) **I thought Supplemental Table 3C summarized many of the key results in a nice and concise manner. I found myself going back to it more than the other tables. I wonder what the authors would think about making it more central and perhaps including it in the main text.**

We think this is a great suggestion and we have re-located the table from the supplement to the manuscript and turned it into a more visually appealing figure (instead of a table):

Figure 5, page 32:

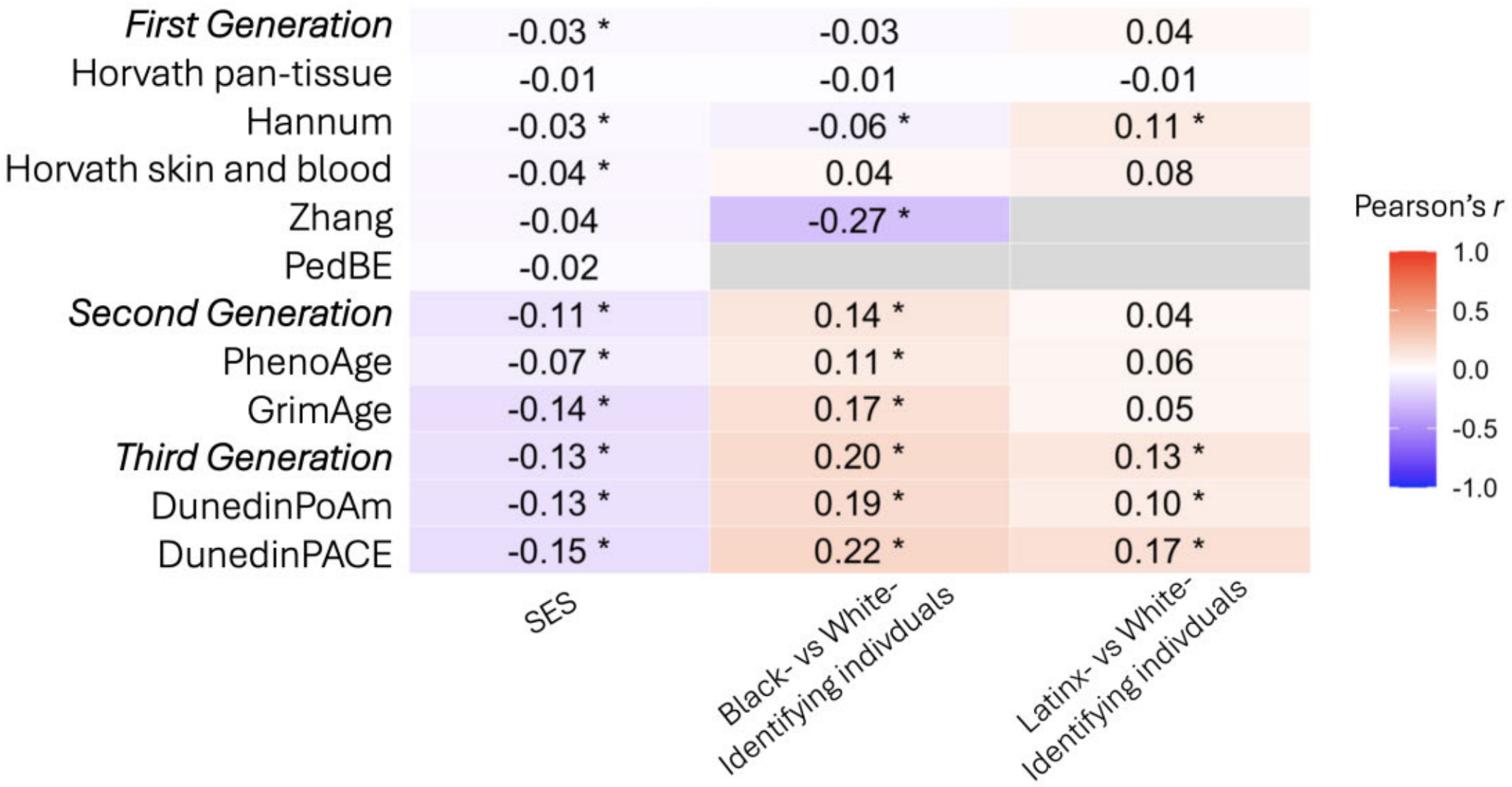

Figure 5. Pearson's r values for the associations between SES/race/ethnicity and DNAm generations and measures of biological aging. *Note:* Estimates reflect Pearson's r to compare estimates across social determinants of health. Estimate's in Cohen's d can be found in Figure 4.

9) **My only suggestion for the Discussion is also minor, aside from the concern discussed above. I thought more attention could have been given to the null finding for repeated methylation measurements. I'd be curious what the other reviewers and editors think. While I understand there were fewer studies and results were less precise, I think these analyses are central to the endeavor as outlined in the Introduction (i.e., how responsive is biological aging to environmental change?). I think some discussion of the possibility that longitudinal associations may be null is worth exploring.**

While we agree that repeated methylation measures offer an important and insightful approach to understanding how biological aging responds to environmental change, our meta-analysis revealed that very few studies currently include such data. On average, only two effect sizes per clock were available, making it statistically difficult to distinguish true null findings from limited power. In line with your feedback, we now emphasize the value of these longitudinal analyses and highlight them as a promising direction for future research.

See response to Reviewer 2, Comment 3.

Comments by Reviewer 2

1) **This meta-analysis investigates the association between social determinants of biological aging (socioeconomic status (SES) and race/ethnicity) and biological aging as measured by epigenetic clocks. In addition, the authors explore potential moderators (e.g.. clock generation, timing, technical factors) of these associations. The meta-analysis uses summarized data from 140 studies comprising 65,919 participants. Lower SES and marginalized race/etnicity were associated with accelerated biological aging. The association of SES was strongest for second- and third-generation measures, and only third-generation clocks indicated association in children. To the best of my knowledge, there are no meta-analyses comprehensively examining the associations between SES/race and biological aging using DNAm-based measures from all three generations. Overall, the manuscript is well-written and presents a substantial amount of interesting and valuable information. My specific comments and concerns are listed below:**

   We would like to thank Reviewer 2 for the positive assessment of our manuscript, thank you for your time and effort you put into reading our work. Our response follows (the reviewer's comments are in bold).

2) **Abstract**
   Does the phrase "Effects of sex and technical factors were minimal…" refer to modifying effects of these factors? This should be clarified.

   Thank you for highlighting this, it is indeed modifying effect which we have added to the abstract. We addressed this in the reply to Reviewer 1, comment 1.

3) **Introduction:**
   The term 'determinant' may imply a causal relationship. However, to my knowledge, there is no conclusive evidence establishing a causal effect of SES and biological aging. The present meta-analysis uses effect sizes from observational studies. It is also plausible that individuals with better underlying health are more likely to attain higher SES. I believe it would be appropriate at least briefly to acknowledge the potential confounding role of factors such as genetic influences in these associations.

   In the revised manuscript, we have ensured that causal language is avoided when describing the associations between SES and biological aging and added a sentence to the Discussion to emphasize more research is necessary to move from observational studies to causal interpretation. We note that the term *"determinant"* is now used only in the context of *"social determinants of health,"* which is a well-established conceptual framework rather than a causal claim (WHO, 2025). We therefore retained this

terminology when referring to that broader framework but refrained from using it to describe SES–clock associations.

Discussion, page 12:

"There is now abundant correlational evidence linking a range of socially stratified exposures with epigenetic clock measures of biological aging (e.g., environmental toxicants, access to healthcare and stress) (Belsky & Baccarelli, 2023; Carroll & Prather, 2021; Febbraio, 2017; Mateo & Williams, 2021; Peters et al., 2021; Raffington & Belsky, 2022). Quasi-experimental studies further provide causal evidence that early-life conditions—poverty, in utero undernutrition from famine, and lower educational attainment—accelerate later-life epigenetic aging (M. Cheng et al., 2024; G. H. J. Graf et al., 2024; Schmitz & Duque, 2022). However, few studies have included repeated measures of DNAm to examine how changes in environmental conditions correspond to changes in biological aging. Ongoing trials are probing epigenetic clock sensitivity to randomized interventions, including behavioral programs (Bischoff-Ferrari et al., 2025; Waziry et al., 2023) and cash transfers. Our results can guide these studies' choice of outcome measures."

4) **Are the authors aware of any previous meta-analyses that have assessed the associations between socioeconomic status (SES) or race/ethnicity and biological aging as measured by epigenetic clocks?**

Previous meta-analyses and systematic reviews have examined associations between socioeconomic status (SES), race/ethnicity, and epigenetic clocks; however, these studies have had important limitations. Some conducted systematic reviews without meta-analytic synthesis (Raffington & Belsky, 2022), while others focused on a narrow set of clocks (e.g., only DunedinPACE , Sugden et al., 2023), or first- and second-generation clocks but not third-generation (Oblak et al., 2021). Others restricted their scope to specific SES indicators (e.g., neighborhood characteristics, Jackson et al., 2023) or included only certain effect size types (e.g., studies reporting standardized betas, Chervova et al., 2024). In contrast, our study is the first to meta-analyze associations across the most commonly used first-, second-, and third-generation clocks, spanning both child and adult samples, providing the most comprehensive synthesis to date.

We have added the following sentence to our introduction:

Discussion, page 10:

"Previous systematic reviews and meta-analyses examining socioeconomic and racial/ethnic differences in epigenetic aging have been limited in scope (Chervova et al., 2024; Oblak et al., 2021; Raffington & Belsky, 2022; Sugden

et al., 2023), whereas our study is the first to quantitatively synthesize evidence across first-, second-, and third-generation clocks, integrating findings from both child and adult samples to provide a comprehensive view of how social determinants relate to biological aging across the lifespan.”

5) **Discussion: If studies based on the latest EPICv2 platform were excluded, this should be acknowledged in the limitations section.**

   We have integrated a reply to this in comment 8.

6) **It would be appropriate to acknowledge that the meta-analysis was limited to studies from high-income countries thus potentially limiting the generalizability of findings.**

   We agree, we have now integrated this acknowledgement in our discussion:

   See Discussion, page 12:

   “An urgent priority is to develop and validate biological aging algorithms that are inclusive and globally representative. This will require expanding data collection beyond predominantly Western, high-income cohorts to encompass participants from varied socioeconomic and geographic contexts, ensuring that epigenetic measures accurately reflect aging processes worldwide.”

7) **The authors conclude that ”In children, only the third -generation speedometers showed consisted associations”, and suggest that their findings provide guidance for selecting epigenetic clocks for future studies. However, in the included child studies, the DNAm was primarily measured using saliva samples. It remains unclear whether the superior performance of the third-generation speedometers would hold if blood-based DNAm data were used instead in children.**

   Thank you for this thoughtful comment. We have addressed this issue in comment 9.

   We agree that tissue differences represent a common challenge in epigenetic research, and indeed, a large amount of the child studies in our meta-analysis relied on saliva-derived DNAm data. Because blood collection in children is often difficult and invasive, future studies are likely to continue relying on saliva samples. Prior work has demonstrated moderate-to-high cross-tissue reliability for the clocks included in our analyses between blood and saliva (Zarandooz & Raffington, 2025), suggesting that this limitation is unlikely to substantially affect the observed SES–clock associations, even though some signal is likely to be lost. We have now incorporated this clarification into the manuscript as follows:

   Discussion, page 11:

“Fourth, we found little evidence that sex, array, tissue type, or cell-count correction moderated SES or racialized differences in epigenetic aging. Still, whether these factors influence SES–clock associations warrants further study. Future work should directly compare associations between females and males, as our meta-analysis could only contrast studies that were mostly male, mostly female, or sex-balanced. Among adults, men typically show older biological age and faster aging than women, whereas in adolescence, girls show older biological age and faster aging, consistent with earlier pubertal timing (deSteiguer et al., 2025; Faul et al., 2023; Raffington et al., 2023). Sex-specific racial/ethnic disparities remain underexplored, though our results suggest that social disparities in GrimAge and DunedinPACE are similar across sexes. Few studies have tested whether SES–clock associations differ by tissue type in children, but prior work shows moderate-to-high correspondence between blood and saliva after cell adjustment(Zarandooz & Raffington, 2025). Although some signal may be lost in saliva, SES associations with GrimAge, DunedinPoAm, and DunedinPACE appear robust to such technical variation. Measuring DNAm in both tissues will clarify how cellular context shapes clock associations in childhood.”

8) **Methods:**
Based on the data provided in the supplement, there appears to be an inconsistency in the coding of the variable ‘Array’. According to the instructions, the categories are defined as: 1=EPIC, 2=EPIC v2, 3=450k, 0=n/a. However, the dataset contains values 0,1,3,4. In Table 3, the chip versions are 450k, EPIC, 450k and EPIC, n/a. Therefore, it is unclear whether studies using EPIC v2 platform were included in the meta-analysis, and if so, whether they were analysed together with studies using EPIC v1. This should be clarified. Additionally, it would be helpful to know whether the authors examined potential differences in associations across these platforms, as there are EPIC v2 has been shown to perform differently from earlier version (Tay et al., 2025).

Reference:
Tay, J. H., Chew, Y. E., Wang, W., Lim, Z. M., Guan, L., Dorajoo, R., Kennedy, B. K., Brooke, R., Gordevicius, J., Horvath, S., Sandalova, E., & Maier, A. B. (2025). DNAm age differences between infinium methylationEPICv1 vs EPICv2 in buffy coat, PBMC, and saliva samples. Communications Biology, 8(1). https://doi.org/10.1038/s42003-025-08021-y

Thank you for noting this inconsistency. We indeed preregistered the inclusion of EPIC v2 arrays, recognizing that recent evidence indicates differences across array platforms (Tay et al., 2025). However, upon reviewing the final set of included studies, we found that none of the included study used the EPIC v2 platform, likely due to its recent release. We therefore recoded the variable to reflect only the arrays actually represented in the meta-analysis (EPIC v1, 450k, mix of both), and the public dataset has been updated accordingly to remove EPIC v2 from the coding scheme.

We have included this in the results as following:

Results, page 9:

“We preregistered analyses comparing EPIC v2 and EPIC v1, but none of the studies in our meta-analysis used the EPIC v2 array because it was only recently released .”

9) **Statistical analysis**
If I understood correctly, the moderators were included in the regression model one at a time. The authors note in the methods that “child studies are substantially more likely to have saliva rather than blood DNAm”. This raises concern that tissue type may confound the moderating effect of timing on the association between SES and biological aging. It would be helpful to see a forest plot displaying effect sizes stratified by both age group (children vs. adults) and tissue type (saliva vs. blood), but I understand that there may not be enough effect sizes available for this kind of comparisons.

We appreciate the reviewer’s thoughtful comment. As noted, saliva samples were primarily available in child studies, and the effect sizes across specific clocks were generally small, making separate forest plots for children and adults uninformative. Moreover, because saliva-based effect sizes in adults were very limited, we were unable to test moderation by tissue type for the adult SES–DNAm associations.

However, we conducted an additional exploratory analysis for the child SES–DNAm associations, where both tissue types (blood and saliva) were more evenly represented. Due to the limited number of effect sizes per specific clock, moderation analyses were only feasible for the Horvath pan-tissue, Hannum, and PoAm clocks (consistent with our preregistered criterion of >5 effect sizes for moderation analyses). None of these analyses revealed significant differences between blood and saliva. This information has now been added to the *Results* section

Results, page 9:

“Because saliva samples were primarily available in child studies, we conducted an exploratory analysis to examine whether tissue type moderated the association between childhood SES and children’s DNAm. Only Horvath pan tissue , Hannum, and DunedinPoAm clocks had sufficient effect sizes across both tissue types to allow moderation testing, and none showed significant differences between blood and saliva (see Supplemental Table 4C).”

10) **Minor:**
In the supplemental tables, some decimal separators are presented as

commas instead of dots. Abbreviations (eg. ES, Esz, Qe,…) should be spelled out in the table notes.

Apologies for these inconsistencies, we have spelled out all abbreviations in the supplementary table notes.

Comments by Reviewer 3

1) **I have carefully reviewed the manuscript entitled "Social determinants of health and epigenetic clocks: Meta-analysis of 140 studies." The topic is timely and the dataset is substantial, drawing on 140 studies and over 65,000 participants. The effort to synthesize evidence across different generations of epigenetic clocks is commendable and has potential value for researchers interested in social determinants of health. However, the manuscript in its current form suffers from important weaknesses in writing, conceptual framing, methodology, and interpretation. Several limitations are insufficiently acknowledged, and some conclusions are presented too strongly given the available evidence. My detailed comments are as follows:**

   We thank Reviewer 3 for carefully reading the manuscript and for the constructive suggestions that help improve its quality. Our response follows (the reviewer's comments are in bold).

2) **The abstract does not meet academic standards; it is overly simplistic, lacks quantitative detail, and does not end with a clear conclusive statement.**

   The abstract was originally written in the style of other *NHB* manuscripts, aiming for widely accessible scientific communication. In response to your feedback, we have revised it to include additional quantitative details and a clearer concluding statement (see our reply to Reviewer 1, comment 1).

3) **The manuscript claims SES and race/ethnicity are the most powerful social determinants but does not provide a convincing rationale for prioritizing them over other determinants.**

   We focused on socioeconomic status (SES) and race/ethnicity because these determinants are consistently identified as the most pervasive and fundamental social drivers of health inequities across populations. Both shape access to resources, exposure to chronic stressors, and structural disadvantage that cumulatively influence disease risk and lifespan (Cheng et al., 2015; Williams et al., 2019; WHO, 2025). Other factors such as gender, neighborhood, social context, national policy are also important to consider in future research. Their effects can intersect with pathways through which SES and race/ethnicity exert their effects. We have revised the manuscript to clarify this rationale and added references to support the prioritization of these two determinants.

We added a sentence to the introduction:

Introduction, page 3:

"These factors are considered fundamental because they shape exposure to a wide range of health-relevant conditions, including access to resources, environmental hazards, and chronic stress (Cheng et al., 2015; Williams et al., 2019; WHO, 2025)."

4) **Multiple effect size metrics were converted into Pearson's r; this process may involve extensive estimation and imputation, yet methodological transparency and sensitivity analyses are insufficient.**

We thank the reviewer for the opportunity to clarify this point. Conversion of diverse effect size metrics into Pearson's *r* is a standard practice in meta-analysis and is consistent with prior work published in *Nature Human Behaviour* and in quantitative reviews of SES and epigenetic clocks (Oblak et al., 2021; Raffington & Belsky, 2022; Swaryandini et al., 2025). The specific formulas used for each type of conversion are detailed in the Methods section and the corresponding R code will be available upon publication. In addition, we provide a supplementary table listing all original effect sizes and the converted *r* values, allowing readers to reproduce or apply alternative conversion methods if desired. Together, these steps ensure full methodological transparency and confirm that our findings are robust to standard effect size conversions.

5) **In the "adults' DNAm with childhood SES" analysis, reliance on retrospective self-reports introduces recall bias and likely underestimates effect sizes; this limitation should be explicitly discussed.**

We agree with the reviewer that reliance on retrospective reports of childhood SES may introduce recall bias, which could attenuate associations with DNAm measures in adulthood. We have now added this limitation to the Discussion, see our reply to Reviewer 2, comment 3.

6) **All effect sizes for race/ethnicity come from U.S.-based cohorts, severely limiting generalizability, yet conclusions are drawn broadly.**

Thank you for emphasizing this caution, we have now included it in our discussion. See reply to comment 11.

7) **Heterogeneity statistics (e.g., I^2, τ^2) are not consistently reported, which undermines evaluation of the pooled results.**

Thank you for this suggestion. In line with best practices outlined by Wibbelink and Assink (2016), we now report heterogeneity statistics (Qe, omnibus test and variance at each level) for all main analyses. These

statistics have been added to the supplementary tables to allow full evaluation of the pooled effects.

8) **Sex differences are discussed in the text but no subgroup analyses across childhood vs. adulthood are performed; such analyses could add important insights.**

   We appreciate this suggestion. Unfortunately, too few studies in childhood samples reported sex-stratified results, as most included mixed-sex samples without providing separate estimates for boys and girls. As a result, subgroup analyses across childhood versus adulthood could not be reliably performed. However, our overall analyses including both child and adult samples showed little evidence for sex differences in the associations between SES or race/ethnicity and epigenetic clocks. We now clarify this in the revised manuscript.

   Results, page 9:

   "We did not probe sex moderation for race/ethnicity, nor specifically in childhood samples, because there were too few studies with sex-imbalanced cohorts."

9) **Writing contains repetition (e.g., page 12, first paragraph) and redundancy; the manuscript is excessively long and could be streamlined.**

   We critically reread the manuscript and revised it to streamline the text and eliminate redundancies, particularly in the abstract, discussion, and the first paragraph on page 12. The main text is now <5000 words long, which is in line with NHB guidelines.

10) **Deviations from the preregistered protocol are only placed in supplementary tables; they should be explicitly detailed in the main text and compared with preregistered plans.**

   We now explicitly note any deviations from the preregistration throughout the manuscript. We note that we made very few deviations to our preregistered plan and mainly added additional analyses to further improve our understanding of results.

   Methods, page 17:
   "We conducted moderator analyses when effect-size heterogeneity was present and at least five effect sizes were available per category. To be more inclusive, we deviated from our preregistration (which required five studies) and instead used a threshold of five effect sizes per category. We also prioritized the timing of DNAm measurement as the most empirically meaningful moderator."

11) **No systematic risk-of-bias assessment of included studies is presented; this is a serious omission for a meta-analysis of observational data.**

Thank you for pointing this out. We have now included the following risk-of-bias assessment in our revised manuscript. In line with previous meta-analyses on SES-epigenetic clocks (Ryan et al., 2020; Oblak et al., 2021; Cherova et al., 2024), we prepared funnel plots, performed Egger's regression test, and ran trim-and fill analyses to assess publication bias (see Supplemental Table S1D).

Additionally, for risk-of-bias assessment, we re-ran analyses excluding non-peer-reviewed papers, excluding meta-analyses, and excluding effect sizes from the largest effect size contributing study the Health and Retirement study cohort respectively. We also re-ran analyses excluding effect sizes with small sample sizes (< 100), to ensure robustness of results. Lastly, we ran Leave-One-Cohort-Out analyses with 19 other influential cohorts. We selected cohorts that were included in at least 3 studies or contributed at least 25 effect sizes. We then re-ran 19 analyses excluding these cohorts one by one. Overall, our results remained unchanged in these robustness checks.

We included this in the Results on pages 4-5:

"We tested for publication bias in SES–clock associations using funnel plots, Egger's test, and trim-and-fill analyses. No evidence of bias was found: plots were symmetric (Supplemental Figure 2), Egger's tests were non-significant, and trim-and-fill minimally affected magnitude of effect sizes (Supplemental Table 1B-C). Sensitivity analyses excluding HRS data, meta-analyses, preprints, effect sizes with small sample sizes (n < 100), and leave-one-cohort- out tests produced negligible changes in effect sizes (r = 0–0.02, see Supplemental Figures 3-6,17 & Supplemental Tables 1D-G), confirming robustness of results."

And Results, page 8:

"We then examined potential publication bias in associations between race/ethnicity and epigenetic clock measures using funnel plots, Egger's regression, and trim-and-fill analyses. Funnel plot's and Egger's test showed asymmetry for the Hannum and GrimAge clocks in Black–White comparisons (See Supplemental Figure 9, Supplemental Table 3C), but trim-and-fill corrections produced minimal changes in effect size estimates (see Supplemental Table 3D). For Latinx–White comparisons, Horvath Pan-Tissue, GrimAge, and DunedinPACE indicated possible bias (see Supplemental Figure 10, Supplemental Table 3E), and trim-and-fill results showed differences in magnitude of the effect sizes (see Supplemental Table 3F). However, results were uncertain due to few studies and high heterogeneity (≥80%). Excluding preprints or small-sample effect sizes minimally altered results by 0–0.02 in Cohen's d (see Supplemental Figures 11-12, Supplemental Tables 3G-J). Removing HRS showed

stronger alterations Black–White effects by ~ 0.03–0.21 and Latinx–White by ~0.04–0.16 (see Supplemental Figure 13, Supplemental Tables 3K-L), as HRS contributed most of the race/ethnicity effect sizes. Leave-one-cohort-out tests for SES–clock links changed d by 0–0.02, occasionally 0.04 (See Supplemental Figures 18-19).

See Methods, page 18:

Risk-of-Bias

"We evaluated potential risk of bias using multiple complementary approaches. We generated funnel plots and conducted Egger's regression tests to assess systematic bias and applied trim-and-fill analyses to estimate the influence of potentially missing studies. To test the robustness of our findings, we re-ran all meta-analyses (a) excluding non–peer-reviewed studies, (b) excluding prior meta-analyses, (c) excluding the largest contributing cohort (the Health and Retirement Study), and (d) excluding effect sizes derived from small samples (n < 100). In addition, we conducted Leave-one-cohort-out *(LOCO) analyses for 19 influential cohorts - defined as those included in ≥3 studies or contributing ≥25 effect sizes - by sequentially removing each cohort and re-estimating the associations."*

12) Numerous moderator and subgroup analyses are performed, raising the risk of false positives; no correction for multiple comparisons is provided.

Thank you for raising this important point. We have now applied multiple comparison corrections to our analyses. The main results remain significant after correction. For the moderator analyses, all findings hold after correction except for the sex difference in the Hannum clock, which no longer reaches significance. This has been noted in the revised text.

Results page 8:
"(see Supplemental Table 4A, sex differences for Hannum did not survive correction for multiple comparison).

Results 12:
"All findings held after correction for multiple comparisons."

13) The three-level meta-analytic model is appropriate but variance components (level-2, level-3) are not reported; cluster-robust variance estimation or leave-one-cohort-out checks are missing.

Thank you for this remark, we have now added variance components to our tables of the main analyses in the supplemental tables, see reply to comment 11 on leave-one-cohort out analyses which we have included as well in the revised manuscript.

14) Publication bias is only assessed for SES associations; no equivalent tests are applied for race/ethnicity, and alternative methods (e.g., selection models, p-curve) are absent.

Thank you for this remark, we have added information on publication bias for race although it should be noted that due to the smaller number of effect sizes and high heterogeneity in the race-clock associations these analyses might be affected. See reply to comment 11.

15) SES measures are highly heterogeneous (education, income, composite indices) yet are pooled together without sufficient stratification; this may dilute or obscure effects.

We agree that socioeconomic status (SES) can be operationalized through diverse indicators such as education, income, and composite indices. However, these measures are conceptually related and moderately to strongly correlated, and prior systematic reviews and meta-analyses examining SES and epigenetic aging have also aggregated across SES indicators (Chervova et al., 2024; Oblak et al., 2021). In line with this literature and to maximize comparability across studies, we therefore analyzed SES measures collectively in the main analyses. We acknowledge this heterogeneity as a limitation and now explicitly note it in the revised manuscript:

See Table 1, page 13:

“Although SES was operationalized differently across studies (e.g., education, income, composite indices), these indicators are conceptually and empirically related and were therefore analyzed together, consistent with prior meta-analytic work on SES and epigenetic aging (Chervova et al., 2024; Oblak et al., 2021).”

16) Adjustment for key confounders (e.g., smoking, BMI) varies across studies; the influence of inconsistent covariate control is not evaluated.

We appreciate this important comment. Indeed, key behavioral and physiological factors such as smoking and BMI may confound associations with epigenetic clocks. To address the reviewer’s concern about inconsistent covariate control, we undertook a number of steps. In the revised manuscript, we added a column to our data extraction table indicating whether each effect size was adjusted for covariates, and if so, which ones. We then conducted moderator analyses to examine whether associations between (1) SES and epigenetic clocks and (2) race/ethnicity and epigenetic clocks differed depending on covariate inclusion. Specifically, we compared studies including ≤5 versus >5 covariates, and we separately tested whether including smoking or BMI as covariates moderated the associations.

Results, page 9:

"Covariates
Lastly, we examined whether the number and type of covariates (smoking, BMI) moderated associations of SES with epigenetic clocks. Across studies, covariate counts ranged from 0 to 37; 643 effect sizes included >5 covariates, 420 adjusted for smoking, and 348 adjusted for BMI. Moderator analyses indicated that the SES–DunedinPoAm association was stronger in models with fewer covariates (≤5: r = −0.17) than in those with more (>5: r = −0.11), whereas PhenoAge, GrimAge, and DunedinPACE were not significantly affected by covariate count (Supplemental Table 4H-I). Adjusting for smoking attenuated SES–GrimAge (−0.16 to −0.07) and SES–DunedinPoAm (−0.17 to −0.06) associations, but did not effect other SES-clock associations (Supplemental Table 4H-I). Similarly, adjusting for BMI attenuated SES–GrimAge (r −0.15 to −0.07) and SES–DunedinPoAm (−0.16 to −0.06) associations, but did not affect other SES–clock associations (Supplemental Table 4H-I). Examining the effects of covariates on the association between race/ethnicity and epigenetic clocks was limited by the number effect sizes, but we did find a reduced effect size for the Black-White on clocks in Hannum (Cohen's d −0.05 to 0.02) when smoking was included as covariate, with no effects on other associations (Supplemental Table 4J-K). All findings held after correction for multiple comparisons."

Conclusion, page 12:

"Fifth, future research should clearly justify covariate selection and, where feasible, report effect sizes with and without key adjustments, as our analysis was constrained by limited reporting across studies. Although adjusting for smoking or BMI appears to attenuate some SES–clock associations, these findings should be interpreted cautiously, because these covariates were typically included alongside numerous others, potentially obscuring their specific contributions."

See Table 3, page 18:

Moderator	Type	Measure	Levels
Covariates	*Categorical*	*If covariates were included in the model*	*<5 covariates versus >5 covariates included*
			Smoking included / not included as covariate
			BMI included/not included as covariate

17) First-generation clocks sometimes indicate “deceleration” of biological age among Black individuals, opposite to second- and third-generation clocks; this anomaly is not explained or tested.

Generally, first generation clocks do not show significant differences for black- versus white- identifying individuals, that is why we are not interpreting these results on the specific clock level. Additionally, the Zhang clock, is based on very small effect sizes, which we added a remark of caution to in the results section.

Results, pages 7-8:
“While slight deceleration was observed for the Hannum and Zhang clocks, which primarily index chronological age, the second-generation clocks that capture morbidity- and mortality-related biological processes indicated faster aging among Black compared to White participants, consistent with established health disparities”

18) Children’s samples rely heavily on saliva or buccal tissue, yet these tissue-specific limitations are not fully considered when interpreting results as life-course effects.

We have addressed this comment in reply to Reviewer 2, comment 7 and 9.

19) Effect sizes derived from studies with small samples or imputed SDs are included without adequate robustness checks; analyses restricted to large-sample or directly reported metrics would be useful.

Thank you for this suggestion, we have now included sensitivity analyses excluding studies with small sample sizes. See our response to comment 11.

20) Intergenerational SES and mobility analyses are mentioned but not developed; given limited effect sizes, these results should be presented more cautiously.

Indeed, more caution is warranted here. We rewrote part of the results section, and added a notion of caution to the discussion too:

Results, page 7:

“We found that individuals whose parents and themselves grew up in low SES had accelerated biological aging compared to individuals whose parents and themselves grew up in high SES, although this conclusion should be interpreted with caution considering the limited effect sizes”

Discussion section see response to reviewer 2, comment 3.

21) **The literature search only extends to February 2024, omitting recent 2024–2025 studies and new clocks; either the search should be updated or conclusions limited accordingly.**

We have replied to this comment in the letter to the Editor on page 1, and have integrated it in the Results section:

See Results, page 10:

"To ensure our findings reflect the most current evidence, we conducted an updated systematic search covering studies published from February 2024 through September 2025. Following our preregistered protocol, we identified 280 records. After title and abstract screening, we reviewed 67 full-text articles; resulting in 33 studies after excluding studies based on inclusion criteria (comprised of 21 adult samples and 14 adolescent/child samples; four drew on cohorts included in our prior synthesis (e.g., HRS, Framingham Heart Study)).

Overall, results aligned with our meta-analysis (see Supplemental Table 5). First-generation clocks showed little to no association with SES or race/ethnicity, whereas later-generation clocks (GrimAge, PhenoAge, DunedinPACE) yielded more consistent associations. Lower compared to higher SES and Black compared to White racial identity was linked to faster aging. Effect sizes for second- and third-generation clocks in the new studies fell within our meta-analytic range (SES: r –0.12 to –0.20; race: d 0.12–0.25). Studies examining specific SES indicators (e.g., spousal or grandparent education) produced more variable findings. PhenoAge had the least consistent results, with several null associations for SES or race, consistent with our observation that it appears less sensitive than GrimAge or DunedinPACE. Taken together, the updated search supports the robustness of our meta-analytic conclusions."

See Discussion, page 10:

"Since our initial search, a growing number of studies have documented associations between SES, racial/ethnic disparities, and epigenetic clocks, which we synthesized in our systematic review. Taken together, the updated search supports the robustness of our meta-analytic conclusions."

22) **Interpretive language is often causal ("disadvantage accelerates aging"), though the evidence is correlational; stronger caution is necessary.**

We thank the reviewer for this important observation. We have thoroughly revised the manuscript to temper any language that could be interpreted

as causal. And we also explicitly acknowledged this limitation in the Discussion.

See response to Reviewer 2, Comment 3.

23) Data sharing could be improved; while scripts are said to be available in supplements, depositing standardized datasets and code in an open repository would enhance reproducibility.

We agree that open data and code sharing enhance reproducibility. To address this, we will deposit all R scripts and standardized datasets in an openly accessible GitHub repository and include the repository link in the Methods section upon publication. We have also added our data to the supplementary tables so everything is documented in one supplementary datafile (supplemental Table 7 and 8).

Methods, page 12:

"The full meta-analytic dataset and analytic scripts are available in Supplemental Table 7 and 8 and on our Github [link available upon publication]."

Reviewer Comments

Reviewer #1:
Remarks to the Author:
Thank you for your careful and responsive revision. I appreciate that each of the points I raised in the first round were considered and thoughtfully integrated into the manuscript. I do not have any additional substantive suggestions. Thanks for this wonderful contribution.

Reviewer #2:
Remarks to the Author:
The authors have addressed all my concerns and I would like to thank them for their thorough revisions.

Reviewer #3:
Remarks to the Author:
The authors have satisfactorily addressed all editorial and reviewer concerns. The revised manuscript demonstrates improved conceptual clarity, methodological rigor, and appropriately tempered interpretation, particularly regarding generational differences in epigenetic clocks, pediatric samples, and heterogeneity due to covariate and technical factors. I have no further substantive concerns and support acceptance of the manuscript in its current form.

We would like to thank the editor and reviewers for their time, careful evaluation, and constructive feedback throughout the review process.

Summary main findings

A meta-analysis of 140 studies (~66,000 participants) shows that social inequality is linked to faster biological aging, with stronger associations observed for newer DNA methylation epigenetic clocks than earlier generations.

Hashtags:
#BiologicalAging; #DNAmethylation; #Epigenetics; #MetaAnalysis;
#SocialDeterminantsOfHealth

Twitter
@YayoukWillems
@AikinsMuna
@QiaoWu4
@laraffington
@Danbelsky

Bluesky
@laraffington.bsky.social
@danbelsky.bsky.social
